# The Effect of Indoor Daylight Levels on Hospital Costs and Length of Stay of Patients Admitted to General Surgery

**DOI:** 10.3389/fpubh.2021.678941

**Published:** 2022-01-20

**Authors:** Xiawei Li, Jianyao Lou, Zheping Yuan, Aiguang Shi, Ning Wang, Lin Zhou, Mingchen Zhao, Fanghe Ye, Zikun Pan, Yulian Wu

**Affiliations:** ^1^Department of Surgery, Second Affiliated Hospital, Zhejiang University School of Medicine, Hangzhou, China; ^2^Key Laboratory of Cancer Prevention and Intervention, China National Ministry of Education, Cancer Institute, Second Affiliated Hospital, Zhejiang University School of Medicine, Hangzhou, China; ^3^Department of Surgery, Fourth Affiliated Hospital, Zhejiang University School of Medicine, Yiwu, China; ^4^Hessian Health Technology Co., Ltd, Beijing, China; ^5^Department of Surgery, ShengZhou Hospital of Traditional Chinese Medicine, Shaoxing, China

**Keywords:** indoor daylight levels, general surgery, hospital costs, length of stay, illiteracy

## Abstract

**Background:**

Indoor daylight levels can directly affect the physical and psychological state of people. However, the effect of indoor daylight levels on the clinical recovery process of the patient remains controversial. This study was to evaluate the effect of indoor daylight levels on hospital costs and the average length of stay (LOS) of a large patient population in general surgery wards.

**Methods:**

Data were collected retrospectively and analyzed of patients in the Second Affiliated Hospital of Zhejiang University, School of Medicine between January 2015 and August 2020. We measured daylight levels in the patient rooms of general surgery and assessed their association with the total hospital costs and LOS of the patients.

**Results:**

A total of 2,998 patients were included in this study with 1,478 each assigned to two daylight level groups after matching. Overall comparison of hospital total costs and LOS among patients according to daylight levels did not show a significant difference. Subgroup analysis showed when exposed to higher intensity of indoor daylight, illiterate patients had lower total hospital costs (CNY ¥13070.0 vs. ¥15210.3, *p* = 0.018) and shorter LOS (7 vs. 10 days, *p* = 0.011) as compared to those exposed to a lower intensity.

**Conclusions:**

Indoor daylight levels were not associated with the hospital costs and LOS of patients in the wards of general surgery, except for those who were illiterate. It might be essential to design guidelines for medical staff and healthcare facilities to enhance the indoor environmental benefits of daylight for some specific populations.

## Introduction

Human health is undoubtedly one of the most important considerations for all kinds of environmental designs. Most of the research related to the impact of environmental elements on occupants has been oriented toward offices and schools rather than focusing on healthcare facilities; however, the in-patient room is a special indoor environment, requiring both extensive experimental and field research efforts to enhance and the treatment of diseases and accelerate the recovery of patients (1). As an important environmental factor, daylight levels will directly affect the physical and psychological state of people under certain conditions. It has been proven that it can elicit immediate physiological changes in body temperature, heart rate, hormones, cognition, mood, and even gene expression, which is intensively related to clinical recovery (1–5).

Previous studies have found that increased daylight exposure in wards can have some positive effects, such as accelerating the discharge of patients with depression (6) and myocardial infarction (7) and reducing the mortality of some cancers such as ovarian cancer, breast cancer, and colon cancer (8). However, others have shown that ambient daylight levels in an intensive care unit (ICU) room did not improve outcomes for critically ill patients, namely, hospital length of stay (LOS), intravenous sedative or analgesic use, and the development of ICU-acquired delirium (9–12). Thus, the effect of indoor daylight levels on the clinical recovery process of the patient remains controversial. In addition, studies on the relationship between light levels and surgery outcomes have been limited to spinal surgery (13) or cardiac surgery (14), etc. and the sample size of these studies was generally small. There has not been an independent study on patients who undergo general surgeries.

Therefore, the purpose of this research was to evaluate the effect of indoor daylight levels on hospitalization, particularly hospital costs and LOS, of a large patient population in general surgery wards. To do this, we measured daylight levels in the patient rooms of general surgery and assessed their association with the total hospital costs and LOS of the patients. Every patient was assigned to a bed upon admission based on availability, without regard to whether there was a window by the side, therefore creating a natural randomized experiment.

## Methods

### Study Population and Data Collection

The study protocol was approved by the Institutional Review Board of the Second Affiliated Hospital of Zhejiang University, School of Medicine, China. A retrospective cohort study was conducted to assess hospital outcomes between patients that were either exposed to the high light side (window) or low light side (door). Adult patients (aged 18 years and older) who had been admitted to the Department of General Surgery between January 1, 2015 and August 28, 2020 were enrolled. We collected patient data, namely, general characteristics (sex, age, and BMI at admission), clinical characteristics (treatment, diagnostic categories, and comorbidities), and demographic characteristics, namely, lifestyle factors (smoking and drinking), residential district (urban or rural), and educational levels. Total costs (Chinese Yuan, CNY ¥) and length of days during stay in hospital were collected as primary outcomes.

### Hospital Building and Patient Units

**Figure 1:**
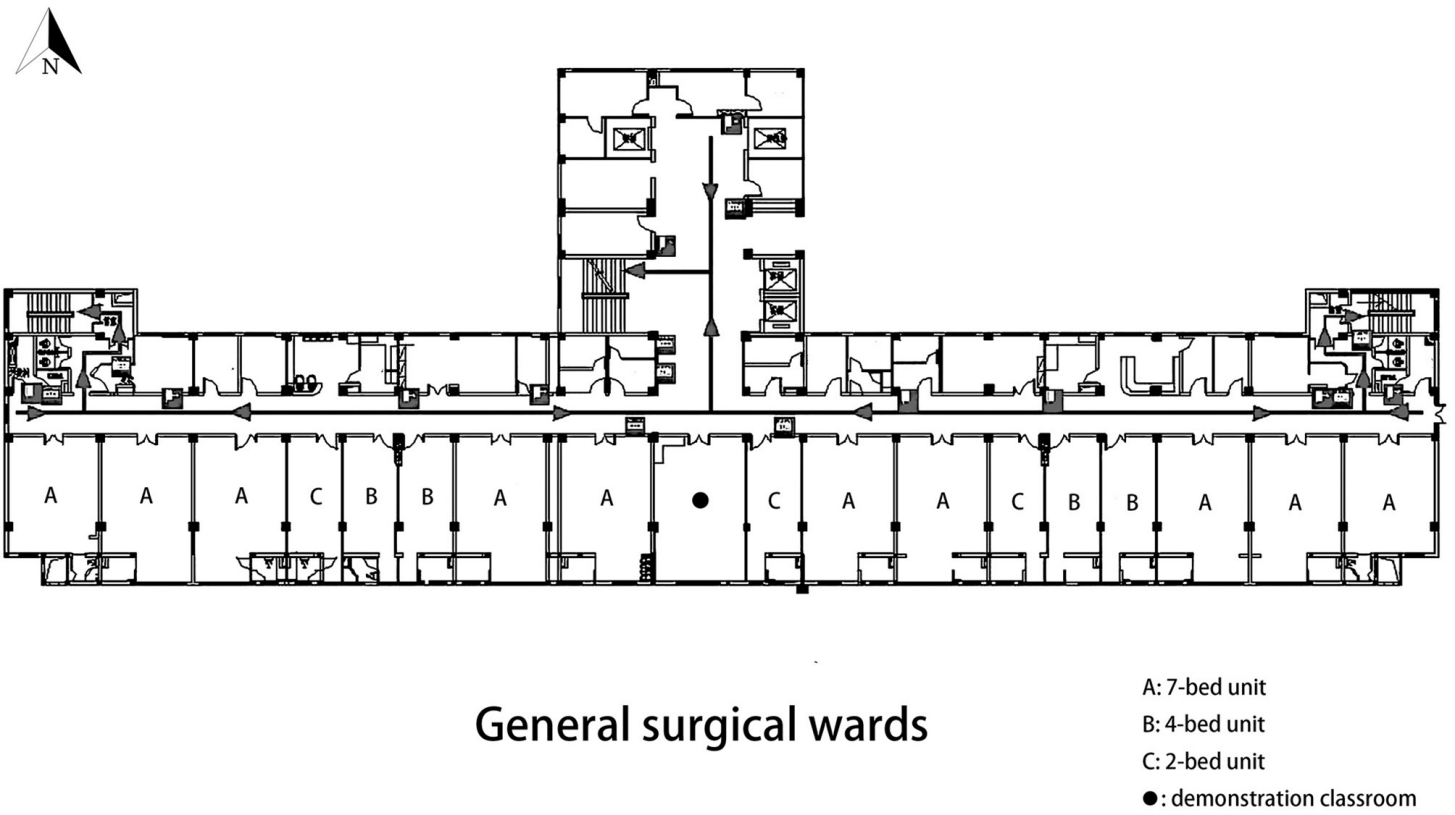
Layouts of the patient units in the general surgical wards.

**Figure 2:**
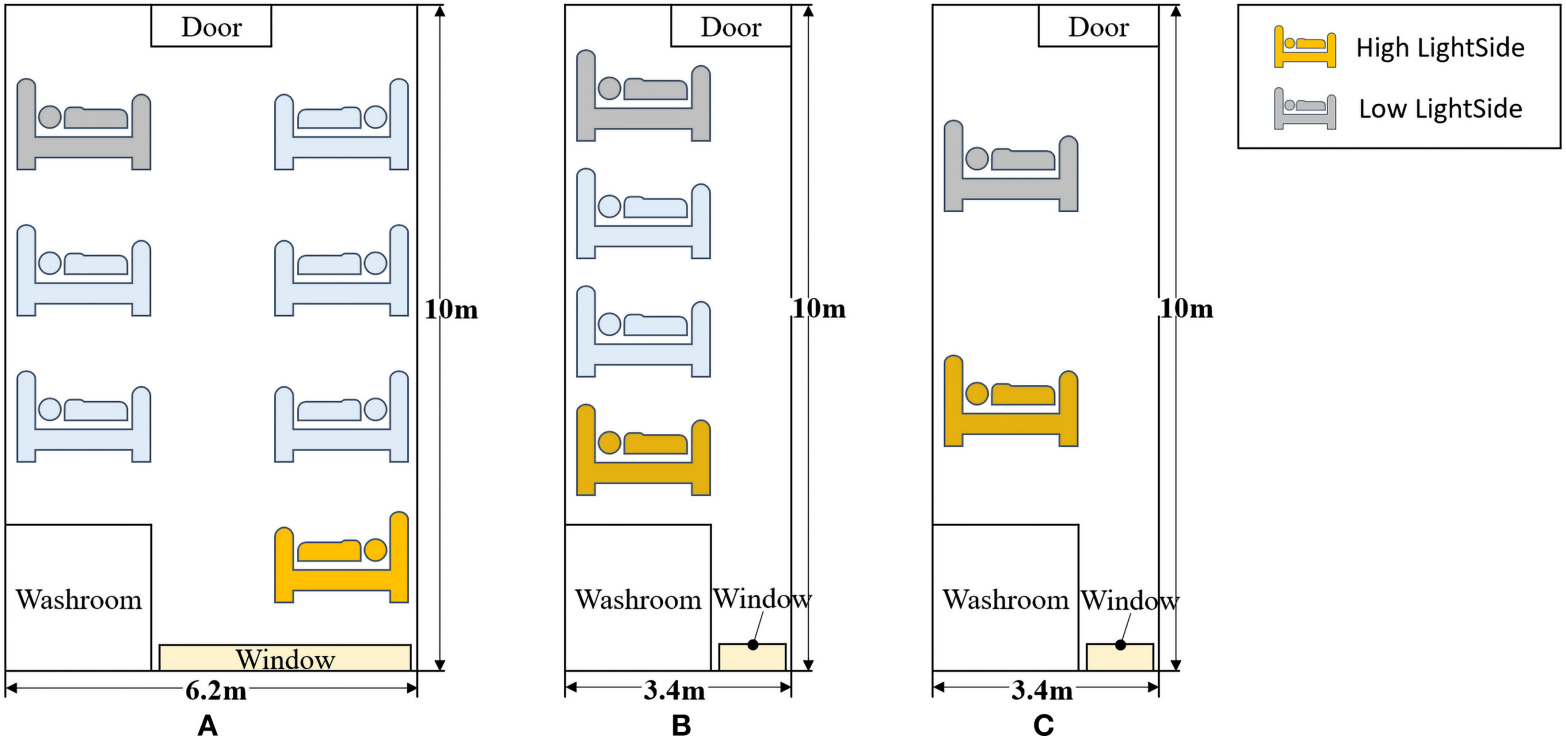
Furnished plan and arrangement of beds for each ward type. **(A–C)** In each room, the patient beds near the door are assigned to the low light side group and beds near the window are assigned to the high light side group.

Located in Hangzhou, Zhejiang, China, SANZU has a total of 3,200 beds and provides nearly 190,000 inpatient services and 150,000 surgeries every year. The hospital building included in this study has 9 floors and each floor has 17 inpatient units. As shown in Figure 1, all units are facing south to allow plenty of sunlight to enter the space. The general surgical wards are located on the 6th floor and comprise of three types of rooms: ten 7-bed rooms, four 4-bed rooms, and three 2-bed rooms. In each room, the patient beds near the door (north) are assigned to the low light side group and beds near the window (south) are assigned to the high light side group. The furnished plan and arrangement of beds for each ward type are shown in Figure 2.

### Measurement of Daylight Intensity

Daylight levels were measured using the illuminance meter (AS813 Smart Sensor; Arco Electronics Ltd., Hong Kong, China). The illuminance meter was placed on the back wall over the head of the patient and positioned toward the window of the hospital unit to measure how much light arrived at the vertical plane of the bed at the head position. In the meantime, the electric light of each room was turned off. We selected one typical clear, sunny day and one typical overcast day around autumnal equinox (September 22 in 2020) to measure levels of daylight. Measurements were taken 8 times a day every hour from 6:00 AM to 6:00 PM at each side (window and door) and at three types of patient units. Each measurement was repeated 5 times and then averaged to obtain a reliable estimate of the light intensity (lux). These reliable estimates were then averaged again across room types to obtain the overall daylight intensity estimates.

### Subgroup Definitions

Given the large sample size, we conducted subgroup analyses to assess for interaction between patients (sex, age, BMI, comorbidity, hospital characteristics, and demographics of patients) and to assess the association between daylight levels and outcomes. The subgroups consist of 10 main groups as defined in Table 1: (1) Sex, (2) Age, (3) BMI, (4) LOS, (5) Surgery, (6) Diagnosis, (7) Comorbidity, (8) Patients' Demographics, (9) District, and (10) Education level. Age was categorized into four groups according to the median and interquartile range values in all participants (<51, 51–59, 60–67, and ≥68 years). Body-mass index (BMI) was calculated as weight in kilograms divided by the square of the height in meters (kg/m^2^) and was categorized into four groups according to cut-off values indicated in The BMI criteria adopted by Chinese Adults Overweight and Obesity Prevention and Control Guidelines: underweight (<18.5 kg/m^2^), normal range (18.5–24 kg/m^2^), overweight (24–30 kg/m^2^), and obese (≥30 kg/m^2^) (15). LOS was categorized into four groups: 1–6, 7–13, 14–29, and ≥30 days). The diagnosis was categorized into six groups: benign tumor, malignant tumor, inflammation, hernia, intestinal obstruction, and others. The district was divided into two groups: rural area and urban area. Education level was categorized into five groups: illiterate (defined as never received formal education), primary school, middle school, high school, and university degree. To find out the differences between the groups, we further divided subgroups into plural subgroups. A total of 616 plural subgroups were generated for further comparisons.

**Table 1:** Basic characteristics of patients on low light and high light sides of the hospital unit.

**Variables** ^**a**^		**Light sides**		***p*-value^**c**^**
		**Low (*n* = 1,478)**	**High (*n* = 1,478)**	
1. Sex				0.574
	1 [Male]	889 (60.1) ^b^	873 (59.1)	
	2 [Female]	589 (39.9)	605 (40.9)	
2. Age				0.616
	1 [ <51 years]	341 (23.1)	358 (24.2)	
	2 [51–59 years]	358 (24.2)	361 (24.4)	
	3 [60–67 years]	365 (24.7)	376 (25.4)	
	4 [≥68 years]	414 (28.0)	383 (25.9)	
3.BMI				0.213
	1 [ <18.5]	208 (14.1)	239 (16.2)	
	2 [18.5–24]	967 (65.4)	918 (62.1)	
	3 [24–28]	255 (17.3)	263 (17.8)	
	4 [≥28]	48 (3.2)	58 (3.9)	
4. Length of Stay (LOS)				0.378
	1 [1–6 days]	753 (50.9)	753 (50.9)	
	2 [7–13 days]	386 (26.1)	405 (27.4)	
	3 [14–29 days]	261 (17.7)	261 (17.7)	
	4 [≥30 days]	78 (5.3)	59 (4.0)	
5. Surgery				0.509
	0 [No]	269 (18.2)	284 (19.2)	
	1 [Yes]	1209 (81.8)	1194 (80.8)	
6. Diagnosis				0.944
	1 [Benign tumor]	955 (64.6)	976 (66.0)	
	2 [Malignant tumor]	99 (6.7)	98 (6.6)	
	3 [Inflammation]	244 (16.5)	239 (16.2)	
	4 [Hernia]	30 (2.0)	25 (1.7)	
	5 [Intestinal obstruction]	62 (4.2)	55 (3.7)	
	6 [Others]	88 (6.0)	85 (5.8)	
7.Comorbidity				0.937
Hypertension	0 [No]	1,007 (68.1)	1,004 (67.9)	
	1 [Yes]	471 (31.9)	474 (32.1)	
Diabetes				0.268
	0 [No]	1,281 (86.7)	1,302 (88.1)	
	1 [Yes]	197 (13.3)	176 (11.9)	
8. Demographics				0.405
Smoking	0 [No]	900 (60.9)	923 (62.4)	
	1 [Yes]	578 (39.1)	555 (37.6)	
Drinking				0.676
	0 [No]	917 (62.0)	929 (62.9)	
	1 [Yes]	561 (38.0)	549 (37.1)	
9.District				**0.028**
	0 [Rural]	509 (34.4)	452 (30.6)	
	1 [Urban]	969 (65.6)	1,026 (69.4)	
10.Education level				0.086
	1 [Illiterate]	131 (8.9)	114 (7.7)	
	2 [Primary school]	509 (34.4)	455 (30.8)	
	3 [Middle school]	426 (28.8)	482 (32.6)	
	4 [High school]	219 (14.8)	234 (15.8)	
	5 [University degree]	193 (13.1)	193 (13.1)	

*^a^Variable names are presented as categorical label [name].*

*^b^Variables are listed as columnwise no. (percentage).*

*^c^p-value is determined by a chi-squared test between low and high light sides.*

*P-value less than 0.05 will be in bold*.

### Patients Matching

Variables such as patient age could potentially confound the relationship between daylights and outcomes, so we conducted a matched analysis. Patients were 1:1 matched so that one member of each pair had one patient on the high light side (window) and one patient on the low light side (door). The criteria for matching were sex, age, and admitting unit. We performed the nearest neighbor matching algorithms using the MatchIt package in R.

### Statistical Analysis

We compared descriptive characteristics and hospital outcomes between patient groups exposed to different light sides after matching. For continuous variables (total costs and length of days of hospitalization) were performed using the *t*-test (or the nonparametric Wilcoxon Rank Sum test for two groups in the case of continuous data with nonhomogenous variances). Meanwhile, nominal variables (e.g., sex and age groups) were analyzed using the chi-squared test. Statistical significance was considered at the level of *p* < 0.05 based on a two-tailed comparison. All statistical calculations were performed by R software version 4.0.2. (www.Rproject.org).

## Results

### Basic Characteristics

A total of 2,998 patients were included in this study. After matching, 1,478 patients were assigned to the low light side group, and 1,478 patients were assigned to the high light side group (Table 1). Most variables did not show a significant difference between these two groups, except the district. There were more patients from the urban area in the high light side group than in the low light side group (69.4% vs. 65.6%, *p* = 0.028).

### Daylight Intensity

**Figure 3:**
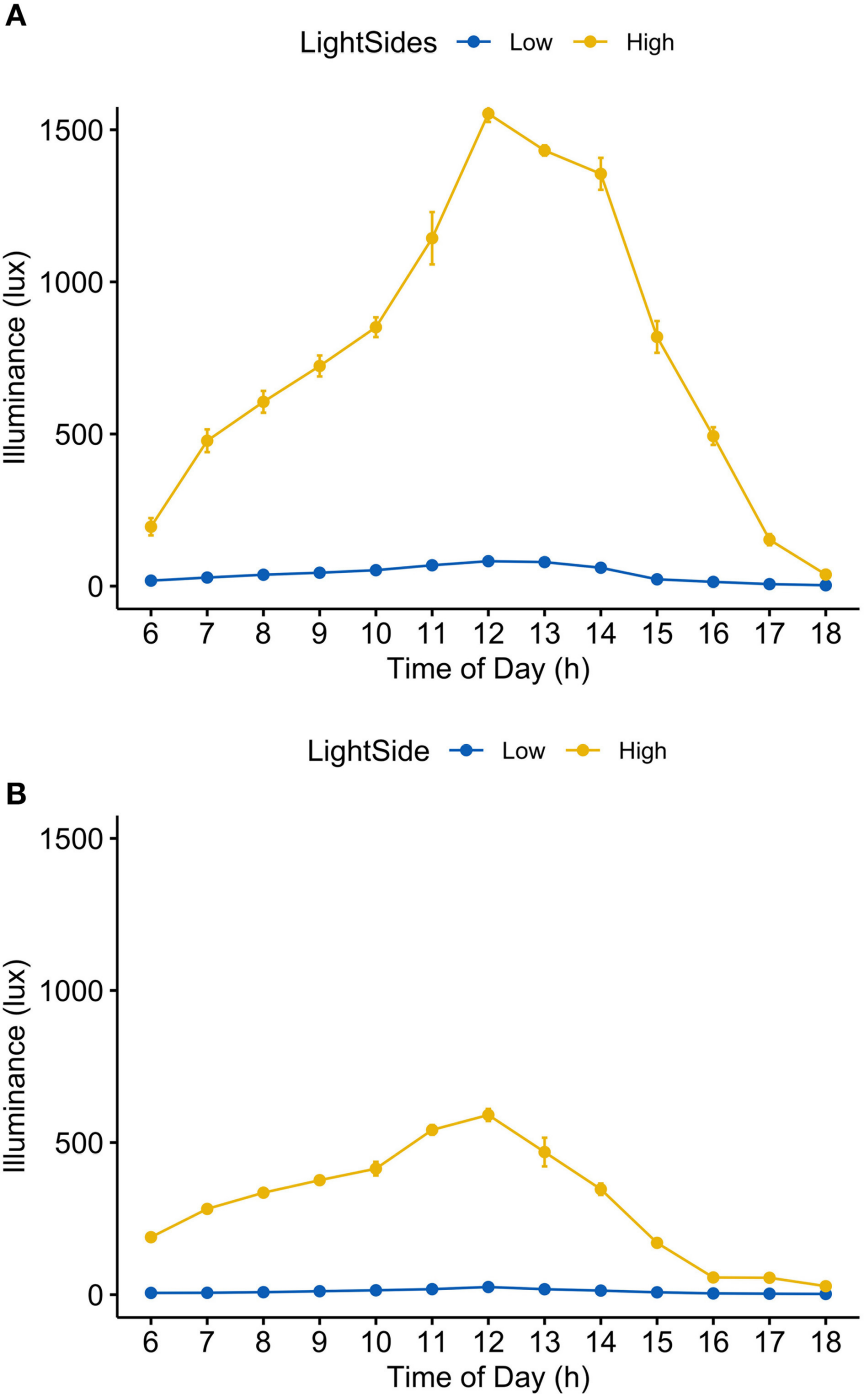
Light intensity by light sides of the hospital unit in two sky conditions. **(A)** Sunny day; **(B)** overcast day.

Average daylight intensity across daily hours in the high light group was significantly higher than that in the low light group both on a sunny day and overcast day (sunny day: low light group = 39.7 ± 28.2 lux, high light group = 756.9 ± 489.1 lux, *p* < 0.001; overcast day: low light group = 10.7 ± 7.1 lux, high light group = 296.6 ± 183.8 lux, *p* < 0.001, mean ± SD; Figures 3A,B; Supplementary Table 1).

### Hospital Costs and Length of Stay

**Figure 4:**
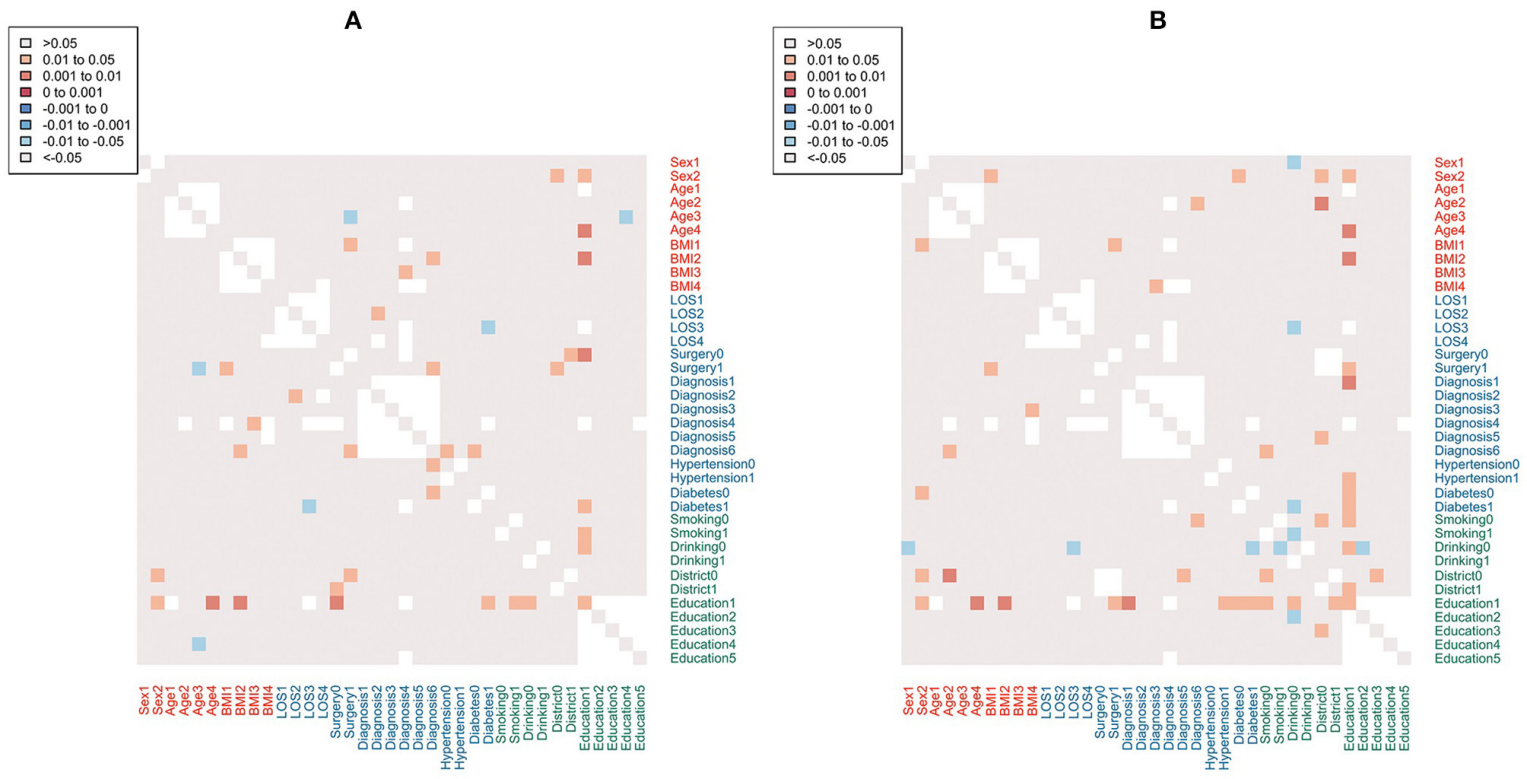
Heatmaps of *p* values for hospital total costs **(A)** and LOS **(B)** between the high light side group and the low light side group by plural subgroups. Positive coefficients (*p* > 0) indicate a direct relationship between variables (low light side > high light side); while negative coefficients (*p* < 0) indicate an inverse correlation (low light side < high light side). No *p*-values if sample size is <3. LOS, length of stay.

Overall comparison of hospital total costs among patients according to light sides did not show a significant difference (CNY ¥14182.0 vs. ¥13724.0, *p* = 0.229). By dividing the patients into subgroups according to their characteristics, we found that in most subgroups, the values of median hospital total costs in the low light side group were higher than that in the high light side group. Particularly in the illiterate subgroup (education level = 1), the difference in median hospital total costs was statistically significant (CNY ¥15210.3 vs. ¥13070.0, *p* = 0.018) (Table 2). Table 3 compares the median LOS between the low light side group and the high light side group. Overall, there was no statistically significant difference among patients in these two groups (6 vs. 6 days, *p* = 0.579). However, in the illiterate subgroup, statistically higher LOS was observed for participants in the low light side group (10 vs. 7 days, *p* = 0.011). Figures 4A,B presents the heat maps visualizing the *p*-values obtained by comparing hospital total costs and LOS between the high light side group and the low light side group in the plural subgroups. There were more significant differences in hospital total costs in the plural subgroups of the illiterate subgroup (Figure 4A). The most significant differences were found in three plural subgroups: age ≥68 years old (number of patients: 61 vs. 59, *p* = 0.001), normal range BMI (number of patients: 81 vs. 68, *p* = 0.002), and nonsurgery (number of patients: 20 vs. 26, *p* = 0.006). We also found more significant differences in LOS in the plural subgroups of the illiterate subgroup (Figure 4B). The most significant differences were shown in three plural subgroups: age ≥68 years old (number of patients: 61 vs. 59, *p* = 0.003), normal range of BMI (number of patients: 81 vs. 68, *p* = 0.009), and diagnosis of benign tumors (number of patients: 81 vs. 63, *p* = 0.005). Detailed *p*-values could be found in Supplementary Tables 2, 3.

**Table 2:** Total costs of patients by light sides of the hospital unit.

**Variables** ^**a**^		***N***	**Light sides**		***P*-value ^**d**^**
			**Low (CNY ¥) ^**b**^**	**High (CNY ¥)**	
Overall		1,478/1,478	14,182.0 (7,692.0–25,796.0)	13,724.0 (7,808.0–23,838.0)	0.229
**1. Sex**					
	1 [Male]	889/873	14,494.9 (7,809.2–24,874.0)	14,322.0 (8,115.0–25,798.0)	0.966
	2 [Female]	589/605	13,947.6 (7,440.5–27,504.0)	13,050.0 (7,607.0–21,426.0)	0.069
**2. Age**					
	1 [ <51 years]	341/358	13,247.5 (7,955.8–23,348.9)	12,672.0 (7,810.0–18,770.0)	0.272
	2 [51–59 years]	358/361	13,953.4 (7,585.0–24,415.4)	13,842.0 (7,597.0–21,240.0)	0.532
	3 [60–67 years]	365/376	13,610.0 (7,234.2–21,366.9)	14,495.0 (8,228.0–23,199.0)	0.094
	4 [≥68 years]	414/383	16,281.7 (8,501.0–36,644.3)	15,143.0 (7,691.0–35,494.0)	0.496
**3. BMI**					
	1 [ <18.5]	208/239	11,986.2 (6,204.5–19,008.2)	11,483.0 (6,531.0–18,565.0)	0.374
	2 [18.5–24]	967/918	14,269.3 (7,910.0–26,474.1)	13,749.0 (7,815.0–22,198.0)	0.229
	3 [24–28]	255/263	15,638.5 (8,749.4–26,372.1)	15,183.0 (8,977.0–30,893.0)	0.901
	4 [≥28]	48/58	18,403.5 (10,904.2–25,688.6)	17,457.0 (8,269.0–30,022.0)	0.571
**4. Length of Stay (LOS)**					
	1 [1–6 days]	753/753	9,154.2 (5,274.6–14,086.1)	9,101.0 (5,409.0–13,642.0)	0.627
	2 [7–13 days]	386/405	15,991.3 (11,506.9–24,273.6)	15,479.0 (10,966.0–22,747.0)	0.500
	3 [14–29 days]	261/261	41,760.9 (27,625.0–57,664.1)	42,646.0 (29,394.0–59,278.0)	0.991
	4 [≥30 days]	78/59	85,389.3 (66,518.6–117,553.7)	83,964.0 (64,563.0–100,000.0)	0.748
**5. Surgery**					
	0 [No]	269/284	12,153.4 (5,762.8–18,499.6)	10,468.0 (6,033.0–16,580.0)	0.360
	1 [Yes]	1,209/1,194	14,685.4 (8,361.3–28,337.9)	14,514.0 (8,345.0–28,189.0)	0.485
**6. Diagnosis**					
	1 [Benign tumor]	955/976	14,042.7 (7,372.4–23,920.0)	13,845.0 (8,012.0–23,803.0)	0.386
	2 [Malignant tumor]	99/98	13,959.3 (7,165.3–25,773.9)	10,112.0 (5,688.0–23,902.0)	0.561
	3 [Inflammation]	244/239	13,837.9 (10,139.0–25,526.3)	13,814.0 (8,535.0–22,096.0)	0.831
	4 [Hernia]	30/25	14,692.1 (11,539.6–17,006.9)	13,952.0 (12,230.0–17,129.0)	0.290
	5 [Intestinal obstruction]	62/55	19,341.6 (9,240.2–35,478.9)	16,378.0 (7,473.0–37,040.0)	0.569
	6 [Others]	88/85	19,639.4 (7,430.1–36,179.6)	12,320.0 (6,067.0–29,873.0)	0.079
**7. Comorbidity**					
Hypertension	0 [No]	1,007/1004	13,610.0 (7,283.9–25,530.2)	13,135.0 (7,556.0–21,242.0)	0.174
	1 [Yes]	471/474	15,186.2 (8,792.6–26,905.5)	15,062.0 (8,314.0–29,577.0)	0.765
**Diabetes**					
	0 [No]	1,281/1,302	14,042.7 (7,492.3–25,801.0)	13,695.0 (7,815.0–22,598.0)	0.090
	1 [Yes]	197/176	14,856.0 (7,962.4–25,781.5)	13,922.0 (7,612.0–31,176.0)	1.000
**8. Demographics**					
Smoking	0 [No]	900/923	14,101.5 (7,490.4–26,998.3)	13,764.0 (7,800.0–22,828.0)	0.098
	1 [Yes]	578/555	14,369.1 (7,974.5–23,980.2)	13,649.0 (7,944.0–25,555.0)	0.817
**Drinking**					
	0 [No]	917/929	13,837.0 (7,046.1–23,940.7)	13,571.0 (7,668.0–22,814.0)	0.625
	1 [Yes]	561/549	14,870.2 (9,332.6–28,315.1)	13,950.0 (8,164.0–25,703.0)	0.193
**9. District**					
	0 [Rural]	509/452	13,864.3 (7,313.0–25,953.7)	12,227.0 (6,537.0–20,951.0)	0.051
	1 [Urban]	969/1,026	14,344.7 (7,962.4–25,522.5)	14,180.0 (8,386.0–25,579.0)	0.714
**10. Education level**					
	1 [Illiterate]	131/114	15,210.3 (8,108.2–48,915.6)	13,070.0 (6,831.0–29,089.0)	**0.018**
	2 [Primary school]	509/455	14,269.3 (7,501.3–25,467.9)	13,814.0 (7,817.0–22,620.0)	0.548
	3 [Middle school]	426/482	14,095.7 (7,579.5–22,886.4)	12,954.0 (7,361.0–23,115.0)	0.184
	4 [High school]	219/234	14,797.1 (7,478.4–23,053.0)	15,673.0 (9,360.0–25,099.0)	0.693
	5 [University degree]	193/193	13,247.5 (9,037.5–26,890.8)	13,764.0 (8,402.0–23,448.0)	0.940

*^a^Variable names are presented as categorical label [definition].*

*^b^CNY, Chinese Yuan. CNY/USD = 0.15 (exchange rate of CNY/USD at 4 pm of 2021-07-19 Coordinated Universal Time).*

*^c^Listed as median (Q1–Q3).*

*^d^p-values are determined by the t-test or Wilcoxon Rank Sum test. p-value for overall comparison is determined by the paired t-test. P-value less than 0.05 will be in bold*.

**Table 3:** Length of stay (LOS) of patients by light sides of the hospital unit.

**Variables** ^**a**^		***N***	**Light sides**		***P*-value^**c**^**
			**Low (days)**	**High (days)**	
Overall		1,478/1,478	6 (3–13)	6 (3–12)	0.579
**1. Sex**					
	1 [Male]	889/873	6 (3–12)	6 (3–13)	0.329
	2 [Female]	589/605	7 (4–14)	7 (4–12)	0.063
**2. Age**					
	1 [ <51 years]	341/358	6 (3–11)	6 (3–11)	0.392
	2 [51–59 years]	358/361	7 (3–12)	6 (3–11)	0.121
	3 [60–67 years]	365/376	5 (2–10)	6 (3–13)	0.067
	4 [≥68 years]	414/383	7 (4–16)	8 (4–15)	0.645
**3. BMI**					
	1 [ <18.5]	208/239	5 (2–12)	5 (2–11)	0.244
	2 [18.5–24]	967/918	6 (3–13)	6 (3–12)	0.636
	3 [24–28]	255/263	7 (4–12)	7 (4–14)	0.350
	4 [≥28]	48/58	9 (6–14)	7 (4–15)	0.956
**4. Length of Stay (LOS)**					
	1 [1–6 days]	753/753	3 (2–5)	3 (2–5)	0.702
	2 [7–13 days]	386/405	9 (7–11)	9 (7–11)	0.723
	3 [14–29 days]	261/261	18 (15–21)	18 (15–22)	0.483
	4 [≥30 days]	78/59	36 (33–45)	38 (34–50)	0.220
**5. Surgery**					
	0 [No]	269/284	6 (3–11)	6 (3–9)	0.340
	1 [Yes]	1,209/1,194	6 (3–13)	7 (3–13)	0.810
**6. Diagnosis**					
	1 [Benign tumor]	955/976	5 (2–12)	5 (2–11)	0.622
	2 [Malignant tumor]	99/98	8 (6–12)	7 (5–14)	0.451
	3 [Inflammation]	244/239	8 (5–13)	7 (5–13)	0.908
	4 [Hernia]	30/25	7 (5–10)	7 (6–10)	0.423
	5 [Intestinal obstruction]	62/55	9 (6–18)	9 (6–15)	0.552
	6 [Others]	88/85	10 (7–18)	9 (6–15)	0.384
**7. Comorbidity**					
Hypertension	0 [No]	1,007/1,004	6 (3–12)	6 (3–11)	0.723
	1 [Yes]	471/474	7 (3–13)	7 (3–14)	0.635
**Diabetes**					
	0 [No]	1,281/1,302	6 (3–13)	6 (3–12)	0.280
	1 [Yes]	197/176	6 (2–11)	7 (3–14)	0.153
**8. Demographics**					
Smoking	0 [No]	900/923	7 (4–13)	7 (4–12)	0.214
	1 [Yes]	578/555	5 (2–12)	6 (2–12)	0.517
**Drinking**					
	0 [No]	917/929	6 (3–12)	7 (3–12)	0.856
	1 [Yes]	561/549	6 (3–13)	6 (3–13)	0.273
**9. District**					
	0 [Rural]	509/452	6 (3–13)	6 (3–11)	0.212
	1 [Urban]	969/1,026	6 (3–13)	7 (3–12)	0.743
**10. Education level**					
	1 [Illiterate]	131/114	10 (5–19)	7 (4–14)	**0.011**
	2 [Primary school]	509/455	6 (2–12)	7 (3–13)	0.188
	3 [Middle school]	426/482	6 (3–10)	6 (3–11)	0.212
	4 [High school]	219/234	6 (3–13)	7 (4–12)	0.857
	5 [University degree]	193/193	7 (4–12)	7 (4–13)	0.503

*^a^Variable names are presented as categorical label [definition].*

*^b^LOS is listed as median (Q1–Q3).*

*^c^p-values are determined by the t-test or Wilcoxon Rank Sum test. p-value for overall comparison is determined by the paired t-test.*

*P-value less than 0.05 will be in bold*.

## Discussion

In this study, we found no significant association between daylight exposure and the hospital costs and LOS of the patients in the wards of general surgery, regardless of their general characteristics (sex, age, and BMI at admission), clinical characteristics (taking surgery or not, diagnostic categories, and comorbidities), and demographic characteristics, namely, lifestyle factors (smoking and drinking), residential district (urban or rural), etc. However, subgroup analysis showed that patients with the lowest education level were prone to be more easily affected by daylight levels, especially when they were old-aged (≥68 years old) or within the normal BMI range (18.5–24 kg/m^2^). When exposed to higher intensity of indoor daylight, they had lower total hospital costs and shorter LOS as compared to those exposed to a lower intensity.

So far, numerous studies have been carried out to clarify the potential importance of different aspects of the environment for health and healing, such as indoor air quality and noise (16–19). As one of the most intuitive environmental factors, it is well-known that daylight may exert a significant impact on the physical and psychological well-being of patients in various direct or indirect ways (20). However, in this research, the absence of association with total hospital costs and LOS may reflect differences in daylight levels that were not physiologically sufficient for statistical significance. What is interesting is that this work also demonstrated that in contrast with patients with higher education levels, illiterate ones did get affected by indoor daylight levels. Undoubtedly, the education levels of patients are often perceived as an adequate index for their socioeconomic status, apart from their income and occupation (21). It has been proved that individuals with lower socioeconomic status tend to suffer from higher risks of psychiatric problems, namely, depression, anxiety disorders, and posttraumatic stress disorder (22–25). And lower levels of knowledge were reported to be associated with significantly higher healthcare costs, due to bad medication adherence (26). Particularly, illiterate patients would find it difficult to understand and follow the guidance from doctors and nurses, which might hinder the treatment process. Besides, according to the results of subgroup analysis, the most undereducated patients who were also old-aged (≥68 years old) and within normal BMI range (18.5–24 kg/m^2^) were more sensitive to light intensity, which was in line with a previous finding that among the elderly group, there is an inverse correlation between anxiety and obesity (27). Moreover, the lack of sample size of the subgroup might explain the statistical insignificance in underweighted (BMI <18.5 kg/m^2^) illiterate patients. Therefore, given the immobility of these characteristics, medical staff should collect information about education level, BMI, and age at admission and enhance indoor environmental benefits to accelerate the recovery process. Furthermore, the association between light levels and outcomes in this specific population also suggested that it was necessary to develop regulations of light in architectural and engineering guidelines of health facilities especially in China and a recommended standard of a minimum light intensity might be essential. According to the Illuminating Engineering Society of North America (IESNA), the recommended illuminance for the inpatient room is 300 lux (28). So far, no authoritative consensus-based standards body, namely, IESNA and International Commission on Illumination, has approved recommended levels for healthy daytime, evening, and night-time indoor light exposure (29).

Unlike our study, previous research showed a significant relationship between indoor daylight environments and an average LOS of the patient in the department of surgery (*p* <0.048) and gynecology (*p* < 0.015) in a hospital (1). Another work by Joarder et al. developed a multiple linear regression to describe the relationship of daylight illuminance and LOS of patients who underwent coronary artery bypass graft (14). Yet, the former study found no significant results in the department of internal medicine and otolaryngology. In addition, unlike these researches, our study included a much larger sample size with detailed clinical information and demographic characteristics, which may account for the different results. In general, our findings are consistent with those studies investigating the effect of indoor daylight levels on the recovery of patients in ICU. Wunsch et al. found that the presence of a window in an ICU room was not beneficial for critically ill patients with subarachnoid hemorrhage (10). Likewise, Smonig et al. pointed out that exposure to natural light did not help improve delirium burden (11). In addition, Verceles et al. indicated that room orientation with different ambient light levels did not exert a significant impact on critical care outcomes or differences in sedative/analgesic/neuroleptic use (12). However, due to limited sample size, these researches did not take into account the demographic information of patients. Moreover, it is noteworthy that patients in ICU are often in sedation and analgesia, which might make external stimuli less potent than they would be for patients in ordinary hospital wards. Even awake patients with brain injury or some other diseases may have photophobia, which might disturb the light exposure they receive.

To the best of our knowledge, this is the first study to assess the potential effect of indoor daylight levels on the recovery of patients admitted to the department of general surgery with such a large sample size. Although it was not a randomized controlled trial, patients were naturally randomly assigned to window-side vs. door-side beds in the general surgery wards, which resulted in a perfect balance of almost all baseline characteristics of patients from the two different groups. Thus, there was less chance of the results or conclusions of this study being affected by potential unmeasured confounding factors. Nonetheless, given the single-center design and observational nature of this study, there are inevitably some limitations that need to be addressed. First, due to limited access to details of hospital costs, we did not figure out how indoor daylight levels affected different kinds of costs, such as nursing costs and surgical costs. Second, this study was not able to include more individualized factors such as social and cultural differences, daylight preferences, preferred activity, etc. Third, not only is the indoor daylight intensity able to influence the physiological condition of patients but also the window view itself can generate positive physiological effects (30–32). Our study cannot rule out the fact the possibility that the window view might play a more important role in affecting the recovery of illiterate patients. At last, the unknown weightage of physical recovery with psychological recuperation and LOS being <6 days for over half the cases limits the broadly applicable claim of this study. Therefore, future investigations are desired in this direction.

To conclude, in this retrospective study, we investigated associations between indoor daylight levels with hospital costs and LOS of patients admitted to general surgery. No significant difference was found between the low light group and the high light group. However, these data do support the beneficial effects of the presence of natural light from a window on outcomes in illiterate patients. Further investigations need to be done to find out the underlying physiological and social psychological mechanisms. This study could shed some light on developing design guidelines for healthcare facilities to enhance indoor environmental benefits that will accelerate the recovery of some specific population.

## Data Availability Statement

The raw data supporting the conclusions of this article will be made available by the authors, without undue reservation.

## Ethics Statement

The studies involving human participants were reviewed and approved by Institutional Review Board of the Second Affiliated Hospital of Zhejiang University, School of Medicine, China. Written informed consent was waived in this retrospective study.

## Author Contributions

XL: conceptualization, writing of the original draft, and formal analysis. JL and AS: investigation and validation. ZY: formal analysis and visualization. NW: investigation. LZ, FY, and ZP: data curation. MZ: formal analysis. YW: writing, reviewing, editing, supervision, project administration, and funding acquisition. All authors have read and approved the manuscript.

## Funding

This work was supported by the General Program of National Natural Science Foundation of China under Grant (Grant Number: 81772562, 2017) (YW) and the Fundamental Research Funds for the Central Universities (Grant Number: 2021FZZX005-08) (XL).

## Conflict of Interest

ZY and MZ were employed by the company Hessian Health Technology Co. Ltd. The remaining authors declare that the research was conducted in the absence of any commercial or financial relationships that could be construed as a potential conflict of interest.

## Publisher's Note

All claims expressed in this article are solely those of the authors and do not necessarily represent those of their affiliated organizations, or those of the publisher, the editors and the reviewers. Any product that may be evaluated in this article, or claim that may be made by its manufacturer, is not guaranteed or endorsed by the publisher.
