# Supplementary material for: The Effect of Indoor Daylight Levels on Hospital Costs and Length of Stay of Patients Admitted to General Surgery
Source: Front Public Health. 2022 Jan 20;9:678941. doi: 10.3389/fpubh.2021.678941 (PMC8810491; doi:10.3389/fpubh.2021.678941)
Supplement: Supplementary file 1 [file Table_1.DOCX]

| Supplemental Table 1 | Light intensity by light sides of the hospital unit across daylight hours in two sky conditions. | | | | |
| --- | --- | --- | --- | --- | --- |
| **Time** | **Sunny Day** | |  | **Overcast Day** | |
|  | **Low Light Side** | **High Light Side** | | **Low Light Side** | **High Light Side** |
| 6:00 | 18 | 195.2 |  | 5.8 | 189 |
| 7:00 | 28.2 | 477.8 |  | 6.2 | 282 |
| 8:00 | 37.4 | 605.8 |  | 8.2 | 335.2 |
| 9:00 | 44 | 723.6 |  | 11.4 | 376.4 |
| 10:00 | 52.4 | 851 |  | 14.4 | 414.2 |
| 11:00 | 68.6 | 1143.6 |  | 18.2 | 541.6 |
| 12:00 | 82 | 1553 |  | 25.2 | 590.8 |
| 13:00 | 79 | 1432 |  | 18 | 468.8 |
| 14:00 | 60.4 | 1355.2 |  | 13.4 | 346.6 |
| 15:00 | 22.4 | 819.2 |  | 7.8 | 170.4 |
| 16:00 | 14 | 493.2 |  | 4.2 | 56.6 |
| 17:00 | 6.6 | 152.6 |  | 3.2 | 55.6 |
| 18:00 | 2.8 | 38 |  | 2.4 | 28 |
| Mean ± SD | 39.7 ± 28.2 | 756.9 ± 489.1 | | 10.7 ± 7.1 | 296.6 ± 183.8 |
| T-test *p* value | <0.001 |  |  | <0.001 |  |

| Supplemental Table 2 | Total costs of patients by light sides of the hospital units across 616 plural subgroups. | | | | |  |  |
| --- | --- | --- | --- | --- | --- | --- | --- |
| **Subgroups** | | **Low Light Side** | |  | **High Light Side** | | ***p*-value** |
|  |  | **N** | **median (Q1-Q3)** |  | **N** | **median (Q1-Q3)** |  |
| Sex=1 | Age1 | 170 | 12936.5 (7863.0-24091.3) |  | 180 | 12810.0 (7518.0-22122.0) | 0.898 |
|  | Age2 | 194 | 14352.8 (7763.0-24415.4) |  | 202 | 15052.0 (8320.0-22707.0) | 0.175 |
|  | Age3 | 257 | 13703.1 (7362.6-21366.9) |  | 262 | 13837.0 (8372.0-22807.0) | 0.226 |
|  | Age4 | 268 | 15999.4 (9052.2-31945.5) |  | 229 | 15692.0 (7833.0-36421.0) | 0.598 |
| Sex=2 | Age1 | 171 | 13284.6 (8221.7-21453.9) |  | 178 | 12532.0 (7870.0-17484.0) | 0.117 |
|  | Age2 | 164 | 13813.6 (7479.3-24263.7) |  | 159 | 12493.0 (6807.0-19055.0) | 0.147 |
|  | Age3 | 108 | 13329.7 (6985.1-20742.2) |  | 114 | 14799.0 (7704.0-29168.0) | 0.203 |
|  | Age4 | 146 | 17493.1 (7552.7-53249.8) |  | 154 | 13497.0 (7687.0-31012.0) | 0.155 |
| Sex=1 | BMI1 | 92 | 11719.0 (6199.2-16773.2) |  | 109 | 11818.0 (7322.0-26785.0) | 0.084 |
|  | BMI2 | 608 | 14603.0 (7911.9-25481.6) |  | 561 | 13956.0 (7833.0-22330.0) | 0.416 |
|  | BMI3 | 162 | 16096.0 (8256.2-26221.1) |  | 168 | 16321.0 (9946.0-32916.0) | 0.813 |
|  | BMI4 | 27 | 18186.6 (9065.5-25267.3) |  | 35 | 19992.0 (8303.0-29643.0) | 0.461 |
| Sex=2 | BMI1 | 116 | 12426.6 (6204.5-23107.2) |  | 130 | 10097.0 (5677.0-16759.0) | 0.200 |
|  | BMI2 | 359 | 13788.4 (7885.2-28322.8) |  | 357 | 13322.0 (7814.0-22079.0) | 0.361 |
|  | BMI3 | 93 | 15244.1 (9154.2-26461.9) |  | 95 | 14711.0 (8454.0-23170.0) | 0.881 |
|  | BMI4 | 21 | 18620.3 (13593.2-25772.5) |  | 23 | 16116.0 (10024.0-29898.0) | 0.965 |
| Sex=1 | LOS1 | 480 | 9619.0 (5586.6-14703.7) |  | 455 | 9542.0 (5758.0-14150.0) | 0.919 |
|  | LOS2 | 223 | 17702.4 (12222.9-25791.3) |  | 220 | 16437.0 (11904.0-24182.0) | 0.280 |
|  | LOS3 | 147 | 42878.7 (27202.3-58374.6) |  | 161 | 44005.0 (31907.0-59771.0) | 0.590 |
|  | LOS4 | 39 | 78086.6 (61814.4-107337.5) |  | 37 | 83964.0 (64887.0-100000.0) | 0.954 |
| Sex=2 | LOS1 | 273 | 8333.2 (4477.3-13446.8) |  | 298 | 8521.0 (4689.0-12994.0) | 0.425 |
|  | LOS2 | 163 | 14980.1 (11216.4-20346.5) |  | 185 | 14821.0 (10225.0-20110.0) | 0.693 |
|  | LOS3 | 114 | 39999.7 (28147.4-55754.6) |  | 100 | 38949.0 (27965.0-58728.0) | 0.918 |
|  | LOS4 | 39 | 90737.8 (71629.9-121204.2) |  | 22 | 83859.0 (61907.0-100000.0) | 0.777 |
| Sex=1 | Surgery0 | 182 | 12103.3 (6081.2-18162.9) |  | 185 | 10756.0 (6927.0-16695.0) | 0.298 |
|  | Surgery1 | 707 | 14934.4 (8511.7-27630.5) |  | 688 | 15338.0 (8673.0-30893.0) | 0.723 |
| Sex=2 | Surgery0 | 87 | 12211.3 (4814.8-19853.3) |  | 99 | 9641.0 (5205.0-16223.0) | 0.130 |
|  | Surgery1 | 502 | 14073.2 (7788.7-30076.3) |  | 506 | 13583.0 (8024.0-24029.0) | 0.166 |
| Sex=1 | Diagnosis1 | 624 | 14185.1 (7751.8-22190.2) |  | 623 | 14567.0 (8140.0-26100.0) | 0.315 |
|  | Diagnosis2 | 40 | 14100.6 (6616.9-23614.8) |  | 38 | 11627.0 (5753.0-24472.0) | 0.345 |
|  | Diagnosis3 | 117 | 14844.5 (10102.9-27057.7) |  | 107 | 14044.0 (9427.0-23200.0) | 0.196 |
|  | Diagnosis4 | 20 | 14692.1 (12427.9-16245.8) |  | 20 | 13801.0 (12096.0-16398.0) | 0.957 |
|  | Diagnosis5 | 31 | 15658.5 (7110.4-28481.1) |  | 27 | 14447.0 (5836.0-28554.0) | 0.560 |
|  | Diagnosis6 | 57 | 18476.9 (7082.6-36105.7) |  | 58 | 15399.0 (6642.0-30729.0) | 0.152 |
| Sex=2 | Diagnosis1 | 331 | 13783.8 (6949.3-31039.3) |  | 353 | 13196.0 (7682.0-20868.0) | 0.441 |
|  | Diagnosis2 | 59 | 12046.5 (7705.0-26836.3) |  | 60 | 9505.0 (5902.0-19390.0) | 0.564 |
|  | Diagnosis3 | 127 | 13454.9 (10293.8-18916.7) |  | 132 | 13677.0 (8312.0-20878.0) | 0.542 |
|  | Diagnosis4 | 10 | 15462.7 (11414.2-23701.3) |  | 5 | 19212.0 (12230.0-49538.0) | 0.288 |
|  | Diagnosis5 | 31 | 25423.3 (10263.1-41685.7) |  | 28 | 18995.0 (11381.0-39488.0) | 0.834 |
|  | Diagnosis6 | 31 | 21002.9 (12567.4-34697.5) |  | 27 | 8733.0 (5162.0-20660.0) | 0.283 |
| Sex=1 | Hypertension0 | 584 | 13951.5 (7361.4-24344.3) |  | 563 | 13828.3 (8042.5-24016.9) | 0.566 |
|  | Hypertension1 | 305 | 15145.3 (9218.2-25066.7) |  | 310 | 16012.6 (8248.1-27641.3) | 0.410 |
| Sex=2 | Hypertension0 | 423 | 13471.3 (7062.1-26231.2) |  | 441 | 12643.1 (7377.0-19205.3) | 0.160 |
|  | Hypertension1 | 166 | 15431.9 (8643.5-31040.0) |  | 164 | 14524.6 (8397.8-30780.4) | 0.262 |
| Sex=1 | Diabetes0 | 752 | 14252.4 (7804.6-24050.2) |  | 756 | 14307.9 (8252.2-24438.6) | 0.718 |
|  | Diabetes1 | 137 | 14856.0 (7906.2-25781.5) |  | 117 | 14446.6 (7211.2-29961.2) | 0.890 |
| Sex=2 | Diabetes0 | 529 | 13788.4 (7234.2-27504.0) |  | 546 | 13035.4 (7529.1-20756.3) | 0.106 |
|  | Diabetes1 | 60 | 14788.1 (9202.2-24283.1) |  | 59 | 13227.8 (8325.6-34015.9) | 0.580 |
| Sex=1 | Smoking0 | 320 | 14535.0 (7557.7-26035.8) |  | 322 | 15397.6 (8456.9-26121.2) | 0.798 |
|  | Smoking1 | 569 | 14371.9 (7962.4-24007.1) |  | 551 | 13649.3 (7969.3-25554.9) | 0.801 |
| Sex=2 | Smoking0 | 580 | 13959.8 (7430.1-27510.3) |  | 601 | 13049.5 (7606.7-21425.8) | 0.070 |
|  | Smoking1 | 9 | 11521.4 (8030.3-19764.0) |  | 4 | 10749.6 (6446.1-24132.5) | 0.820 |
| Sex=1 | Drinking0 | 368 | 13841.3 (6822.8-21299.5) |  | 371 | 14997.2 (8086.2-27533.7) | 0.199 |
|  | Drinking1 | 521 | 14844.5 (8900.7-27609.7) |  | 502 | 13926.2 (8132.3-25390.9) | 0.394 |
| Sex=2 | Drinking0 | 549 | 13835.5 (7164.5-26978.5) |  | 558 | 12939.6 (7529.2-20756.3) | 0.152 |
|  | Drinking1 | 40 | 15574.9 (11516.6-41342.8) |  | 47 | 15158.3 (9195.5-30571.1) | 0.164 |
| Sex=1 | District0 | 305 | 13330.9 (6980.0-24441.4) |  | 256 | 12704 .0(6628.0-22381.0) | 0.434 |
|  | District1 | 584 | 14766.2 (8500.2-24891.1) |  | 617 | 15112.0 (8879.0-27009.0) | 0.597 |
| Sex=2 | District0 | 204 | 14202.2 (7499.4-30021.5) |  | 196 | 12038.0 (6484.0-19636.0) | 0.029 |
|  | District1 | 385 | 13788.4 (7399.0-26461.9) |  | 409 | 13322.0 (7804.0-21993.0) | 0.321 |
| Sex=1 | Education1 | 41 | 14827.2 (8126.2-51336.8) |  | 36 | 13950.0 (6944.0-23049.0) | 0.228 |
|  | Education2 | 319 | 14394.4 (7867.9-21975.5) |  | 254 | 13582.0 (8037.0-22797.0) | 0.454 |
|  | Education3 | 278 | 14766.2 (7814.7-25735.2) |  | 314 | 14504.0 (7541.0-26057.0) | 0.409 |
|  | Education4 | 135 | 14866.9 (7356.5-21742.0) |  | 148 | 16548.0 (10496.0-28546.0) | 0.122 |
|  | Education5 | 116 | 13232.4 (9281.0-25800.3) |  | 121 | 13956.0 (9122.0-25436.0) | 0.910 |
| Sex=2 | Education1 | 90 | 15227.2 (7821.6-45559.4) |  | 78 | 12094.0 (6831.0-29387.0) | 0.043 |
|  | Education2 | 190 | 13985.3 (7146.9-29431.4) |  | 201 | 14497.0 (7680.0-22399.0) | 0.985 |
|  | Education3 | 148 | 13565.3 (7108.0-19892.8) |  | 168 | 12422.0 (6029.0-18195.0) | 0.266 |
|  | Education4 | 84 | 14652.0 (9916.5-25772.9) |  | 86 | 13904.0 (8392.0-20417.0) | 0.239 |
|  | Education5 | 77 | 13247.5 (7955.8-29994.1) |  | 72 | 13296.0 (8160.0-19884.0) | 0.970 |
| Age=1 | BMI1 | 48 | 10603.7 (6383.1-14978.9) |  | 67 | 10749.0 (7567.0-18345.0) | 0.689 |
|  | BMI2 | 207 | 12836.0 (7990.2-22767.2) |  | 223 | 12812.0 (7919.0-18291.0) | 0.278 |
|  | BMI3 | 72 | 15764.5 (10225.0-25906.7) |  | 51 | 13510.0 (9480.0-24689.0) | 0.795 |
|  | BMI4 | 14 | 12943.4 (9270.2-18879.5) |  | 17 | 13590.0 (5745.0-16818.0) | 0.847 |
| Age=2 | BMI1 | 55 | 12973.1 (6089.5-17718.6) |  | 53 | 9448.0 (5421.0-15400.0) | 0.187 |
|  | BMI2 | 221 | 14462.9 (8057.3-25911.8) |  | 214 | 13537.0 (7524.0-19334.0) | 0.066 |
|  | BMI3 | 62 | 12803.7 (6922.9-23208.5) |  | 76 | 16127.0 (8634.0-24902.0) | 0.728 |
|  | BMI4 | 20 | 18532.8 (10904.2-25098.6) |  | 18 | 19954.0 (13099.0-42648.0) | 0.817 |
| Age=3 | BMI1 | 41 | 9665.5 (5998.1-19108.2) |  | 59 | 11526.0 (6790.0-16580.0) | 0.467 |
|  | BMI2 | 259 | 13303.9 (7313.9-20961.3) |  | 225 | 14431.0 (8231.0-21870.0) | 0.354 |
|  | BMI3 | 60 | 14650.8 (9697.4-19700.8) |  | 75 | 15408.0 (9859.0-33263.0) | 0.334 |
|  | BMI4 | 5 | 38985.1 (19177.3-62106.0) |  | 17 | 27635.0 (11841.0-37541.0) | 0.760 |
| Age=4 | BMI1 | 64 | 14009.9 (6737.9-21967.2) |  | 60 | 12138.0 (5648.0-27709.0) | 0.422 |
|  | BMI2 | 280 | 16066.4 (8881.7-39229.7) |  | 256 | 15128.0 (7801.0-35501.0) | 0.720 |
|  | BMI3 | 61 | 21165.1 (13845.6-39497.3) |  | 61 | 16279.0 (8973.0-37428.0) | 0.747 |
|  | BMI4 | 9 | 20336.4 (13733.8-25660.6) |  | 6 | 16529.0 (9026.0-28029.0) | 0.748 |
| Age=1 | LOS1 | 180 | 10020.4 (4862.9-13727.6) |  | 198 | 9976.0 (5887.8-13354.7) | 0.903 |
|  | LOS2 | 99 | 14844.5 (11077.1-25240.1) |  | 101 | 14558.9 (10998-20343.1) | 0.538 |
|  | LOS3 | 47 | 34990.6 (26625.4-47449.4) |  | 47 | 39018.7 (29010.9-55885) | 0.952 |
|  | LOS4 | 15 | 70521.8 (54729.5-96981.9) |  | 12 | 72082.7 (47931.1-78334.5) | 0.392 |
| Age=2 | LOS1 | 176 | 8174.6 (5423.2-13731.6) |  | 201 | 8881.4 (5579.7-13912.0) | 0.297 |
|  | LOS2 | 103 | 15570.7 (11467.3-23160.5) |  | 98 | 16391.1 (13048.1-22710.3) | 0.611 |
|  | LOS3 | 59 | 42185.1 (25383.6-60091.6) |  | 52 | 42019.5 (30139.1-56943.6) | 0.539 |
|  | LOS4 | 20 | 84348.6 (58637.7-123455.5) |  | 10 | 82758.3 (61599.3-145448.5) | 0.558 |
| Age=3 | LOS1 | 212 | 9067.0 (5219.6-14374.3) |  | 198 | 10042.4 (5943.5-14519.2) | 0.234 |
|  | LOS2 | 86 | 16847.7 (11473.0-23975.6) |  | 90 | 15639.0 (10201.1-22382.3) | 0.444 |
|  | LOS3 | 54 | 39462.7 (27567.3-54437.7) |  | 70 | 44920.7 (30642.5-56625.6) | 0.964 |
|  | LOS4 | 13 | 71252.1 (67034.5-104863.3) |  | 18 | 81976.3 (75112.8-117689.2) | 0.299 |
| Age=4 | LOS1 | 185 | 8910.6 (5420.9-14344.7) |  | 156 | 7533.3 (4763.9-13113.2) | 0.121 |
|  | LOS2 | 98 | 17496.8 (12797.4-24474.9) |  | 116 | 15315.4 (10329.8-24077) | 0.557 |
|  | LOS3 | 101 | 43948.3 (30818.6-59036.1) |  | 92 | 46666.9 (29927.6-66058.1) | 0.588 |
|  | LOS4 | 30 | 92204.7 (75444.7-120517.5) |  | 19 | 95752.0 (75002.0-133668.0) | 0.785 |
| Age=1 | Surgery0 | 56 | 7092.7 (2896.1-17955.3) |  | 58 | 10801.0 (5410.0-16195.0) | 0.452 |
|  | Surgery1 | 285 | 13471.3 (9509.9-24441.4) |  | 300 | 12956.0 (8455.0-20386.0) | 0.381 |
| Age=2 | Surgery0 | 56 | 11752.9 (6325.0-15753.9) |  | 65 | 10998.0 (6139.0-17118.0) | 0.996 |
|  | Surgery1 | 302 | 14447.9 (7764.2-27513.2) |  | 296 | 14077.0 (7909.0-22637.0) | 0.571 |
| Age=3 | Surgery0 | 61 | 10308.3 (6211.4-16045.6) |  | 75 | 10633.0 (6772.0-16455.0) | 0.819 |
|  | Surgery1 | 304 | 14388.0 (7355.3-22930.7) |  | 301 | 15158.0 (8620.0-30147.0) | 0.047 |
| Age=4 | Surgery0 | 96 | 14283.8 (6544.1-20927.8) |  | 86 | 9514.0 (6056.0-17168.0) | 0.051 |
|  | Surgery1 | 318 | 16945.5 (9525.8-42801.0) |  | 297 | 16616.0 (8401.0-41146.0) | 0.761 |
| Age=1 | Diagnosis1 | 165 | 13471.3 (8741.8-20715.8) |  | 201 | 13304.0 (8453.0-19589.0) | 0.288 |
|  | Diagnosis2 | 34 | 10697.4 (4955.9-26457.2) |  | 24 | 8648.0 (4403.0-15772.0) | 0.158 |
|  | Diagnosis3 | 100 | 12369.7 (9512.4-17702.9) |  | 77 | 12455.0 (8057.0-14821.0) | 0.899 |
|  | Diagnosis4 | 5 | 10112.9 (5351.9-10503.3) |  | 6 | 11915.0 (9954.0-14906.0) | 0.216 |
|  | Diagnosis5 | 14 | 24527.5 (9133.9-33319.8) |  | 17 | 18530.0 (5312.0-39019.0) | 0.492 |
|  | Diagnosis6 | 23 | 21199.8 (6971.5-35570.8) |  | 33 | 11650.0 (8019.0-20135.0) | 0.172 |
| Age=2 | Diagnosis1 | 246 | 13691.2 (7409.4-19767.5) |  | 262 | 13689.0 (7800.0-19492.0) | 0.563 |
|  | Diagnosis2 | 27 | 12380.0 (6335.8-22628.7) |  | 24 | 10741.0 (7463.0-15789.0) | 0.746 |
|  | Diagnosis3 | 44 | 14135.1 (11558.3-25676.2) |  | 45 | 14294.0 (7398.0-24748.0) | 0.705 |
|  | Diagnosis4 | 7 | 14712.9 (9755.5-17398.8) |  | 2 | 16733.0 (16535.0-16931.0) | NA |
|  | Diagnosis5 | 18 | 21517.9 (7534.9-42109.9) |  | 12 | 16522.0 (6885.0-22142.0) | 0.115 |
|  | Diagnosis6 | 16 | 42974.5 (10290.2-64887.3) |  | 16 | 16869.0 (5089.0-25770.0) | 0.063 |
| Age=3 | Diagnosis1 | 276 | 13317.4 (6881.9-20163.3) |  | 277 | 13322.0 (8219.0-22332.0) | 0.125 |
|  | Diagnosis2 | 16 | 14740.6 (8495.2-26496.2) |  | 20 | 16722.0 (9931.0-34370.0) | 0.898 |
|  | Diagnosis3 | 38 | 13834.0 (7788.2-21353.3) |  | 49 | 14887.0 (7536.0-22399.0) | 0.908 |
|  | Diagnosis4 | 7 | 15388.5 (13479.3-21957.1) |  | 5 | 15257.0 (13571.0-16579.0) | 0.503 |
|  | Diagnosis5 | 7 | 13703.1 (10515.7-41663.4) |  | 10 | 17093.0 (13561.0-32714.0) | 0.876 |
|  | Diagnosis6 | 21 | 17309.7 (6254.8-24007.1) |  | 15 | 19406.0 (4544.0-34307.0) | 0.849 |
| Age=4 | Diagnosis1 | 268 | 16205.6 (7465.8-42737.9) |  | 236 | 16011.0 (7845.0-37639.0) | 0.312 |
|  | Diagnosis2 | 22 | 16734.1 (9836.2-22390.2) |  | 30 | 7768.0 (5261.0-17671.0) | 0.532 |
|  | Diagnosis3 | 62 | 17035.0 (11900.3-34896.7) |  | 68 | 15344.0 (8825.0-27008.0) | 0.700 |
|  | Diagnosis4 | 11 | 14700.7 (14326.9-16436.7) |  | 12 | 13801.0 (11895.0-23813.0) | 0.413 |
|  | Diagnosis5 | 23 | 18974.9 (10430.7-26307.7) |  | 16 | 13266.0 (7836.0-26932.0) | 0.993 |
|  | Diagnosis6 | 28 | 19531.1 (10877.8-36356.8) |  | 21 | 10680.0 (6468.0-53424.0) | 0.610 |
| Age=1 | Hypertension0 | 314 | 13266.1 (8140.3-21854.1) |  | 332 | 12455.3 (7590.9-18269.1) | 0.177 |
|  | Hypertension1 | 27 | 12280.8 (7512.4-27280.9) |  | 26 | 15863.6 (12019.4-25855.2) | 0.416 |
| Age=2 | Hypertension0 | 257 | 13565.8 (7118.0-25772.5) |  | 268 | 13349.5 (7392.7-21464.7) | 0.218 |
|  | Hypertension1 | 101 | 14501.0 (9760.5-21496.5) |  | 93 | 14551.8 (8500.8-19962.8) | 0.498 |
| Age=3 | Hypertension0 | 232 | 12551.1 (6759.3-20786.3) |  | 225 | 14495.2 (8547.3-22238.1) | 0.377 |
|  | Hypertension1 | 133 | 14494.9 (8372.5-21366.9) |  | 151 | 14431.0 (7834.8-28131.9) | 0.112 |
| Age=4 | Hypertension0 | 204 | 16030.9 (7471.5-39229.7) |  | 179 | 14109.3 (7275.3-27447.7) | 0.573 |
|  | Hypertension1 | 210 | 16318.6 (10496.8-34313.8) |  | 204 | 15541.7 (8346.5-39148.8) | 0.629 |
| Age=1 | Diabetes0 | 328 | 12860.1 (7782.7-21639.7) |  | 350 | 12672.3 (7869.6-18487.2) | 0.264 |
|  | Diabetes1 | 13 | 18539.9 (14539.4-25781.5) |  | 8 | 13403.4 (4426.2-35897.9) | 0.815 |
| Age=2 | Diabetes0 | 307 | 13990.8 (7693.2-25817.3) |  | 307 | 13848.5 (7372.2-20893) | 0.312 |
|  | Diabetes1 | 51 | 13716.5 (7568.1-20967.1) |  | 54 | 13142.1 (8658.8-21078.2) | 0.740 |
| Age=3 | Diabetes0 | 308 | 13612.6 (6975.4-21485.7) |  | 331 | 14595.4 (8583.7-22800.3) | 0.190 |
|  | Diabetes1 | 57 | 13582.9 (7906.2-20486.4) |  | 45 | 11325.5 (5667.1-35203.3) | 0.882 |
| Age=4 | Diabetes0 | 338 | 16205.6 (8104.0-36644.3) |  | 314 | 15020.1 (7686.7-33165.8) | 0.306 |
|  | Diabetes1 | 76 | 16849.1 (12043.8-35537.3) |  | 69 | 15898.6 (7833.0-49537.8) | 0.414 |
| Age=1 | Smoking0 | 250 | 13266.1 (7566.3-23754.3) |  | 266 | 12756.5 (8081.0-18145.2) | 0.346 |
|  | Smoking1 | 91 | 13007.4 (8898.6-21414.5) |  | 92 | 12529.7 (7077.2-20847.8) | 0.578 |
| Age=2 | Smoking0 | 228 | 13786.1 (7479.3-24039.1) |  | 218 | 12973.0 (6786.9-19427.1) | 0.196 |
|  | Smoking1 | 130 | 14744.4 (8022.5-24415.4) |  | 143 | 15026.2 (9210.0-23702.8) | 0.769 |
| Age=3 | Smoking0 | 178 | 13469.1 (6614.8-20101.6) |  | 191 | 15257.2 (8161.3-24279.9) | 0.104 |
|  | Smoking1 | 187 | 14371.9 (7713.6-21513.4) |  | 185 | 12765.0 (8369.7-22332.0) | 0.392 |
| Age=4 | Smoking0 | 244 | 17043.0 (9714.2-39690.2) |  | 248 | 15442.1 (8294.5-35354.4) | 0.277 |
|  | Smoking1 | 170 | 15245.7 (7905.2-30872.5) |  | 135 | 14997.2 (6249.5-37111.1) | 0.751 |
| Age=1 | Drinking0 | 242 | 12748.3 (7371.3-22987.4) |  | 266 | 12577.5 (8024.5-18059.8) | 0.205 |
|  | Drinking1 | 99 | 13906.2 (9304.0-22669.8) |  | 92 | 13126.3 (7407.0-22122.4) | 0.925 |
| Age=2 | Drinking0 | 230 | 13746.7 (7368.3-22855.5) |  | 222 | 12792.4 (6874.7-19907.0) | 0.234 |
|  | Drinking1 | 128 | 15060.8 (9133.1-26397.8) |  | 139 | 15053.4 (9362.3-23474.8) | 0.133 |
| Age=3 | Drinking0 | 196 | 12415.7 (6046.7-19927.3) |  | 192 | 14799.4 (7330.4-27567.7) | 0.192 |
|  | Drinking1 | 169 | 14827.2 (8631.0-22949.2) |  | 184 | 13003.8 (8666.3-22284.6) | 0.297 |
| Age=4 | Drinking0 | 249 | 15960.3 (7254.8-32008.7) |  | 249 | 15230.4 (8141.7-35533.6) | 0.846 |
|  | Drinking1 | 165 | 17141.6 (10563.3-42223.2) |  | 134 | 14777.3 (7215.6-34819.5) | 0.181 |
| Age=1 | District0 | 100 | 11953.7 (6558.3-23419.6) |  | 120 | 12451.0 (6855.0-19635.0) | 0.758 |
|  | District1 | 241 | 13455.4 (9137.5-21551.9) |  | 238 | 12762.0 (8415.0-18145.0) | 0.185 |
| Age=2 | District0 | 119 | 13990.8 (8072.0-30667.0) |  | 127 | 12310.0 (6249.0-18776.0) | 0.067 |
|  | District1 | 239 | 13838.9 (7419.8-22168.3) |  | 234 | 14396.0 (8077.0-21893.0) | 0.911 |
| Age=3 | District0 | 157 | 11095.9 (7003.3-19858.6) |  | 110 | 10930.0 (6329.0-20058.0) | 0.612 |
|  | District1 | 208 | 14486.0 (7647.2-21928.7) |  | 266 | 15264.0 (9640.0-27242.0) | 0.233 |
| Age=4 | District0 | 133 | 17729.8 (7501.3-39193.4) |  | 95 | 14566.0 (7378.0-37340.0) | 0.522 |
|  | District1 | 281 | 15782.4 (9517.8-34932.5) |  | 288 | 15269.0 (7826.0-35354.0) | 0.611 |
| Age=1 | Education1 | 2 | 30145.9 (20568.2-39723.6) |  | 4 | 21966.7 (8977.6-48644.0) | NA |
|  | Education2 | 40 | 11391.3 (6016.4-26765.2) |  | 49 | 13678.1 (6963.5-19562.4) | 0.302 |
|  | Education3 | 118 | 13565.3 (8677.8-20380.2) |  | 139 | 12422.1 (7223.6-17724.9) | 0.756 |
|  | Education4 | 60 | 15315.3 (10455.5-27534.3) |  | 75 | 13063.4 (8803.2-20533.2) | 0.279 |
|  | Education5 | 121 | 12069.5 (7809.2-24158.6) |  | 91 | 13044.9 (8370.5-21940.0) | 0.927 |
| Age=2 | Education1 | 23 | 13984.4 (6637.3-26837.2) |  | 17 | 10852.1 (6139.3-18479.7) | 0.159 |
|  | Education2 | 100 | 14769.4 (8226.5-23240.6) |  | 97 | 13949.6 (8225.9-19205.3) | 0.501 |
|  | Education3 | 127 | 11715.6 (7062.1-22072.0) |  | 137 | 12114.6 (6410.0-22746.9) | 0.209 |
|  | Education4 | 78 | 14024.0 (7471.9-25441.8) |  | 77 | 16690.0 (10083.3-21650.0) | 0.796 |
|  | Education5 | 30 | 14425.2 (9558.3-25548.6) |  | 33 | 13227.8 (9276.9-18425.9) | 0.243 |
| Age=3 | Education1 | 45 | 11376.8 (6795.5-30009.5) |  | 34 | 13495.8 (6750.0-28217.0) | 0.631 |
|  | Education2 | 172 | 13364.0 (7350.6-20706.5) |  | 159 | 12986.3 (8236.5-21735.9) | 0.249 |
|  | Education3 | 98 | 15318.6 (8519.8-22302.5) |  | 117 | 15158.3 (8681.7-28030.2) | 0.479 |
|  | Education4 | 36 | 11601.8 (5438.3-16202.2) |  | 39 | 16193.0 (8733.2-29320.0) | 0.027 |
|  | Education5 | 14 | 16460.3 (10698.6-55361.1) |  | 27 | 17634.2 (8360.6-24576.9) | 0.803 |
| Age=4 | Education1 | 61 | 32008.7 (12144.1-59857.6) |  | 59 | 13216.6 (7375.2-30085.3) | 0.001 |
|  | Education2 | 197 | 15152.4 (7501.3-30732.6) |  | 150 | 15113.8 (7508.5-37349.0) | 0.313 |
|  | Education3 | 83 | 17658.1 (6994.6-40057.3) |  | 89 | 15003.6 (7527-30708.3) | 0.223 |
|  | Education4 | 45 | 16726.4 (9549.7-22260.6) |  | 43 | 18000.1 (10660.7-35282.9) | 0.300 |
|  | Education5 | 28 | 17961.9 (12011.8-33588.3) |  | 42 | 15835.9 (9179.9-35089.6) | 0.835 |
| BMI=1 | LOS1 | 124 | 7152.8 (4863.6-11582.6) |  | 143 | 8380.6 (5146.6-12014.4) | 0.261 |
|  | LOS2 | 38 | 16090.2 (13356.0-20905.1) |  | 49 | 14608.4 (9640.5-18599.6) | 0.170 |
|  | LOS3 | 30 | 36752.2 (22141.0-47717.0) |  | 32 | 38638.9 (32998.6-50796.2) | 0.698 |
|  | LOS4 | 16 | 75673.7 (59619.3-114035.7) |  | 15 | 78364.9 (53683.9-104559.3) | 0.377 |
| BMI=2 | LOS1 | 501 | 9769.1 (5351.9-14517.9) |  | 476 | 9440.7 (5357.7-13942.3) | 0.723 |
|  | LOS2 | 240 | 15347.3 (10976.5-25485.4) |  | 253 | 15314.0 (10633.5-22841.5) | 0.877 |
|  | LOS3 | 174 | 42531.9 (29027.2-59072.9) |  | 156 | 46106.6 (28814.8-60741.2) | 0.598 |
|  | LOS4 | 52 | 90201.1 (68748.8-115285.5) |  | 33 | 80087.4 (64886.9-126637.3) | 0.930 |
| BMI=3 | LOS1 | 112 | 9377.4 (5325.4-14284.4) |  | 111 | 9541.8 (6302.0-13546.2) | 0.765 |
|  | LOS2 | 91 | 17972.4 (13509.5-24943.4) |  | 86 | 16580.2 (13147.8-24120.2) | 0.677 |
|  | LOS3 | 44 | 44939.6 (30778.3-55553.2) |  | 59 | 44005.4 (33372.4-55126) | 0.300 |
|  | LOS4 | 8 | 74338.8 (61351.8-92388.6) |  | 7 | 85571.5 (77284.9-133445.3) | 0.631 |
| BMI=4 | LOS1 | 16 | 9310.0 (6759.7-14076.6) |  | 23 | 8152.9 (5630.7-17457.1) | 0.344 |
|  | LOS2 | 17 | 14020.9 (11865.9-20336.4) |  | 17 | 16316.3 (13056.5-27542.5) | 0.385 |
|  | LOS3 | 13 | 32729.7 (21446.2-43068.8) |  | 14 | 30506.6 (22451.3-60840.5) | 0.973 |
|  | LOS4 | 2 | 91505.4 (76592.2-106418.6) |  | 4 | 81543.4 (69801.3-100994.1) | NA |
| BMI=1 | Surgery0 | 175 | 11432.5 (6190.3-17493.1) |  | 193 | 12068.0 (6767.0-20684.0) | 0.953 |
|  | Surgery1 | 33 | 14086.1 (6244.7-34390.1) |  | 46 | 8620.0 (5335.0-13029.0) | 0.024 |
| BMI=2 | Surgery0 | 789 | 14731.0 (8570.9-30647.3) |  | 729 | 14520.0 (8402.0-26131.0) | 0.406 |
|  | Surgery1 | 178 | 11744.6 (5625.2-17561.8) |  | 189 | 10449.0 (6064.0-16616.0) | 0.298 |
| BMI=3 | Surgery0 | 205 | 17058.8 (10868.7-29994.1) |  | 222 | 16143.0 (9996.0-32582.0) | 0.762 |
|  | Surgery1 | 50 | 10871.9 (5852.2-16271.6) |  | 41 | 12955.0 (6951.0-20152.0) | 0.254 |
| BMI=4 | Surgery0 | 40 | 18403.5 (9580.5-27307.5) |  | 50 | 20269.0 (9300.0-30686.0) | 0.569 |
|  | Surgery1 | 8 | 17149.4 (13161.4-20445.4) |  | 8 | 15266.0 (5485.0-17078.0) | 0.589 |
| BMI=1 | Diagnosis1 | 162 | 10298.5 (6002.8-15475.1) |  | 180 | 11360.0 (6606.0-17512.0) | 0.851 |
|  | Diagnosis2 | 4 | 9120.6 (1522.2-21737.1) |  | 6 | 10796.0 (6395.0-14511.0) | 0.932 |
|  | Diagnosis3 | 13 | 17229.8 (12642.0-38157.8) |  | 20 | 9495.0 (4718.0-16137.0) | 0.134 |
|  | Diagnosis4 | 1 | 19212.1 (19212.1-19212.1) |  | 0 | NA | NA |
|  | Diagnosis5 | 19 | 36033.2 (19699.2-48581.4) |  | 20 | 18348.0 (11381.0-37033.0) | 0.204 |
|  | Diagnosis6 | 10 | 22641.1 (16607.4-49178.6) |  | 12 | 9518.0 (4983.0-11142.0) | 0.094 |
| BMI=2 | Diagnosis1 | 624 | 14232.9 (7808.7-25649.8) |  | 605 | 13940.0 (8242.0-22330.0) | 0.350 |
|  | Diagnosis2 | 68 | 13968.9 (6616.9-26095.4) |  | 68 | 10693.0 (6114.0-25800.0) | 0.547 |
|  | Diagnosis3 | 163 | 13446.8 (8982.5-24706.0) |  | 144 | 13477.0 (7983.0-20198.0) | 0.727 |
|  | Diagnosis4 | 18 | 14595.2 (10358.5-15474.4) |  | 17 | 15352.0 (12230.0-19633.0) | 0.230 |
|  | Diagnosis5 | 36 | 13509.1 (7710.8-26602.5) |  | 33 | 14447.0 (6064.0-28288.0) | 0.813 |
|  | Diagnosis6 | 58 | 22055.9 (13136.4-37843.0) |  | 51 | 13956.0 (6496.0-23046.0) | 0.047 |
| BMI=3 | Diagnosis1 | 143 | 17019.4 (9256.0-31076.7) |  | 155 | 16007.0 (9225.0-32028.0) | 0.365 |
|  | Diagnosis2 | 23 | 13959.3 (7995.6-23332.5) |  | 18 | 8736.0 (5628.0-13517.0) | 0.938 |
|  | Diagnosis3 | 54 | 13937.9 (10996.7-22379.5) |  | 61 | 15037.0 (11943.0-27702.0) | 0.116 |
|  | Diagnosis4 | 11 | 16818.6 (13479.3-17915.7) |  | 7 | 12410.0 (11930.0-13610.0) | 0.023 |
|  | Diagnosis5 | 7 | 19708.2 (7110.4-34363.7) |  | 2 | 67743.0 (66312.0-69175.0) | 0.553 |
|  | Diagnosis6 | 17 | 11094.9 (6254.8-18904.1) |  | 20 | 30210.0 (5825.0-34722.0) | 0.068 |
| BMI=4 | Diagnosis1 | 26 | 19756.9 (12250.5-25744.5) |  | 36 | 20635.0 (7957.0-56722.0) | 0.673 |
|  | Diagnosis2 | 4 | 10500.0 (9458.3-16414.8) |  | 6 | 21216.0 (9026.0-29952.0) | 0.537 |
|  | Diagnosis3 | 14 | 17146.4 (13805.6-24836.7) |  | 14 | 13682.0 (12220.0-19073.0) | 0.145 |
|  | Diagnosis4 | 0 | NA |  | 0 | NA | NA |
|  | Diagnosis5 | 0 | NA |  | 1 | 1424.0 (1424.0-1424.0) | NA |
|  | Diagnosis6 | 3 | 3542.5 (2801.3-8752.5) |  | 2 | 21325.0 (17870.0-24779.0) | 0.231 |
| BMI=1 | Hypertension0 | 170 | 11299.6 (6021.9-18358.3) |  | 187 | 10383.7 (5964.3-18163.8) | 0.592 |
|  | Hypertension1 | 38 | 15256.4 (8536.3-22034.9) |  | 52 | 12952.3 (8053.7-21084.2) | 0.404 |
| BMI=2 | Hypertension0 | 675 | 13864.3 (7594.5-25936.4) |  | 645 | 13547.9 (7806.5-20440.8) | 0.237 |
|  | Hypertension1 | 292 | 14974.1 (8608.2-27773.5) |  | 273 | 14431.0 (7833.0-26154.3) | 0.657 |
| BMI=3 | Hypertension0 | 138 | 15683.0 (9192.5-27840.7) |  | 142 | 14467.9 (8522.3-26034.2) | 0.389 |
|  | Hypertension1 | 117 | 15493.1 (8361.3-25830.4) |  | 121 | 16470.3 (9985.7-34116.0) | 0.211 |
| BMI=4 | Hypertension0 | 24 | 14673.8 (9349.8-21686.1) |  | 30 | 15365.9 (9115.6-30686.3) | 0.598 |
|  | Hypertension1 | 24 | 19807.4 (11691.2-39827.9) |  | 28 | 20269.3 (8259.8-29435.5) | 0.772 |
| BMI=1 | Diabetes0 | 189 | 11773.7 (6162.0-18516.1) |  | 220 | 10939.8 (6130.6-18162.3) | 0.205 |
|  | Diabetes1 | 19 | 15186.2 (7620.0-30162.3) |  | 19 | 12957.5 (8416.4-36448.2) | 0.211 |
| BMI=2 | Diabetes0 | 837 | 14194.5 (7829.6-27465.2) |  | 811 | 13764.2 (8019.6-21317.6) | 0.095 |
|  | Diabetes1 | 130 | 14466.1 (8133.2-20853.8) |  | 107 | 13733.0 (7330.7-33212.9) | 0.925 |
| BMI=3 | Diabetes0 | 214 | 15198.3 (9141.7-25734.1) |  | 222 | 15146.7 (9867.4-30897.0) | 0.764 |
|  | Diabetes1 | 41 | 19708.2 (7962.4-34285.6) |  | 41 | 16470.3 (8634.0-30790.0) | 0.718 |
| BMI=4 | Diabetes0 | 41 | 15326.8 (11324.3-25660.6) |  | 49 | 19992.2 (8453.1-30708.3) | 0.472 |
|  | Diabetes1 | 7 | 21707.9 (14465.1-33615.1) |  | 9 | 13056.5 (7195.8-19361.4) | 0.790 |
| BMI=1 | Smoking0 | 140 | 11648.6 (6125.8-21611.4) |  | 164 | 10361.4 (6091.2-18752.7) | 0.132 |
|  | Smoking1 | 68 | 12061.4 (6383.1-17199.4) |  | 75 | 11735.0 (6987.4-17828.6) | 0.290 |
| BMI=2 | Smoking0 | 570 | 14117.9 (7762.4-28330.4) |  | 561 | 13848.5 (7833.0-22534.4) | 0.137 |
|  | Smoking1 | 397 | 14662.8 (8262.5-23860.1) |  | 357 | 13376.8 (7543.3-20777.7) | 0.985 |
| BMI=3 | Smoking0 | 156 | 15307.4 (9150.0-26519.9) |  | 158 | 15086.8 (9018.7-28008.2) | 0.630 |
|  | Smoking1 | 99 | 16175.4 (7924.3-25933.8) |  | 105 | 16913.0 (8634.4-34495.2) | 0.772 |
| BMI=4 | Smoking0 | 34 | 18403.5 (12250.5-24082.5) |  | 40 | 16342.3 (8193.6-30287.5) | 0.485 |
|  | Smoking1 | 14 | 15521.6 (8594.6-30962.5) |  | 18 | 23889.6 (9525.8-28374.1) | 0.990 |
| BMI=1 | Drinking0 | 150 | 11299.6 (5985.4-17575.6) |  | 165 | 9941.7 (5921.7-18160.8) | 0.301 |
|  | Drinking1 | 58 | 13636.5 (9554.7-26314.5) |  | 74 | 12546.2 (7623.5-33351.3) | 0.752 |
| BMI=2 | Drinking0 | 577 | 13906.1 (7204.2-25801.0) |  | 563 | 13715.3 (7760.8-22809.3) | 0.809 |
|  | Drinking1 | 390 | 14780.8 (9302.5-28768.7) |  | 355 | 13813.6 (8113.7-20838.2) | 0.106 |
| BMI=3 | Drinking0 | 160 | 14426.7 (7911.5-26519.9) |  | 162 | 14653.4 (8719.0-27294.0) | 1.000 |
|  | Drinking1 | 95 | 16818.6 (10093.7-25931.7) |  | 101 | 17227.2 (9827.9-32949.2) | 0.869 |
| BMI=4 | Drinking0 | 30 | 18403.5 (11467.7-24075.2) |  | 39 | 16368.4 (10146.9-29897.7) | 0.509 |
|  | Drinking1 | 18 | 16655.3 (9023.0-30962.5) |  | 19 | 20546.5 (6483.2-29250.7) | 0.948 |
| BMI=1 | District0 | 126 | 14044.5 (6942.4-36302.0) |  | 149 | 12958.0 (8283.0-26785.0) | 0.185 |
|  | District1 | 82 | 9026.2 (5403.7-14593.9) |  | 90 | 8149.0 (5351.0-13964.0) | 0.365 |
| BMI=2 | District0 | 643 | 14147.0 (7852.3-24696.2) |  | 648 | 13944.0 (8191.0-22246.0) | 0.713 |
|  | District1 | 324 | 14481.9 (7984.7-30861.6) |  | 270 | 12871.0 (7097.0-22021.0) | 0.083 |
| BMI=3 | District0 | 172 | 14923.9 (9728.6-24595.2) |  | 182 | 15087.0 (9065.0-32582.0) | 0.328 |
|  | District1 | 83 | 17301.6 (7397.0-30780.4) |  | 81 | 15828.0 (8409.0-27702.0) | 0.236 |
| BMI=4 | District0 | 28 | 19071.6 (11850.2-27227.2) |  | 47 | 19361.0 (11943.0-30428.0) | 0.610 |
|  | District1 | 20 | 13895.8 (9349.8-22696.1) |  | 11 | 12744.0 (6677.0-25186.0) | 0.930 |
| BMI=1 | Education1 | 24 | 10045.6 (5967.5-24857.1) |  | 21 | 8832.0 (6653.0-16018.0) | 0.202 |
|  | Education2 | 68 | 10762.3 (4953.6-19322.5) |  | 77 | 11511.0 (6493.0-16533.0) | 0.626 |
|  | Education3 | 61 | 11518.8 (7118.0-17658.1) |  | 75 | 10334.0 (5504.0-16177.0) | 0.817 |
|  | Education4 | 32 | 13704.5 (6914.6-22946.0) |  | 38 | 11755.0 (7254.0-25976.0) | 0.454 |
|  | Education5 | 23 | 11919.1 (6345.7-15931.7) |  | 28 | 15104.0 (8401.0-31883.0) | 0.931 |
| BMI=2 | Education1 | 81 | 26145.2 (10692.2-57114.5) |  | 68 | 13582.0 (6248.0-25969.0) | 0.002 |
|  | Education2 | 355 | 13972.0 (7968.3-21715.6) |  | 284 | 13997.0 (8122.0-22301.0) | 0.333 |
|  | Education3 | 263 | 14735.7 (7785.4-23529.8) |  | 308 | 12899.0 (7393.0-20747.0) | 0.167 |
|  | Education4 | 136 | 14523.1 (7213.6-21693.8) |  | 138 | 15329.0 (10164.0-21147.0) | 0.661 |
|  | Education5 | 132 | 12870.4 (8384.1-27665.6) |  | 120 | 13136.0 (7972.0-22797.0) | 0.944 |
| BMI=3 | Education1 | 21 | 11376.8 (5800.7-23767.8) |  | 21 | 14494.0 (9132.0-32629.0) | 0.591 |
|  | Education2 | 74 | 17064.2 (10014.7-39115.0) |  | 79 | 15459.0 (8387.0-35978.0) | 0.853 |
|  | Education3 | 83 | 14433.0 (6789.1-24101.1) |  | 81 | 15183.0 (9318.0-27702.0) | 0.969 |
|  | Education4 | 46 | 15504.7 (11704.0-23489.6) |  | 45 | 16337.0 (10028.0-25673.0) | 0.958 |
|  | Education5 | 31 | 17702.3 (10467.9-28442.5) |  | 37 | 13877.0 (10220.0-26340.0) | 0.872 |
| BMI=4 | Education1 | 5 | 15326.8 (14057.7-18186.6) |  | 4 | 18280.0 (6554.0-29560.0) | 0.547 |
|  | Education2 | 12 | 20387.0 (10928.0-25715.4) |  | 15 | 11841.0 (5758.0-19677.0) | 0.626 |
|  | Education3 | 19 | 18879.0 (8861.4-37899.3) |  | 18 | 22025.0 (7639.0-29295.0) | 0.497 |
|  | Education4 | 5 | 19177.3 (18966.0-25772.5) |  | 13 | 28421.0 (20546.0-46575.0) | 0.059 |
|  | Education5 | 7 | 13962.4 (10627.8-14077.1) |  | 8 | 14095.0 (13499.0-27117.0) | 0.170 |
| LOS=1 | Surgery0 | 141 | 8030.3 (3622.3-14086.1) |  | 162 | 8545.0 (5140.0-13870.0) | 0.718 |
|  | Surgery1 | 612 | 9306.5 (5473.1-14071.6) |  | 591 | 9132.0 (5496.0-13529.0) | 0.659 |
| LOS=2 | Surgery0 | 86 | 12125.3 (6402.7-19530.9) |  | 84 | 10710.0 (7110.0-17075.0) | 0.317 |
|  | Surgery1 | 300 | 17015.2 (12861.2-25776.0) |  | 321 | 16368.0 (12951.0-24774.0) | 0.648 |
| LOS=3 | Surgery0 | 31 | 27548.7 (20639.2-34589.7) |  | 34 | 27128.0 (14567.0-44074.0) | 0.761 |
|  | Surgery1 | 230 | 43801.3 (30855.2-60023.1) |  | 227 | 46001.0 (31291.0-60578.0) | 0.983 |
| LOS=4 | Surgery0 | 11 | 78086.6 (56254.2-90335.7) |  | 4 | 44236.0 (36022.0-54366.0) | 0.090 |
|  | Surgery1 | 67 | 86361.8 (67067.4-120567.6) |  | 55 | 85571.0 (68323.0-100000.0) | 0.793 |
| LOS=1 | Diagnosis1 | 577 | 9389.7 (5779.3-14494.9) |  | 575 | 9651.0 (5914.0-14524.0) | 0.274 |
|  | Diagnosis2 | 31 | 4804.7 (2080.1-9701.5) |  | 39 | 6848.0 (4465.0-9326.0) | 0.879 |
|  | Diagnosis3 | 97 | 10265.5 (4477.3-12323.8) |  | 89 | 8402.0 (4446.0-12422.0) | 0.828 |
|  | Diagnosis4 | 10 | 10799.4 (6542.1-14316.5) |  | 9 | 11599.0 (9986.0-12261.0) | 0.532 |
|  | Diagnosis5 | 17 | 6193.2 (3693.5-9770.9) |  | 16 | 5836.0 (4059.0-9649.0) | 0.603 |
|  | Diagnosis6 | 21 | 5243.9 (2060.1-15773.7) |  | 25 | 4680.0 (1940.0-8947.0) | 0.253 |
| LOS=2 | Diagnosis1 | 168 | 17710.7 (11423.7-24718.6) |  | 202 | 17454.0 (12323.0-25767.0) | 0.419 |
|  | Diagnosis2 | 50 | 14300.9 (9844.3-22552.2) |  | 33 | 10195.0 (6295.0-15324.0) | 0.020 |
|  | Diagnosis3 | 88 | 15120.2 (12373.8-20441.1) |  | 100 | 14538.0 (11828.0-18396.0) | 0.393 |
|  | Diagnosis4 | 19 | 16418.4 (14326.9-22145.5) |  | 13 | 15257.0 (13571.0-17129.0) | 0.738 |
|  | Diagnosis5 | 25 | 17902.7 (11968.1-25066.7) |  | 24 | 16752.0 (12193.0-19851.0) | 0.522 |
|  | Diagnosis6 | 36 | 17648.5 (9979.2-28285.3) |  | 33 | 13956.0 (7820.0-21849.0) | 0.130 |
| LOS=3 | Diagnosis1 | 156 | 46759.4 (33666.9-62236.7) |  | 159 | 49824.0 (35429.0-64066.0) | 0.874 |
|  | Diagnosis2 | 16 | 32847.5 (25809.5-38373.3) |  | 22 | 32635.0 (28447.0-37473.0) | 0.857 |
|  | Diagnosis3 | 49 | 32657.3 (23754.9-43068.8) |  | 43 | 29710.0 (22177.0-43326.0) | 0.965 |
|  | Diagnosis4 | 1 | 12872.1 (12872.1-12872.1) |  | 3 | 37617.0 (34976.0-83345.0) | NA |
|  | Diagnosis5 | 12 | 36851.4 (27017.3-44625.2) |  | 11 | 55039.0 (38036.0-69112.0) | 0.056 |
|  | Diagnosis6 | 27 | 33847.4 (21524.8-60285.0) |  | 23 | 34116.0 (16911.0-47187.0) | 0.270 |
| LOS=4 | Diagnosis1 | 54 | 88433.4 (69033.9-119754.0) |  | 40 | 85313.0 (63831.0-100000.0) | 0.538 |
|  | Diagnosis2 | 2 | 89315.4 (79067.2-99563.5) |  | 4 | 100000.0 (81565.0-200000.0) | NA |
|  | Diagnosis3 | 10 | 66988.5 (60538.0-85441.2) |  | 7 | 80087.0 (71295.0-100000.0) | 0.420 |
|  | Diagnosis4 | 0 | NA |  | 0 | NA | NA |
|  | Diagnosis5 | 8 | 98442.2 (59389.3-117727.5) |  | 4 | 72083.0 (65291.0-85212.0) | 0.410 |
|  | Diagnosis6 | 4 | 61967.4 (47003.8-149530.6) |  | 4 | 71895.0 (58810.0-81178.0) | 0.471 |
| LOS=1 | Hypertension0 | 524 | 8770 (5134.6-13543.2) |  | 538 | 9021.8 (5402.8-13504.7) | 0.328 |
|  | Hypertension1 | 229 | 10537.1 (5665.8-15021.0) |  | 215 | 9132.1 (5456.9-14635.0) | 0.564 |
| LOS=2 | Hypertension0 | 261 | 15623.1 (11286.2-24441.4) |  | 271 | 15479.2 (11040.8-21837.9) | 0.510 |
|  | Hypertension1 | 125 | 16856.3 (12351.4-23860.1) |  | 134 | 15652.0 (10831.6-25766.9) | 0.800 |
| LOS=3 | Hypertension0 | 173 | 43223.5 (28288.4-57122) |  | 154 | 41708.6 (29422.7-58128.1) | 0.565 |
|  | Hypertension1 | 88 | 37411.2 (27157-59241.5) |  | 107 | 45768.3 (29338.1-61881.9) | 0.487 |
| LOS=4 | Hypertension0 | 49 | 82825.9 (62994.4-112434.2) |  | 41 | 78364.9 (58294.4-103208.7) | 0.780 |
|  | Hypertension1 | 29 | 88167.9 (68819.1-121331.8) |  | 18 | 101115.0 (68143.4-132031.9) | 0.993 |
| LOS=1 | Diabetes0 | 643 | 9210.5 (5165.5-13891.7) |  | 669 | 9247.5 (5499.5-13712.0) | 0.252 |
|  | Diabetes1 | 110 | 8936.1 (5504-14925.7) |  | 84 | 7858.2 (4570.9-12721.5) | 0.094 |
| LOS=2 | Diabetes0 | 341 | 15798.0 (11365-23631.7) |  | 358 | 15665.6 (11196.0-22478.0) | 0.773 |
|  | Diabetes1 | 45 | 18728.4 (13838.9-28315.1) |  | 47 | 14921.1 (10238.8-26302.7) | 0.269 |
| LOS=3 | Diabetes0 | 228 | 41973 (27605.9-57586.0) |  | 227 | 41919.9 (28616.1-57810.4) | 0.447 |
|  | Diabetes1 | 33 | 40903.3 (28798.1-58678.4) |  | 34 | 53127.8 (33272.4-73400.8) | 0.031 |
| LOS=4 | Diabetes0 | 69 | 85457.5 (66489-120058.6) |  | 48 | 85010.8 (69175.9-126202.4) | 0.867 |
|  | Diabetes1 | 9 | 85321 (70983.3-95992.3) |  | 11 | 77703.8 (63424.2-91550.8) | 0.926 |
| LOS=1 | Smoking0 | 428 | 8543.9 (4760.6-13700.9) |  | 451 | 8650.4 (5297.9-13460.0) | 0.368 |
|  | Smoking1 | 325 | 9760.5 (5963.6-14825.9) |  | 302 | 9536.3 (5709.7-13924.6) | 0.752 |
| LOS=2 | Smoking0 | 251 | 15336.0 (11370.9-22138.6) |  | 280 | 15477.8 (10990.0-22425.4) | 0.922 |
|  | Smoking1 | 135 | 18057.0 (11917.0-25694.9) |  | 125 | 15797.7 (10786.8-24178.1) | 0.256 |
| LOS=3 | Smoking0 | 171 | 39497.3 (27526.4-55752.3) |  | 158 | 40391.5 (29116.0-58452.9) | 0.396 |
|  | Smoking1 | 90 | 44478.3 (27981.0-60002.9) |  | 103 | 45768.3 (30681.0-59834.6) | 0.325 |
| LOS=4 | Smoking0 | 50 | 90201.1 (67820.7-123446.7) |  | 34 | 79794.9 (59169.2-124585.3) | 0.355 |
|  | Smoking1 | 28 | 74669.4 (61377.9-101346.9) |  | 25 | 84450.2 (66039.7-95752.0) | 0.251 |
| LOS=1 | Drinking0 | 465 | 8030.3 (4881.5-13845.6) |  | 461 | 8645.7 (5109.4-13359.5) | 0.428 |
|  | Drinking1 | 288 | 10548.5 (5958.1-14549.0) |  | 292 | 9793.6 (6036.0-13919.1) | 0.748 |
| LOS=2 | Drinking0 | 244 | 15364.7 (11362.7-22463.7) |  | 275 | 15036.5 (10534.2-22341.9) | 0.989 |
|  | Drinking1 | 142 | 17393.9 (12193.9-25601.6) |  | 130 | 16641.7 (12214.4-25427.4) | 0.298 |
| LOS=3 | Drinking0 | 165 | 39497.3 (25746.8-55757.0) |  | 159 | 42389.7 (29451.1-60517.3) | 0.435 |
|  | Drinking1 | 96 | 45316.4 (33338.7-60893.9) |  | 102 | 43371.1 (28522.4-56206.8) | 0.291 |
| LOS=4 | Drinking0 | 43 | 90141.7 (69670.4-120567.6) |  | 34 | 83858.9 (56387.0-131643.8) | 0.779 |
|  | Drinking1 | 35 | 80227.5 (61814.4-107337.5) |  | 25 | 83964.2 (71514.0-95752.0) | 0.857 |
| LOS=1 | District0 | 258 | 8108.2 (4996.5-13323.7) |  | 244 | 8225.0 (5301.0-12338.0) | 0.798 |
|  | District1 | 495 | 10002.7 (5347.3-14220.9) |  | 509 | 9825.0 (5516.0-14031.0) | 0.516 |
| LOS=2 | District0 | 134 | 16317.7 (11399.2-25866.2) |  | 120 | 15357.0 (9023.0-21293.0) | 0.068 |
|  | District1 | 252 | 15869.6 (11697.4-22498.1) |  | 285 | 15798.0 (11572.0-22942.0) | 0.632 |
| LOS=3 | District0 | 93 | 39497.3 (28288.4-59085.2) |  | 79 | 46558.0 (29196.0-57671.0) | 0.438 |
|  | District1 | 168 | 42617.4 (27605.9-57231.5) |  | 182 | 41344.0 (29558.0-60841.0) | 0.641 |
| LOS=4 | District0 | 24 | 83638.1 (61581.0-105037.6) |  | 9 | 86176.0 (66040.0-100000.0) | 0.609 |
|  | District1 | 54 | 87264.8 (67197.2-119754.0) |  | 50 | 82026.0 (64401.0-100000.0) | 0.834 |
| LOS=1 | Education1 | 42 | 5936.8 (4230.4-8966.4) |  | 54 | 7044.9 (4748.9-12782.5) | 0.099 |
|  | Education2 | 277 | 9623.0 (5160.3-14420.4) |  | 224 | 8880.1 (5734.4-13843.4) | 0.872 |
|  | Education3 | 229 | 8872.8 (5395.6-14052.8) |  | 275 | 9100.9 (5108.9-12897.1) | 0.375 |
|  | Education4 | 114 | 10038.9 (5382.2-14773) |  | 112 | 11481.8 (6566.4-15437.1) | 0.197 |
|  | Education5 | 91 | 10520.1 (5099.0-13577.2) |  | 88 | 10169.5 (5669.7-14172.5) | 0.519 |
| LOS=2 | Education1 | 37 | 14147.0 (11376.8-20433.1) |  | 31 | 14493.9 (9499.1-19942.7) | 0.975 |
|  | Education2 | 119 | 16856.3 (11321.0-25694.9) |  | 122 | 14511.7 (9228.9-21082.8) | 0.197 |
|  | Education3 | 114 | 17729.6 (12771.8-23480.4) |  | 119 | 16667.1 (12940.8-24568.2) | 0.526 |
|  | Education4 | 56 | 15551.0 (13032.7-22932.3) |  | 73 | 16336.9 (11716.2-22284.7) | 0.959 |
|  | Education5 | 60 | 13991.7 (10903.5-24469.5) |  | 60 | 15370.8 (11189.0-20580.2) | 0.499 |
| LOS=3 | Education1 | 40 | 49056.3 (33187.1-60515.6) |  | 27 | 35868.4 (29770.8-53132.6) | NA |
|  | Education2 | 92 | 41290.5 (29856.5-53909.3) |  | 89 | 42037.3 (25929.4-56264.3) | 0.295 |
|  | Education3 | 58 | 45423.3 (29398.8-59960.3) |  | 71 | 53035.9 (36795.8-65011.1) | 0.923 |
|  | Education4 | 40 | 35188.3 (24174.7-46514.1) |  | 40 | 43243.1 (30770.1-56222.2) | 0.892 |
|  | Education5 | 31 | 36706.6 (26519.2-60652.1) |  | 34 | 31993.0 (13627.8-50036.0) | 0.252 |
| LOS=4 | Education1 | 12 | 96864.1 (77983.6-119399.3) |  | 2 | 60614.5 (52377.7-68851.3) | 0.415 |
|  | Education2 | 21 | 91905.9 (74440.7-112434.2) |  | 20 | 104232.8 (61530.8-144133.9) | 0.636 |
|  | Education3 | 25 | 78155.7 (62417.9-104863.3) |  | 17 | 79502.4 (64886.9-103208.7) | 0.279 |
|  | Education4 | 9 | 62994.4 (49513.9-113694.4) |  | 9 | 74454.4 (45987.6-77750.3) | 0.538 |
|  | Education5 | 11 | 85321.0 (69004.9-134275.4) |  | 11 | 91461.1 (78762.0-109922.2) | 0.662 |
| Surgery=0 | Diagnosis1 | 146 | 13588.1 (6671.7-18286.7) |  | 157 | 11399.0 (7536.0-16695.0) | 0.328 |
|  | Diagnosis2 | 17 | 3463.1 (1629.3-10345.2) |  | 25 | 4629.0 (3591.0-9828.0) | 0.884 |
|  | Diagnosis3 | 50 | 10739.8 (5368.2-23654.4) |  | 49 | 8661.0 (5771.0-16568.0) | 0.936 |
|  | Diagnosis4 | 2 | 7729.3 (5011.9-10446.6) |  | 1 | 2723.0 (2723.0-2723.0) | NA |
|  | Diagnosis5 | 29 | 13703.1 (8030.3-23297.5) |  | 25 | 13217.0 (6064.0-18167.0) | 0.258 |
|  | Diagnosis6 | 25 | 6504.0 (3164.9-13249.1) |  | 27 | 9193.0 (4966.0-15124.0) | 0.178 |
| Surgery=1 | Diagnosis1 | 809 | 14105.3 (7399.0-26037.2) |  | 819 | 14520.0 (8127.0-28499.0) | 0.795 |
|  | Diagnosis2 | 82 | 14581.3 (9084.4-26907.4) |  | 73 | 12951.0 (7668.0-29365.0) | 0.383 |
|  | Diagnosis3 | 194 | 14123.8 (11430.2-25794.1) |  | 190 | 14439.0 (11019.0-22541.0) | 0.808 |
|  | Diagnosis4 | 28 | 14706.8 (12427.9-17165.3) |  | 24 | 14605.0 (12254.0-17650.0) | 0.287 |
|  | Diagnosis5 | 33 | 30647.3 (11090.4-48574.1) |  | 30 | 34768.0 (9912.0-58217.0) | 0.696 |
|  | Diagnosis6 | 63 | 24449.9 (17532.9-40878.1) |  | 58 | 17885.0 (7760.0-30884.0) | 0.041 |
| Surgery=0 | Hypertension0 | 166 | 11771.8 (4860.0-17719.0) |  | 185 | 10194.7 (6139.3-16347.8) | 0.576 |
|  | Hypertension1 | 103 | 12359.8 (6451.1-20107.8) |  | 99 | 10786.8 (5814.3-17198.9) | 0.329 |
| Surgery=1 | Hypertension0 | 841 | 13906.2 (7809.2-27529.2) |  | 819 | 13795.1 (8034.8-25084.4) | 0.401 |
|  | Hypertension1 | 368 | 16039.2 (10546.1-30765.0) |  | 375 | 16193.0 (8896.8-32933.4) | 0.878 |
| Surgery=0 | Diabetes0 | 214 | 11331.5 (5330.6-17561.8) |  | 246 | 10718.0 (6134.1-16552.9) | 0.886 |
|  | Diabetes1 | 55 | 14344.7 (8602.0-20250.0) |  | 38 | 8910.6 (5391.3-18141.9) | 0.166 |
| Surgery=1 | Diabetes0 | 1067 | 14517.9 (8467.6-27932.9) |  | 1056 | 14433.3 (8367.9-26507.0) | 0.222 |
|  | Diabetes1 | 142 | 15043.0 (7908.4-30722.2) |  | 138 | 15224.9 (8228.6-36956.9) | 0.115 |
| Surgery=0 | Smoking0 | 159 | 12351.4 (4289.3-18757.4) |  | 166 | 9996.1 (5792.4-16327.0) | 0.216 |
|  | Smoking1 | 110 | 12010.7 (6737.7-18286.7) |  | 118 | 11178.4 (6701.0-17068.2) | 0.160 |
| Surgery=1 | Smoking0 | 741 | 14517.9 (8361.3-30964.8) |  | 757 | 14493.9 (8409.5-26932.2) | 0.166 |
|  | Smoking1 | 468 | 14850.3 (8383.6-25690.9) |  | 437 | 14566.7 (8207.9-30387.4) | 0.453 |
| Surgery=0 | Drinking0 | 163 | 11790.2 (4929.0-17535.1) |  | 168 | 10523.4 (6049.7-16564.5) | 0.332 |
|  | Drinking1 | 106 | 12614.6 (6439.3-20200.3) |  | 116 | 10458.6 (6032.5-16620.9) | 0.072 |
| Surgery=1 | Drinking0 | 754 | 14130.7 (7495.6-26678.3) |  | 761 | 14056.7 (8115.5-27524.9) | 0.827 |
|  | Drinking1 | 455 | 15348.8 (9935.3-33304) |  | 433 | 15288.4 (8878.8-30387.4) | 0.432 |
| Surgery=0 | District0 | 89 | 8440.8 (4022.3-15731.9) |  | 88 | 10216.0 (6057.0-16314.0) | 0.783 |
|  | District1 | 180 | 13324.8 (6337.8-20014.4) |  | 196 | 10640.0 (5894.0-16650.0) | 0.035 |
| Surgery=1 | District0 | 420 | 14745.6 (8079.4-30163.6) |  | 364 | 12769.0 (6702.0-24361.0) | 0.049 |
|  | District1 | 789 | 14683.5 (8502.8-27529.2) |  | 830 | 15101.0 (9176.0-30346.0) | 0.792 |
| Surgery=0 | Education1 | 20 | 14210.2 (10041.7-21861.1) |  | 26 | 6465.0 (4843.0-13173.0) | 0.006 |
|  | Education2 | 111 | 11828.0 (5972.3-17104.1) |  | 95 | 11475.0 (8054.0-16602.0) | 0.323 |
|  | Education3 | 67 | 12466.9 (5448.7-17798.0) |  | 90 | 10208.0 (5713.0-17012.0) | 0.597 |
|  | Education4 | 38 | 13643.2 (5381.0-24141.5) |  | 37 | 11716.0 (6927.0-17319.0) | 0.178 |
|  | Education5 | 33 | 11249.7 (4370.1-21146.8) |  | 36 | 8572.0 (5038.0-12972.0) | 0.330 |
| Surgery=1 | Education1 | 111 | 15244.1 (7809.9-50397.7) |  | 88 | 14568.0 (8525.0-32872.0) | 0.218 |
|  | Education2 | 398 | 14678.5 (8327.2-29597.9) |  | 360 | 14089.0 (7780.0-30358.0) | 0.714 |
|  | Education3 | 359 | 14739.8 (8034.1-24227.7) |  | 392 | 13399.0 (7767.0-26085.0) | 0.262 |
|  | Education4 | 181 | 14870.2 (8683.0-22811.6) |  | 197 | 16115.0 (10028.0-27643.0) | 0.223 |
|  | Education5 | 160 | 13637.3 (9573.6-28959.6) |  | 157 | 15089.0 (9921.0-28110.0) | 0.630 |
| Diagnosis=1 | Hypertension0 | 649 | 12836.0 (6869.7-21741.8) |  | 671 | 13430.5 (7626.6-21481.5) | 0.756 |
|  | Hypertension1 | 306 | 15633.4 (9737.3-29728.5) |  | 305 | 15288.4 (8400.9-27643.3) | 0.379 |
| Diagnosis=2 | Hypertension0 | 62 | 14421.8 (8349.7-26907.4) |  | 66 | 9670.6 (5517.7-17312.6) | 0.886 |
|  | Hypertension1 | 37 | 11502.1 (7009.1-20848.2) |  | 32 | 12030.5 (6294.3-26634.5) | 0.427 |
| Diagnosis=3 | Hypertension0 | 178 | 13674.5 (10114.9-23112) |  | 159 | 13063.4 (8517.8-18133.5) | 0.742 |
|  | Hypertension1 | 66 | 14161.5 (10416.6-29198.8) |  | 80 | 14962.0 (8581.6-26425.4) | 0.276 |
| Diagnosis=4 | Hypertension0 | 18 | 14423.8 (10358.5-16189.6) |  | 16 | 14414.0 (12072.5-16716.9) | 0.224 |
|  | Hypertension1 | 12 | 15369.2 (13521.7-20272.8) |  | 9 | 13951.9 (12261.3-19212.1) | 0.874 |
| Diagnosis=5 | Hypertension0 | 44 | 24527.5 (11664.9-38461.6) |  | 40 | 15212.8 (6041.1-29498.3) | 0.088 |
|  | Hypertension1 | 18 | 11083.6 (7054.7-21657.4) |  | 15 | 16837.3 (13114.5-52192.4) | 0.053 |
| Diagnosis=6 | Hypertension0 | 56 | 21551.3 (7349.9-38887.9) |  | 52 | 11199.9 (7003.4-21865.6) | 0.016 |
|  | Hypertension1 | 32 | 17983.0 (10877.8-26799.3) |  | 33 | 19406.3 (5235.4-39235.8) | 0.810 |
| Diagnosis=1 | Diabetes0 | 821 | 13838.2 (7313.0-23860.1) |  | 854 | 13847.8 (8101.8-22526.9) | 0.212 |
|  | Diabetes1 | 134 | 14861.4 (7908.4-24219.1) |  | 122 | 13681.1 (7601.6-31990.2) | 0.315 |
| Diagnosis=2 | Diabetes0 | 83 | 13965.7 (7475.7-26262.2) |  | 89 | 10756.3 (6293.1-24942.8) | 0.563 |
|  | Diabetes1 | 16 | 13002.9 (5822.6-20566.7) |  | 9 | 7833.0 (4536.2-8972.7) | 0.524 |
| Diagnosis=3 | Diabetes0 | 217 | 13733.8 (10151.1-23366.7) |  | 213 | 13931.9 (8650.4-22268.7) | 0.955 |
|  | Diabetes1 | 27 | 14927.2 (10141.8-33147.2) |  | 26 | 12863.4 (8417.7-16623.7) | 0.321 |
| Diagnosis=4 | Diabetes0 | 26 | 14698.2 (11168.5-17006.9) |  | 23 | 13649.3 (11914.6-16458.2) | 0.384 |
|  | Diabetes1 | 4 | 13786.4 (12427.9-22655.7) |  | 2 | 40936.8 (36636.2-45237.3) | NA |
| Diagnosis=5 | Diabetes0 | 56 | 19699.2 (9594.0-35739.5) |  | 48 | 16197.9 (7125.8-36560.2) | 0.461 |
|  | Diabetes1 | 6 | 17221.9 (9655.7-27912.5) |  | 7 | 20080.5 (14779.1-50915.9) | 0.585 |
| Diagnosis=6 | Diabetes0 | 78 | 19639.4 (7412.3-36932.7) |  | 75 | 11650.4 (5690.1-26204.5) | 0.024 |
|  | Diabetes1 | 10 | 19937.6 (12301.7-31034.8) |  | 10 | 42107.1 (11622.9-63306.0) | 0.280 |
| Diagnosis=1 | Smoking0 | 525 | 14046.3 (7214.8-29283.0) |  | 555 | 13948.0 (7898.0-23582.9) | 0.784 |
|  | Smoking1 | 430 | 14019.8 (7609.1-21694.3) |  | 421 | 13582.6 (8028.0-25204.2) | 0.516 |
| Diagnosis=2 | Smoking0 | 74 | 13968.9 (7667.5-26470.8) |  | 72 | 10730.4 (6229.0-25132.9) | 0.954 |
|  | Smoking1 | 25 | 12380.0 (6111.4-21489.7) |  | 26 | 9670.6 (5232.1-19414.3) | 0.476 |
| Diagnosis=3 | Smoking0 | 185 | 13458.6 (9137.5-20468.8) |  | 193 | 13764.2 (8628.1-21410.7) | 0.993 |
|  | Smoking1 | 59 | 15531.3 (11810.5-27630.5) |  | 46 | 14500.4 (7378.3-24299.0) | 0.616 |
| Diagnosis=4 | Smoking0 | 23 | 14683.5 (12129.7-17261.0) |  | 15 | 15257.2 (12580.4-19422.7) | 0.325 |
|  | Smoking1 | 7 | 14700.7 (11107.0-15721.6) |  | 10 | 13029.8 (11764.5-15686.3) | 0.382 |
| Diagnosis=5 | Smoking0 | 40 | 21860.5 (9594.0-36148.6) |  | 43 | 17319.1 (7565.8-37310.6) | 0.985 |
|  | Smoking1 | 22 | 15197.1 (8493.6-29252.1) |  | 12 | 14077.6 (7629.5-21270.4) | 0.246 |
| Diagnosis=6 | Smoking0 | 53 | 19108.2 (7082.6-34409.5) |  | 45 | 9842.7 (5226.4-20135.4) | 0.088 |
|  | Smoking1 | 35 | 20170.5 (9132.5-37948.0) |  | 40 | 18689.1 (7569.5-34867.6) | 0.478 |
| Diagnosis=1 | Drinking0 | 553 | 13529.8 (6643.9-22166.8) |  | 576 | 13764.5 (7822.9-22820.9) | 0.621 |
|  | Drinking1 | 402 | 14861.4 (9238.3-25751.3) |  | 400 | 13944.2 (8136.6-25659.4) | 0.447 |
| Diagnosis=2 | Drinking0 | 75 | 13965.7 (8263.6-26262.2) |  | 66 | 10412.1 (5517.7-25804.3) | 0.957 |
|  | Drinking1 | 24 | 12730.7 (4067.3-16671.2) |  | 32 | 10017.0 (6849.3-19637.1) | 0.488 |
| Diagnosis=3 | Drinking0 | 172 | 13674.5 (9815.6-22699.5) |  | 173 | 13184.7 (8401.5-20767.3) | 0.999 |
|  | Drinking1 | 72 | 14517.1 (11419.9-26373.1) |  | 66 | 15344.8 (10557.6-25309.2) | 0.622 |
| Diagnosis=4 | Drinking0 | 23 | 14506.9 (11983.7-16744.1) |  | 13 | 13951.9 (12230.3-19633.3) | 0.272 |
|  | Drinking1 | 7 | 15388.5 (12406.8-17135.4) |  | 12 | 14453.3 (11894.7-15993.1) | 0.663 |
| Diagnosis=5 | Drinking0 | 38 | 19341.6 (7979.4-33801.1) |  | 41 | 16837.3 (6064.5-36404.7) | 0.852 |
|  | Drinking1 | 24 | 19985.7 (11087.0-35739.5) |  | 14 | 15666.2 (9649.4-37046.4) | 0.573 |
| Diagnosis=6 | Drinking0 | 56 | 18268.1 (6861.0-31788.3) |  | 60 | 10714.5 (6157.8-28552.2) | 0.302 |
|  | Drinking1 | 32 | 30261.0 (11733.4-39278.3) |  | 25 | 18062.2 (6067.1-31906.9) | 0.171 |
| Diagnosis=1 | District0 | 346 | 12794.3 (7146.9-21694.2) |  | 314 | 12138.0 (6955.0-19327.0) | 0.326 |
|  | District1 | 609 | 14550.7 (7566.2-25774.2) |  | 662 | 14828.0 (8373.0-27158.0) | 0.549 |
| Diagnosis=2 | District0 | 30 | 14715.7 (9086.6-25823.0) |  | 30 | 8197.0 (4473.0-28815.0) | 0.873 |
|  | District1 | 69 | 12380.0 (6111.4-21496.5) |  | 68 | 11020.0 (6926.0-18654.0) | 0.577 |
| Diagnosis=3 | District0 | 73 | 15244.1 (7016.2-32008.7) |  | 64 | 12897.0 (5760.0-27227.0) | 0.310 |
|  | District1 | 171 | 13615.1 (10568.0-20138.8) |  | 175 | 13932.0 (10491.0-20613.0) | 0.872 |
| Diagnosis=4 | District0 | 5 | 25953.7 (25911.8-26631.0) |  | 6 | 13801.0 (13590.0-15923.0) | 0.814 |
|  | District1 | 25 | 14147.0 (10503.3-15503.0) |  | 19 | 15257.0 (11197.0-18171.0) | 0.136 |
| Diagnosis=5 | District0 | 24 | 24527.5 (11745.3-36148.6) |  | 14 | 15666.0 (6305.0-19697.0) | 0.139 |
|  | District1 | 38 | 18151.5 (7979.4-29872.6) |  | 41 | 16667.0 (8106.0-37568.0) | 0.590 |
| Diagnosis=6 | District0 | 31 | 21002.9 (7191.7-35002.1) |  | 24 | 15754.0 (6889.0-23465.0) | 0.254 |
|  | District1 | 57 | 18904.1 (11286.2-36401.1) |  | 61 | 12034.0 (5313.0-30790.0) | 0.154 |
| Diagnosis=1 | Education1 | 81 | 15210.3 (6891.7-60455.2) |  | 63 | 12144.0 (6710.0-23767.0) | 0.061 |
|  | Education2 | 346 | 14028.4 (7326.7-20278.7) |  | 315 | 13021.0 (7814.0-20954.0) | 0.634 |
|  | Education3 | 290 | 13343.2 (7088.1-21403.9) |  | 341 | 13109.0 (7512.0-23856.0) | 0.409 |
|  | Education4 | 140 | 14832.0 (7381.7-24367.6) |  | 153 | 16759.0 (10009.0-27643.0) | 0.688 |
|  | Education5 | 98 | 14386.9 (9344.1-30722.1) |  | 104 | 16443.0 (10977.0-27671.0) | 0.682 |
| Diagnosis=2 | Education1 | 9 | 15003.9 (12046.5-31457.1) |  | 13 | 10852.0 (6951.0-29394.0) | 0.352 |
|  | Education2 | 36 | 12724.8 (6041.9-20918.6) |  | 35 | 11511.0 (6375.0-29784.0) | 0.309 |
|  | Education3 | 19 | 11793.3 (6243.5-20686.7) |  | 28 | 10430.0 (5498.0-16233.0) | 0.494 |
|  | Education4 | 17 | 15611.4 (9099.4-25830.4) |  | 10 | 8968.0 (7683.0-11330.0) | 0.103 |
|  | Education5 | 18 | 11110.4 (4183.1-31519.3) |  | 12 | 7434.0 (5038.0-15370.0) | 0.518 |
| Diagnosis=3 | Education1 | 24 | 14716.9 (10265.4-32863.2) |  | 23 | 15904.0 (9416.0-33136.0) | 0.969 |
|  | Education2 | 63 | 15358.5 (11362.7-34191.8) |  | 69 | 14821.0 (8849.0-22399.0) | 0.782 |
|  | Education3 | 63 | 14934.4 (11216.1-23352.1) |  | 66 | 12897.0 (6952.0-17296.0) | 0.296 |
|  | Education4 | 39 | 13906.2 (9131.5-16634.3) |  | 39 | 13063.0 (10952.0-18405.0) | 0.406 |
|  | Education5 | 55 | 12045.2 (9756.2-16239.2) |  | 42 | 12563.0 (9180.0-20690.0) | 0.502 |
| Diagnosis=4 | Education1 | 6 | 20297.7 (14281.1-26451.2) |  | 2 | 16582.0 (15267.0-17897.0) | NA |
|  | Education2 | 7 | 15388.5 (13792.5-21511.7) |  | 4 | 13610.0 (12530.0-22621.0) | 0.695 |
|  | Education3 | 10 | 14533.5 (11168.5-16327.5) |  | 8 | 14094.0 (12365.0-16717.0) | 0.350 |
|  | Education4 | 6 | 12801.1 (7534.2-14652.2) |  | 5 | 15798.0 (15352.0-16337.0) | 0.174 |
|  | Education5 | 1 | 2294.6 (2294.6-2294.6) |  | 6 | 11528.0 (9041.0-17790.0) | NA |
| Diagnosis=5 | Education1 | 5 | 18974.9 (17328.0-20423.6) |  | 6 | 13130.0 (7739.0-15318.0) | 0.267 |
|  | Education2 | 29 | 19708.2 (8030.3-37669.7) |  | 11 | 19461.0 (10420.0-41599.0) | 0.640 |
|  | Education3 | 14 | 17831.6 (11733.4-27586.5) |  | 19 | 16667.0 (6612.0-37311.0) | 0.304 |
|  | Education4 | 6 | 42919.1 (22311.5-58071.8) |  | 6 | 16848.0 (15428.0-45609.0) | 0.381 |
|  | Education5 | 8 | 17790.0 (5903.8-27398.0) |  | 13 | 16837.0 (5701.0-37027.0) | 0.367 |
| Diagnosis=6 | Education1 | 6 | 13183.9 (5878.4-39491.5) |  | 7 | 7701.0 (4268.0-20320.0) | 0.527 |
|  | Education2 | 28 | 18481.7 (11848.5-36356.8) |  | 21 | 20135.0 (7820.0-39236.0) | 0.802 |
|  | Education3 | 30 | 21712.8 (7941.5-37250.6) |  | 20 | 13731.0 (5825.0-23465.0) | 0.065 |
|  | Education4 | 11 | 21002.9 (4204.4-23899.4) |  | 21 | 15832.0 (7163.0-30997.0) | 0.984 |
|  | Education5 | 13 | 17828.2 (11094.9-33229.8) |  | 16 | 8931.0 (5021.0-11551.0) | 0.321 |
| Hypertension=0 | Diabetes0 | 923 | 13535.6 (7283.9-25634.5) |  | 942 | 13202.9 (7572.0-20831.5) | 0.425 |
|  | Diabetes1 | 84 | 14589.2 (7048.3-22610.2) |  | 62 | 13001.4 (7449.3-27621.9) | 0.176 |
| Hypertension=1 | Diabetes0 | 358 | 15340.7 (9170.2-26016.3) |  | 360 | 15151.0 (8599.9-27681.2) | 0.598 |
|  | Diabetes1 | 113 | 15107.7 (8504.0-30747.2) |  | 114 | 14630.2 (7665.8-32600.6) | 0.619 |
| Hypertension=0 | Smoking0 | 635 | 13615.1 (7013.1-26298.6) |  | 648 | 13080.1 (7536.3-21241.6) | 0.179 |
|  | Smoking1 | 372 | 13471.2 (7736.0-23433.0) |  | 356 | 13358.5 (7878.4-21292.8) | 0.648 |
| Hypertension=1 | Smoking0 | 265 | 15107.7 (8954.2-28787.4) |  | 275 | 15230.4 (8425.9-29506.4) | 0.314 |
|  | Smoking1 | 206 | 15561.3 (8777.8-24912.5) |  | 199 | 15026.2 (8045.1-29510.6) | 0.339 |
| Hypertension=0 | Drinking0 | 644 | 13258.2 (6691.7-24497.7) |  | 649 | 13044.9 (7497.4-20723.2) | 0.590 |
|  | Drinking1 | 363 | 14685.4 (8759.0-25634.5) |  | 355 | 13642.0 (8003.8-21417.7) | 0.129 |
| Hypertension=1 | Drinking0 | 273 | 14927.2 (7906.6-23767.8) |  | 280 | 14876.4 (8203.7-28539.0) | 0.905 |
|  | Drinking1 | 198 | 16264.3 (10610.9-33588.3) |  | 194 | 15405.3 (8456.9-30438.5) | 0.773 |
| Hypertension=0 | District0 | 367 | 13284.6 (7060.6-25730.8) |  | 339 | 12132.4 (6253.6-19773.2) | 0.093 |
|  | District1 | 640 | 13800.0 (7394.8-24469.5) |  | 665 | 13733.0 (8207.9-21914.5) | 0.603 |
| Hypertension=1 | District0 | 142 | 15490.7 (7952.2-27137.3) |  | 113 | 13583.7 (7084.0-25673.3) | 0.368 |
|  | District1 | 329 | 15145.3 (9769.1-26753.3) |  | 361 | 15607.0 (8848.6-30996.7) | 0.965 |
| Hypertension=0 | Education1 | 83 | 14235.5 (7112.5-47874.3) |  | 68 | 13015.4 (6738.2-22941.2) | 0.086 |
|  | Education2 | 306 | 13930.1 (6737.5-23058.1) |  | 295 | 13842.1 (7898.0-21177.5) | 0.913 |
|  | Education3 | 311 | 13529.8 (7176.1-22862.2) |  | 339 | 12447.1 (6891.3-20303.4) | 0.276 |
|  | Education4 | 153 | 14344.7 (7440.5-21593.6) |  | 164 | 14103.4 (8646.3-21956.6) | 0.957 |
|  | Education5 | 154 | 12507.6 (8155.4-26226.4) |  | 138 | 13018.0 (8354.9-19418.3) | 0.928 |
| Hypertension=1 | Education1 | 48 | 18203.6 (10107.8-53179.4) |  | 46 | 13938.3 (7079.3-30943.5) | 0.085 |
|  | Education2 | 203 | 14494.9 (8585.4-25870.9) |  | 160 | 13731.5 (7724.9-34932.4) | 0.374 |
|  | Education3 | 115 | 16137.4 (8227.5-22579.1) |  | 143 | 15026.2 (8447.7-27668.8) | 0.403 |
|  | Education4 | 66 | 15258.1 (7567.1-25485.2) |  | 70 | 17932.4 (11180.8-27150.8) | 0.339 |
|  | Education5 | 39 | 14731.0 (11401.0-27174.7) |  | 55 | 16911.1 (11459.1-29008.6) | 0.958 |
| Diabetes=0 | Smoking0 | 796 | 13975.9 (7304.8-26998.3) |  | 820 | 13837.7 (7805.9-22511.8) | 0.601 |
|  | Smoking1 | 485 | 14162.6 (8010.9-23428.0) |  | 482 | 13443.3 (7960.4-22893.0) | 0.781 |
| Diabetes=1 | Smoking0 | 104 | 14897.1 (8342.3-25852.4) |  | 103 | 13045.3 (7578.1-29338.1) | 0.636 |
|  | Smoking1 | 93 | 14735.7 (7962.4-25781.5) |  | 73 | 15143.3 (7883.4-32516.1) | 0.505 |
| Diabetes=0 | Drinking0 | 798 | 13714.8 (6965.8-25303.1) |  | 814 | 13460.0 (7565.4-22102.0) | 0.326 |
|  | Drinking1 | 483 | 14827.2 (9361.2-27443.8) |  | 488 | 13952.5 (8336.7-25160.9) | 0.143 |
| Diabetes=1 | Drinking0 | 119 | 14431.3 (7628.8-21108.9) |  | 115 | 14446.6 (8012.5-32482.2) | 0.167 |
|  | Drinking1 | 78 | 15887.1 (9155.8-30722.2) |  | 61 | 13813.6 (7195.8-29961.2) | 0.683 |
| Diabetes=0 | District0 | 456 | 13758.8 (7012.9-26098.5) |  | 406 | 12227.1 (6555.9-20013.4) | 0.106 |
|  | District1 | 825 | 14105.3 (8125.1-25445.3) |  | 896 | 14180.3 (8487.9-24120.2) | 0.455 |
| Diabetes=1 | District0 | 53 | 15065.0 (8758.3-23940.7) |  | 46 | 11969.3 (6010.4-29735.9) | 0.473 |
|  | District1 | 144 | 14795.8 (7725.2-26533.0) |  | 130 | 14239.0 (7910.2-31949.3) | 0.259 |
| Diabetes=0 | Education1 | 110 | 15285.4 (7476.0-49108.5) |  | 104 | 13936.4 (6911.1-29582.5) | 0.081 |
|  | Education2 | 428 | 13956.7 (7298.4-25621.4) |  | 388 | 13787.9 (7848.7-21848.6) | 0.965 |
|  | Education3 | 380 | 14149.9 (7774.2-23662.5) |  | 439 | 13108.7 (7362.8-23002.3) | 0.120 |
|  | Education4 | 189 | 15049.5 (8683.0-22811.6) |  | 201 | 15352.1 (8980.9-22941.7) | 0.983 |
|  | Education5 | 174 | 12748.3 (8670.4-24746.4) |  | 170 | 13549.7 (8354.9-22133.2) | 0.994 |
| Diabetes=1 | Education1 | 21 | 13454.9 (8954.2-48232.3) |  | 10 | 7122.5 (5051.0-9258.7) | 0.011 |
|  | Education2 | 81 | 15326.6 (10345.2-22904.2) |  | 67 | 13813.6 (6850.0-46688.6) | 0.966 |
|  | Education3 | 46 | 12839.8 (7057.9-18678.2) |  | 43 | 10637.2 (7330.7-24235.0) | 0.377 |
|  | Education4 | 30 | 13899.1 (6197.4-23711.6) |  | 33 | 16986.6 (10633.5-27643.3) | 0.327 |
|  | Education5 | 19 | 24311.9 (13405.6-32309.6) |  | 23 | 16517.0 (11264.3-35297.1) | 0.858 |
| Smoking=0 | Drinking0 | 771 | 13788.4 (7000.9-26049.4) |  | 790 | 13284.7 (7686.7-22085.2) | 0.357 |
|  | Drinking1 | 129 | 15599.6 (12031.2-37669.7) |  | 133 | 16634.8 (9247.5-30544.6) | 0.078 |
| Smoking=1 | Drinking0 | 146 | 13909.6 (7325.6-19825.6) |  | 139 | 15143.3 (7350.5-31268.4) | 0.325 |
|  | Drinking1 | 432 | 14718.2 (8309.5-25690.9) |  | 416 | 13194.9 (8071.5-23257.0) | 0.764 |
| Smoking=0 | District0 | 301 | 14196.5 (7484.7-29903.8) |  | 269 | 12455.3 (6926.6-22078.8) | 0.061 |
|  | District1 | 599 | 14052.8 (7531.2-26230.0) |  | 654 | 14121.4 (8211.5-23599.8) | 0.382 |
| Smoking=1 | District0 | 208 | 12234.2 (6818.8-23374.0) |  | 183 | 11526.1 (6253.6-20268.7) | 0.456 |
|  | District1 | 370 | 14826.6 (9302.5-24415.4) |  | 372 | 14553.9 (8696.7-27734.1) | 0.530 |
| Smoking=0 | Education1 | 105 | 15210.3 (8166.0-45712.0) |  | 93 | 13924.8 (7049.3-31469.7) | 0.107 |
|  | Education2 | 276 | 14364.1 (6912.1-27486.1) |  | 276 | 14472.0 (7589.0-22881.9) | 0.876 |
|  | Education3 | 233 | 13864.3 (7520.5-21902.8) |  | 272 | 12505.4 (7392.7-19619.2) | 0.123 |
|  | Education4 | 134 | 13997.4 (7936.4-21894.2) |  | 144 | 14896.5 (9140.2-22597.5) | 0.909 |
|  | Education5 | 152 | 13518.7 (8417.1-28959.6) |  | 138 | 13942.5 (8415.1-25012.9) | 0.728 |
| Smoking=1 | Education1 | 26 | 22418.4 (8099.1-59620.1) |  | 21 | 9541.8 (6023.3-16923.3) | 0.042 |
|  | Education2 | 233 | 14162.6 (8631.0-22132.4) |  | 179 | 12664.2 (8134.5-21719.7) | 0.479 |
|  | Education3 | 193 | 14371.9 (7886.2-23428.0) |  | 210 | 14259.4 (7172.1-27612.2) | 0.944 |
|  | Education4 | 85 | 15531.3 (7358-24449.9) |  | 90 | 16147.0 (10041.9-28797.7) | 0.599 |
|  | Education5 | 41 | 12069.5 (9592.3-21146.8) |  | 55 | 12455.2 (8225.3-20210.9) | 0.329 |
| Drinking=0 | District0 | 307 | 13355.6 (6830.7-25895.7) |  | 292 | 12282.8 (6153.2-21062.1) | 0.299 |
|  | District1 | 610 | 13959.8 (7239.8-22342.0) |  | 637 | 13876.9 (8207.2-23786.0) | 0.933 |
| Drinking=1 | District0 | 202 | 14544.2 (8065.5-27745.6) |  | 160 | 12226.5 (6977.4-20950.8) | 0.102 |
|  | District1 | 359 | 14934.4 (10156.6-28114.8) |  | 389 | 15026.2 (8878.8-26339.7) | 0.602 |
| Drinking=0 | Education1 | 102 | 15227.2 (7547.0-47044.3) |  | 87 | 12995.3 (6996.0-29379.5) | 0.048 |
|  | Education2 | 291 | 13947.6 (6514.5-23471.1) |  | 278 | 14254.2 (7558.1-25504.9) | 0.090 |
|  | Education3 | 248 | 13456.4 (7070.1-20381.8) |  | 272 | 12451.2 (6953.7-19851.7) | 0.350 |
|  | Education4 | 136 | 13963.8 (7250.1-21760.4) |  | 154 | 14868.7 (8962.1-21993.1) | 0.578 |
|  | Education5 | 140 | 13637.3 (8915.1-27665.6) |  | 138 | 13966.8 (8354.9-23574.6) | 0.698 |
| Drinking=1 | Education1 | 29 | 15003.9 (9665.5-51336.8) |  | 27 | 15607.0 (6525.0-22275.2) | 0.073 |
|  | Education2 | 218 | 15136.2 (9913.4-25553.0) |  | 177 | 12986.3 (8138.8-21107.3) | 0.378 |
|  | Education3 | 178 | 15665.4 (8322.6-33115.6) |  | 210 | 14259.4 (7547.6-27931.5) | 0.314 |
|  | Education4 | 83 | 15364.8 (10992.1-26615.5) |  | 80 | 17091.7 (11341.4-30809.0) | 0.989 |
|  | Education5 | 53 | 12056.0 (9389.7-24158.6) |  | 55 | 12261.3 (8818.0-21523.8) | 0.379 |
| District=0 | Education1 | 58 | 14634.0 (7373.5-52289.1) |  | 55 | 10195.0 (6710.0-23552.0) | 0.081 |
|  | Education2 | 228 | 13900.6 (7232.2-25621.4) |  | 167 | 12664.0 (7081.0-20525.0) | 0.327 |
|  | Education3 | 169 | 14194.5 (7901.0-24441.4) |  | 164 | 11100.0 (6050.0-18678.0) | 0.145 |
|  | Education4 | 34 | 15361.5 (6292.2-23214.6) |  | 54 | 15307.0 (6658.0-22082.0) | 0.502 |
|  | Education5 | 20 | 9632.3 (5573.7-23995.2) |  | 12 | 21505.0 (16921.0-31565.0) | 0.110 |
| District=1 | Education1 | 73 | 15326.8 (9475.1-47218.8) |  | 59 | 13948.0 (7000.0-31505.0) | 0.137 |
|  | Education2 | 281 | 14434.7 (7906.6-24874.0) |  | 288 | 14377.0 (8238.0-25775.0) | 0.304 |
|  | Education3 | 257 | 14086.1 (7009.1-21504.0) |  | 318 | 14054.0 (8115.0-25391.0) | 0.543 |
|  | Education4 | 185 | 14700.7 (7687.6-22260.6) |  | 180 | 15726.0 (10639.0-25906.0) | 0.780 |
|  | Education5 | 173 | 13578.8 (9517.5-27057.7) |  | 181 | 13315.0 (8207.0-22588.0) | 0.686 |
| Definition of subgroup labels: Sex: 1 [Male], 2 [Female]; Age: 1 [<51years], 2 [51-59years], 3 [60-67years], 4 [≥68years]; BMI 1 [<18.5], 2 [18.5-24], 3 [24-28], 4 [≥28]; Length of Stay (LOS): 1 [1-6 days], 2 [7-13 days], 3 [14-29 days], 4 [≥30 days]; Surgery: 0 [No], 1 [Yes]; Diagnosis: 1 [Benign tumor], 2 [Malignant tumor], 3 [Inflammation], 4 [Hernia], 5 [Intestinal obstruction], 6 [Others]; Hypertension: 0 [No], 1 [Yes]; Diabetes: 0 [No], 1 [Yes]; Smoking: 0 [No], 1 [Yes]; Drinking: 0 [No], 1 [Yes]; District: 0 [Rural], 1 [Urban]; Education: 1 [Illiterate], 2 [Primary school], 3 [Middle school], 4 [High school], 5 [University degree]; | | | | | | | |
| *p* values are determined by T-test or Wilcoxon Rank Sum test. | | | |  |  |  |  |

| Supplemental Table 3 | Length of Stay (LOS) of patients by light sides of the hospital units across 616 plural subgroups. | | | | | | |
| --- | --- | --- | --- | --- | --- | --- | --- |
| **Subgroups** | | **Low Light Side** | |  | **High Light Side** | | ***p*-value** |
|  |  | **N** | **median (Q1-Q3)** | | **N** | **median (Q1-Q3)** |  |
| Sex=1 | Age1 | 170 | 6 (3-10) |  | 180 | 6 (3-11.3) | 0.369 |
|  | Age2 | 194 | 6 (3-12) |  | 202 | 6 (3-10.8) | 0.406 |
|  | Age3 | 257 | 5 (2-10) |  | 262 | 5 (2-11.8) | 0.220 |
|  | Age4 | 268 | 7 (3-14) |  | 229 | 7 (3-15) | 0.392 |
| Sex=2 | Age1 | 171 | 6 (4-11) |  | 178 | 6 (3-10) | 0.092 |
|  | Age2 | 164 | 7 (4-13) |  | 159 | 6 (3-11) | 0.158 |
|  | Age3 | 108 | 6 (4-10.3) |  | 114 | 7 (3.3-14) | 0.114 |
|  | Age4 | 146 | 9.5 (5-19) |  | 154 | 9 (5-15) | 0.123 |
| Sex=1 | BMI1 | 92 | 4 (2-10) |  | 109 | 6 (2-12) | 0.371 |
|  | BMI2 | 608 | 6 (2-12) |  | 561 | 6 (3-12) | 0.928 |
|  | BMI3 | 162 | 7 (4-11) |  | 168 | 7 (4-14) | 0.286 |
|  | BMI4 | 27 | 7 (4.5-14) |  | 35 | 7 (4-15.5) | 0.526 |
| Sex=2 | BMI1 | 116 | 6 (3-13.5) |  | 130 | 5 (2-10) | 0.031 |
|  | BMI2 | 359 | 7 (4-14) |  | 357 | 7 (4-12) | 0.495 |
|  | BMI3 | 93 | 7 (4-13) |  | 95 | 7 (4-12) | 0.884 |
|  | BMI4 | 21 | 9 (7-14) |  | 23 | 7 (6-11.5) | 0.392 |
| Sex=1 | LOS1 | 480 | 3 (2-4) |  | 455 | 3 (2-4) | 0.340 |
|  | LOS2 | 223 | 9 (7.5-11) |  | 220 | 9 (7-11) | 0.239 |
|  | LOS3 | 147 | 18 (15-22) | | 161 | 18 (15-22) | 0.958 |
|  | LOS4 | 39 | 36 (31.5-42.5) | | 37 | 37 (33-45) | 0.160 |
| Sex=2 | LOS1 | 273 | 4 (2-5) |  | 298 | 3 (2-5) | 0.457 |
|  | LOS2 | 163 | 9 (7-11) |  | 185 | 9 (7-11) | 0.384 |
|  | LOS3 | 114 | 18 (15-21) | | 100 | 19 (15-23) | 0.280 |
|  | LOS4 | 39 | 36 (33-45.5) | | 22 | 42 (35.3-52) | 0.466 |
| Sex=1 | Surgery0 | 182 | 6 (3-10) |  | 185 | 6 (3-9) | 0.254 |
|  | Surgery1 | 707 | 6 (2.5-13) |  | 688 | 7 (3-13.3) | 0.133 |
| Sex=2 | Surgery0 | 87 | 7 (4-12.5) |  | 99 | 6 (3-10) | 0.087 |
|  | Surgery1 | 502 | 7 (4-14) |  | 506 | 7 (4-12) | 0.211 |
| Sex=1 | Diagnosis1 | 624 | 4 (2-10) |  | 623 | 5 (2-11) | 0.151 |
|  | Diagnosis2 | 40 | 8 (6-12) |  | 38 | 8.5 (6-14.8) | 0.280 |
|  | Diagnosis3 | 117 | 9 (6-14) |  | 107 | 7 (5-13) | 0.160 |
|  | Diagnosis4 | 20 | 7 (5-9.3) |  | 20 | 7 (6-10) | 0.506 |
|  | Diagnosis5 | 31 | 7 (6-10) |  | 27 | 7 (6-12) | 0.701 |
|  | Diagnosis6 | 57 | 10 (7-18) |  | 58 | 9 (6-15) | 0.635 |
| Sex=2 | Diagnosis1 | 331 | 6 (3-14) |  | 353 | 5 (2-11) | 0.100 |
|  | Diagnosis2 | 59 | 8 (5.5-13) |  | 60 | 7 (4-13.3) | 0.988 |
|  | Diagnosis3 | 127 | 7 (5-12) |  | 132 | 8 (5-12) | 0.226 |
|  | Diagnosis4 | 10 | 7.5 (7-10.8) | | 5 | 8 (8-9) | 0.585 |
|  | Diagnosis5 | 31 | 11 (7-21) |  | 28 | 10 (7-16.5) | 0.239 |
|  | Diagnosis6 | 31 | 9 (6.5-17) |  | 27 | 8 (6-15) | 0.412 |
| Sex=1 | Hypertension0 | 584 | 6 (3-12) |  | 563 | 6 (3-12) | 0.573 |
|  | Hypertension1 | 305 | 5 (2-11) |  | 310 | 7 (3-14) | 0.386 |
| Sex=2 | Hypertension0 | 423 | 6 (4-12) |  | 441 | 6 (3-11) | 0.272 |
|  | Hypertension1 | 166 | 8 (5-17) |  | 164 | 9 (5-14) | 0.101 |
| Sex=1 | Diabetes0 | 752 | 6 (3-12) |  | 756 | 6 (3-12.3) | 0.648 |
|  | Diabetes1 | 137 | 4 (2-10) |  | 117 | 6 (3-13) | 0.136 |
| Sex=2 | Diabetes0 | 529 | 7 (4-14) |  | 546 | 6 (3-11) | 0.044 |
|  | Diabetes1 | 60 | 7.5 (4-14) |  | 59 | 9 (5-14) | 0.772 |
| Sex=1 | Smoking0 | 320 | 7 (3-12) |  | 322 | 7 (4-13) | 0.467 |
|  | Smoking1 | 569 | 5 (2-12) |  | 551 | 6 (2-12) | 0.498 |
| Sex=2 | Smoking0 | 580 | 7 (4-14) |  | 601 | 7 (4-11) | 0.063 |
|  | Smoking1 | 9 | 7 (5-8) |  | 4 | 11 (6.5-14.5) | 0.927 |
| Sex=1 | Drinking0 | 368 | 6 (3-11) |  | 371 | 7 (3-13) | 0.018 |
|  | Drinking1 | 521 | 6 (2-13) |  | 502 | 6 (2-12) | 0.577 |
| Sex=2 | Drinking0 | 549 | 7 (4-14) |  | 558 | 6 (3-11) | 0.160 |
|  | Drinking1 | 40 | 8 (5-17.3) |  | 47 | 8 (4-15) | 0.148 |
| Sex=1 | District0 | 305 | 5 (2-11) |  | 256 | 6 (3-10.3) | 0.719 |
|  | District1 | 584 | 6 (3-12) |  | 617 | 6 (3-13) | 0.230 |
| Sex=2 | District0 | 204 | 7 (4-15) |  | 196 | 6 (3-11) | 0.017 |
|  | District1 | 385 | 7 (4-13) |  | 409 | 7 (4-12) | 0.456 |
| Sex=1 | Education1 | 41 | 11 (4-20) |  | 36 | 6 (3-10.8) | 0.082 |
|  | Education2 | 319 | 5 (2-11) |  | 254 | 6 (2.3-13) | 0.063 |
|  | Education3 | 278 | 6 (3-11) |  | 314 | 5.5 (2-11) | 0.352 |
|  | Education4 | 135 | 6 (3-12) |  | 148 | 7 (4-13) | 0.227 |
|  | Education5 | 116 | 7 (3.8-12) |  | 121 | 7 (3-14) | 0.412 |
| Sex=2 | Education1 | 90 | 9.5 (5-18) |  | 78 | 7 (4-14) | 0.032 |
|  | Education2 | 190 | 7 (4-14) |  | 201 | 7 (4-13) | 0.796 |
|  | Education3 | 148 | 6 (3-10) |  | 168 | 6 (3-10) | 0.396 |
|  | Education4 | 84 | 6 (4-14) |  | 86 | 5 (3-11) | 0.137 |
|  | Education5 | 77 | 7 (5-13) |  | 72 | 7 (5-11) | 0.876 |
| Age=1 | BMI1 | 48 | 4.5 (2-7) |  | 67 | 5 (3-12) | 0.905 |
|  | BMI2 | 207 | 6 (3-10) |  | 223 | 6 (3-9) | 0.520 |
|  | BMI3 | 72 | 8 (5-11) |  | 51 | 6 (4-11.5) | 0.379 |
|  | BMI4 | 14 | 7.5 (4.3-12.8) | | 17 | 5 (2-9) | 0.535 |
| Age=2 | BMI1 | 55 | 4 (2-13) |  | 53 | 3 (2-7) | 0.205 |
|  | BMI2 | 221 | 7 (3-13) |  | 214 | 6 (3-10) | 0.173 |
|  | BMI3 | 62 | 6 (4-10.8) |  | 76 | 7 (3-11.3) | 0.473 |
|  | BMI4 | 20 | 7 (5.8-14.3) | | 18 | 7 (6-14.8) | 0.900 |
| Age=3 | BMI1 | 41 | 5 (3-11) |  | 59 | 4 (2-8.5) | 0.774 |
|  | BMI2 | 259 | 5 (2-10) |  | 225 | 5 (2-12) | 0.241 |
|  | BMI3 | 60 | 7 (3-10) |  | 75 | 7 (4-14) | 0.112 |
|  | BMI4 | 5 | 18 (17-19) | | 17 | 10 (6-15) | 0.468 |
| Age=4 | BMI1 | 64 | 6 (4-13.5) |  | 60 | 6 (3-12) | 0.399 |
|  | BMI2 | 280 | 7 (3-16.3) |  | 256 | 8 (4-15) | 0.822 |
|  | BMI3 | 61 | 9 (3-18) |  | 61 | 9 (6-16) | 0.839 |
|  | BMI4 | 9 | 11 (6-13) |  | 6 | 12 (7.8-15.5) | 0.371 |
| Age=1 | LOS1 | 180 | 4 (2-5) |  | 198 | 3 (2-5) | 0.524 |
|  | LOS2 | 99 | 9 (7-10.5) |  | 101 | 9 (7-11) | 0.845 |
|  | LOS3 | 47 | 16 (14-19) | | 47 | 18 (15-21) | 0.082 |
|  | LOS4 | 15 | 37 (33-41.5) | | 12 | 36 (33.8-38) | 0.321 |
| Age=2 | LOS1 | 176 | 3 (2-4) |  | 201 | 3 (2-4) | 0.457 |
|  | LOS2 | 103 | 9 (7-10) |  | 98 | 9 (7-11) | 0.957 |
|  | LOS3 | 59 | 17 (15-21) | | 52 | 17 (15-20) | 0.472 |
|  | LOS4 | 20 | 36 (32.8-42) | | 10 | 40.5 (34.8-58.5) | 0.200 |
| Age=3 | LOS1 | 212 | 3 (2-5) |  | 198 | 3 (2-4) | 0.994 |
|  | LOS2 | 86 | 9 (8-11) |  | 90 | 8 (7-10) | 0.174 |
|  | LOS3 | 54 | 18.5 (15.3-21) | | 70 | 19 (15-22) | 0.892 |
|  | LOS4 | 13 | 35 (31-44) | | 18 | 42 (35.5-51) | 0.137 |
| Age=4 | LOS1 | 185 | 3 (2-5) |  | 156 | 3 (2-5) | 0.581 |
|  | LOS2 | 98 | 9 (7-11) |  | 116 | 9 (8-11) | 0.862 |
|  | LOS3 | 101 | 18 (16-23) | | 92 | 20 (16-22) | 0.553 |
|  | LOS4 | 30 | 36 (34.3-48.3) | | 19 | 41 (32-49) | 0.846 |
| Age=1 | Surgery0 | 56 | 5.5 (3-10) |  | 58 | 5.5 (3-10) | 0.361 |
|  | Surgery1 | 285 | 6 (4-11) |  | 300 | 6 (3-11) | 0.678 |
| Age=2 | Surgery0 | 56 | 7 (4-10) |  | 65 | 5 (3-8) | 0.188 |
|  | Surgery1 | 302 | 7 (3-13) |  | 296 | 6 (3-11) | 0.245 |
| Age=3 | Surgery0 | 61 | 5 (2-9) |  | 75 | 5 (2-8) | 0.429 |
|  | Surgery1 | 304 | 5 (2-11) |  | 301 | 6 (3-14) | 0.065 |
| Age=4 | Surgery0 | 96 | 7 (3-13) |  | 86 | 6.5 (4.3-10.8) | 0.842 |
|  | Surgery1 | 318 | 8 (4-17) |  | 297 | 9 (4-16) | 0.812 |
| Age=1 | Diagnosis1 | 165 | 5 (2-9) |  | 201 | 5 (2-10) | 0.558 |
|  | Diagnosis2 | 34 | 8 (4.3-11.8) | | 24 | 7 (4-11.3) | 0.643 |
|  | Diagnosis3 | 100 | 7 (5-11) |  | 77 | 6 (4-9) | 0.733 |
|  | Diagnosis4 | 5 | 5 (5-6) |  | 6 | 7 (5.3-10.3) | 0.284 |
|  | Diagnosis5 | 14 | 9.5 (6-19.3) | | 17 | 10 (7-15) | 0.760 |
|  | Diagnosis6 | 23 | 10 (6-15.5) | | 33 | 7 (6-13) | 0.410 |
| Age=2 | Diagnosis1 | 246 | 5 (2-10) |  | 262 | 5 (3-9) | 0.249 |
|  | Diagnosis2 | 27 | 7 (5.5-10.5) | | 24 | 6 (4-11) | 0.467 |
|  | Diagnosis3 | 44 | 8.5 (6-13.3) | | 45 | 8 (5-14) | 0.740 |
|  | Diagnosis4 | 7 | 7 (5-8.5) |  | 2 | 8.5 (7.8-9.3) | NA |
|  | Diagnosis5 | 18 | 9.5 (7-22) |  | 12 | 9 (7-12.3) | 0.069 |
|  | Diagnosis6 | 16 | 18 (8.8-23) | | 16 | 8.5 (5.8-13.5) | 0.035 |
| Age=3 | Diagnosis1 | 276 | 4 (2-10) |  | 277 | 5 (2-10) | 0.086 |
|  | Diagnosis2 | 16 | 9 (7-12.3) |  | 20 | 11 (7-15) | 0.868 |
|  | Diagnosis3 | 38 | 7 (5-11.8) |  | 49 | 9 (5-13) | 0.917 |
|  | Diagnosis4 | 7 | 8 (7-10.5) |  | 5 | 7 (7-7) | 0.158 |
|  | Diagnosis5 | 7 | 9 (8-17) |  | 10 | 9.5 (6.3-19) | 0.928 |
|  | Diagnosis6 | 21 | 9 (7-10) |  | 15 | 8 (7-16.5) | 0.358 |
| Age=4 | Diagnosis1 | 268 | 6 (2-17) |  | 236 | 7 (3-15) | 0.465 |
|  | Diagnosis2 | 22 | 8.5 (6-14) |  | 30 | 7 (6-15.5) | 0.738 |
|  | Diagnosis3 | 62 | 11 (6-16) |  | 68 | 9 (7-13.3) | 0.710 |
|  | Diagnosis4 | 11 | 9 (7-11) |  | 12 | 8.5 (6-10.5) | 0.670 |
|  | Diagnosis5 | 23 | 7 (6-16) |  | 16 | 7 (5.8-21.5) | 0.551 |
|  | Diagnosis6 | 28 | 9.5 (6-17.3) | | 21 | 11 (8-19) | 0.535 |
| Age=1 | Hypertension0 | 314 | 6 (3-11) |  | 332 | 6 (3-10) | 0.361 |
|  | Hypertension1 | 27 | 7 (3.5-10.5) | | 26 | 5.5 (2-12) | 0.872 |
| Age=2 | Hypertension0 | 257 | 6 (3-13) |  | 268 | 6 (3-11) | 0.650 |
|  | Hypertension1 | 101 | 7 (3-10) |  | 93 | 6 (3-8) | 0.153 |
| Age=3 | Hypertension0 | 232 | 5 (2-11) |  | 225 | 6 (3-12) | 0.138 |
|  | Hypertension1 | 133 | 5 (2-10) |  | 151 | 6 (2-14) | 0.286 |
| Age=4 | Hypertension0 | 204 | 7 (3.8-16.3) | | 179 | 7 (3-13) | 0.589 |
|  | Hypertension1 | 210 | 7 (4-16) |  | 204 | 9.5 (5-16) | 0.877 |
| Age=1 | Diabetes0 | 328 | 6 (3-10.3) |  | 350 | 6 (3-10) | 0.355 |
|  | Diabetes1 | 13 | 8 (4-13) |  | 8 | 5.5 (4.5-19.8) | 0.583 |
| Age=2 | Diabetes0 | 307 | 7 (3-13) |  | 307 | 6 (3-11) | 0.138 |
|  | Diabetes1 | 51 | 4 (3-7.5) |  | 54 | 6 (2-8) | 0.706 |
| Age=3 | Diabetes0 | 308 | 5 (2-11) |  | 331 | 6 (3-13) | 0.146 |
|  | Diabetes1 | 57 | 5 (2-10) |  | 45 | 4 (2-19) | 0.199 |
| Age=4 | Diabetes0 | 338 | 7 (4-17) |  | 314 | 8 (4-15) | 0.346 |
|  | Diabetes1 | 76 | 6.5 (3-15) |  | 69 | 9 (5-16) | 0.229 |
| Age=1 | Smoking0 | 250 | 6 (4-11) |  | 266 | 6 (3-10) | 0.097 |
|  | Smoking1 | 91 | 5 (3-9) |  | 92 | 6 (3-11.3) | 0.149 |
| Age=2 | Smoking0 | 228 | 7 (4-12.3) |  | 218 | 6 (3-11) | 0.131 |
|  | Smoking1 | 130 | 6 (2.3-12) |  | 143 | 5 (3-10.5) | 0.554 |
| Age=3 | Smoking0 | 178 | 6 (3-11) |  | 191 | 7 (3-13) | 0.246 |
|  | Smoking1 | 187 | 5 (2-10) |  | 185 | 5 (2-11) | 0.421 |
| Age=4 | Smoking0 | 244 | 8 (4-18) |  | 248 | 9 (5-15) | 0.417 |
|  | Smoking1 | 170 | 6.5 (2.3-14) | | 135 | 7 (3-15.5) | 0.882 |
| Age=1 | Drinking0 | 242 | 6 (4-11) |  | 266 | 6 (3-10) | 0.218 |
|  | Drinking1 | 99 | 6 (3-9.5) |  | 92 | 7 (2.8-12.3) | 0.611 |
| Age=2 | Drinking0 | 230 | 6 (3-12) |  | 222 | 6 (3-11) | 0.422 |
|  | Drinking1 | 128 | 7 (3-13) |  | 139 | 6 (3-10.5) | 0.118 |
| Age=3 | Drinking0 | 196 | 6 (3-10.3) |  | 192 | 7 (3-13.3) | 0.123 |
|  | Drinking1 | 169 | 5 (2-10) |  | 184 | 5 (2-11.3) | 0.269 |
| Age=4 | Drinking0 | 249 | 7 (4-16) |  | 249 | 9 (5-16) | 0.346 |
|  | Drinking1 | 165 | 7 (3-16) |  | 134 | 6.5 (3-14) | 0.058 |
| Age=1 | District0 | 100 | 6 (4-10.3) |  | 120 | 6 (3-10) | 0.247 |
|  | District1 | 241 | 6 (3-11) |  | 238 | 6 (3-11) | 0.745 |
| Age=2 | District0 | 119 | 7 (3-14) |  | 127 | 5 (3-9) | 0.004 |
|  | District1 | 239 | 6 (3-10.5) |  | 234 | 6 (3-11) | 0.883 |
| Age=3 | District0 | 157 | 5 (2-9) |  | 110 | 4 (2-11) | 0.383 |
|  | District1 | 208 | 6 (2-12) |  | 266 | 6 (3-13) | 0.226 |
| Age=4 | District0 | 133 | 8 (4-16) |  | 95 | 8 (4-16) | 0.665 |
|  | District1 | 281 | 7 (3-16) |  | 288 | 8 (4-15) | 0.733 |
| Age=1 | Education1 | 2 | 22 (14-30) | | 4 | 12 (4.3-21) | NA |
|  | Education2 | 40 | 7 (4-13) |  | 49 | 6 (3-11) | 0.336 |
|  | Education3 | 118 | 6 (3-9) |  | 139 | 5 (3-9) | 0.951 |
|  | Education4 | 60 | 6 (3-13.3) |  | 75 | 7 (4-11.5) | 0.458 |
|  | Education5 | 121 | 7 (4-11) |  | 91 | 6 (4-11.5) | 0.975 |
| Age=2 | Education1 | 23 | 7 (4-11.5) |  | 17 | 3 (3-6) | 0.115 |
|  | Education2 | 100 | 6 (3-12) |  | 97 | 6 (3-10) | 0.865 |
|  | Education3 | 127 | 7 (3-13) |  | 137 | 6 (3-11) | 0.201 |
|  | Education4 | 78 | 7 (3-13) |  | 77 | 6 (4-11) | 0.456 |
|  | Education5 | 30 | 7 (3.3-11.8) | | 33 | 5 (2-9) | 0.227 |
| Age=3 | Education1 | 45 | 7 (4-14) |  | 34 | 7 (3.3-13) | 0.725 |
|  | Education2 | 172 | 5 (2-10.3) |  | 159 | 6 (3-13) | 0.055 |
|  | Education3 | 98 | 5 (2-9.8) |  | 117 | 5 (2-11) | 0.762 |
|  | Education4 | 36 | 5.5 (2-8) |  | 39 | 6 (4-12.5) | 0.375 |
|  | Education5 | 14 | 5.5 (4-14.8) | | 27 | 7 (4.5-13.5) | 0.800 |
| Age=4 | Education1 | 61 | 15 (7-21) |  | 59 | 8 (5.5-14) | 0.003 |
|  | Education2 | 197 | 6 (2-14) |  | 150 | 8 (4-16) | 0.459 |
|  | Education3 | 83 | 7 (3-13.5) |  | 89 | 7 (3-13) | 0.246 |
|  | Education4 | 45 | 6 (4-14) |  | 43 | 9 (3-14) | 0.520 |
|  | Education5 | 28 | 9 (6-17.3) |  | 42 | 10.5 (6-20.5) | 0.402 |
| BMI=1 | LOS1 | 124 | 3 (2-5) |  | 143 | 3 (1-4) | 0.382 |
|  | LOS2 | 38 | 8.5 (7-11) |  | 49 | 9 (8-11) | 0.838 |
|  | LOS3 | 30 | 19 (17-21.8) | | 32 | 18.5 (14-22.3) | 0.441 |
|  | LOS4 | 16 | 43 (36.8-57) | | 15 | 38 (31.5-43) | 0.217 |
| BMI=2 | LOS1 | 501 | 3 (2-5) |  | 476 | 3 (2-5) | 0.227 |
|  | LOS2 | 240 | 9 (7-11) |  | 253 | 9 (7-11) | 0.883 |
|  | LOS3 | 174 | 18 (15-21) | | 156 | 19 (16-22.3) | 0.113 |
|  | LOS4 | 52 | 35 (31.8-42.3) | | 33 | 39 (34-52) | 0.075 |
| BMI=3 | LOS1 | 112 | 3 (2-5) |  | 111 | 3 (2-5) | 0.930 |
|  | LOS2 | 91 | 9 (8-11) |  | 86 | 9 (7-11) | 0.680 |
|  | LOS3 | 44 | 18 (15-22.3) | | 59 | 18 (15-21) | 0.465 |
|  | LOS4 | 8 | 35 (33-38.5) | | 7 | 43 (35-71.5) | 0.201 |
| BMI=4 | LOS1 | 16 | 4.5 (2.8-6) | | 23 | 4 (1.5-5.5) | 0.220 |
|  | LOS2 | 17 | 9 (7-11) |  | 17 | 8 (7-10) | 0.564 |
|  | LOS3 | 13 | 17 (14-18) | | 14 | 16 (15-19.3) | 0.905 |
|  | LOS4 | 2 | 40 (35-45) | | 4 | 38 (34-47) | NA |
| BMI=1 | Surgery0 | 175 | 5 (2-10) |  | 193 | 5 (2-11) | 0.820 |
|  | Surgery1 | 33 | 8 (4-17) |  | 46 | 6 (3-10) | 0.041 |
| BMI=2 | Surgery0 | 789 | 6 (3-14) |  | 729 | 7 (3-13) | 0.925 |
|  | Surgery1 | 178 | 6 (2.3-10) |  | 189 | 6 (3-8) | 0.193 |
| BMI=3 | Surgery0 | 205 | 8 (4-13) |  | 222 | 7 (4-14) | 0.516 |
|  | Surgery1 | 50 | 5.5 (3-10) |  | 41 | 7 (3-9) | 0.508 |
| BMI=4 | Surgery0 | 40 | 9 (6-15.3) |  | 50 | 7 (4.3-15.8) | 0.977 |
|  | Surgery1 | 8 | 7 (6.5-12.3) | | 8 | 8 (4-10.8) | 0.901 |
| BMI=1 | Diagnosis1 | 162 | 4 (2-7) |  | 180 | 4 (2-9.3) | 0.872 |
|  | Diagnosis2 | 4 | 5.5 (3.8-11) | | 6 | 11 (7-13.5) | 0.915 |
|  | Diagnosis3 | 13 | 10 (7-19) |  | 20 | 5.5 (3.8-8.5) | 0.126 |
|  | Diagnosis4 | 1 | 8 (8-8) |  | 0 | NA | NA |
|  | Diagnosis5 | 19 | 17 (10.5-24) | | 20 | 9.5 (6.8-14.5) | 0.112 |
|  | Diagnosis6 | 10 | 10 (6.3-18) | | 12 | 7 (3.8-16) | 0.683 |
| BMI=2 | Diagnosis1 | 624 | 5 (2-12) |  | 605 | 5 (2-11) | 0.555 |
|  | Diagnosis2 | 68 | 8 (5-12.3) |  | 68 | 7 (5-15) | 0.517 |
|  | Diagnosis3 | 163 | 7 (5-13) |  | 144 | 7 (5.8-12) | 0.778 |
|  | Diagnosis4 | 18 | 7 (5-10.8) |  | 17 | 8 (6-11) | 0.321 |
|  | Diagnosis5 | 36 | 7 (6-10) |  | 33 | 7 (6-13) | 0.663 |
|  | Diagnosis6 | 58 | 12 (8-18.8) | | 51 | 9 (6-14.5) | 0.175 |
| BMI=3 | Diagnosis1 | 143 | 6 (2-13) |  | 155 | 7 (3-12.5) | 0.845 |
|  | Diagnosis2 | 23 | 8 (6.5-11) |  | 18 | 7.5 (4.5-9) | 0.894 |
|  | Diagnosis3 | 54 | 7.5 (6-11) |  | 61 | 10 (6-14) | 0.112 |
|  | Diagnosis4 | 11 | 7 (7-9) |  | 7 | 7 (5.5-7.5) | 0.201 |
|  | Diagnosis5 | 7 | 7 (5-17) |  | 2 | 29 (25-33) | 0.250 |
|  | Diagnosis6 | 17 | 9 (6-10) |  | 20 | 8.5 (6-17) | 0.223 |
| BMI=4 | Diagnosis1 | 26 | 7 (5-14.8) |  | 36 | 7 (2-16.3) | 0.820 |
|  | Diagnosis2 | 4 | 7.5 (6.8-10) | | 6 | 13 (8.3-15.5) | 0.486 |
|  | Diagnosis3 | 14 | 10.5 (8.3-14) | | 14 | 7 (6-9.8) | 0.043 |
|  | Diagnosis4 | 0 | NA |  | 0 | NA | NA |
|  | Diagnosis5 | 0 | NA |  | 1 | 2 (2-2) | NA |
|  | Diagnosis6 | 3 | 6 (5-9.5) |  | 2 | 15 (15-15) | 0.115 |
| BMI=1 | Hypertension0 | 170 | 5 (2-12) |  | 187 | 5 (2-10) | 0.576 |
|  | Hypertension1 | 38 | 7 (3.3-14) |  | 52 | 5.5 (2-12) | 0.211 |
| BMI=2 | Hypertension0 | 675 | 6 (3-12) |  | 645 | 6 (3-11) | 0.769 |
|  | Hypertension1 | 292 | 6 (3-14) |  | 273 | 7 (3-13) | 0.687 |
| BMI=3 | Hypertension0 | 138 | 8 (4-12) |  | 142 | 7 (4-12) | 0.936 |
|  | Hypertension1 | 117 | 7 (3-12) |  | 121 | 7 (4-14) | 0.190 |
| BMI=4 | Hypertension0 | 24 | 7 (4.8-11.3) | | 30 | 7 (4.3-14.8) | 0.284 |
|  | Hypertension1 | 24 | 12 (6-17.3) | | 28 | 8 (4-14.3) | 0.546 |
| BMI=1 | Diabetes0 | 189 | 5 (2-12) |  | 220 | 5 (2-10) | 0.160 |
|  | Diabetes1 | 19 | 3 (2-12) |  | 19 | 8 (3-17) | 0.361 |
| BMI=2 | Diabetes0 | 837 | 6 (3-13) |  | 811 | 6 (3-11) | 0.283 |
|  | Diabetes1 | 130 | 5 (2-10) |  | 107 | 7 (3-13) | 0.133 |
| BMI=3 | Diabetes0 | 214 | 7 (4-11.8) |  | 222 | 7 (4-13.8) | 0.311 |
|  | Diabetes1 | 41 | 8 (3-13) |  | 41 | 8 (4-13) | 0.967 |
| BMI=4 | Diabetes0 | 41 | 9 (6-14) |  | 49 | 9 (5-15) | 0.685 |
|  | Diabetes1 | 7 | 7 (5.5-14) |  | 9 | 6 (4-7) | 0.369 |
| BMI=1 | Smoking0 | 140 | 6 (3-13) |  | 164 | 5 (2-11) | 0.075 |
|  | Smoking1 | 68 | 4 (2-12) |  | 75 | 5 (2-10) | 0.589 |
| BMI=2 | Smoking0 | 570 | 7 (4-14) |  | 561 | 7 (4-12) | 0.525 |
|  | Smoking1 | 397 | 5 (2-11) |  | 357 | 5 (2-11) | 0.962 |
| BMI=3 | Smoking0 | 156 | 7 (4-12) |  | 158 | 7 (4-12) | 0.658 |
|  | Smoking1 | 99 | 7 (3-12) |  | 105 | 7 (3-14) | 0.355 |
| BMI=4 | Smoking0 | 34 | 9 (6.3-14) |  | 40 | 7 (4.8-14.3) | 0.868 |
|  | Smoking1 | 14 | 6.5 (4-16.3) | | 18 | 8 (4-14.8) | 0.885 |
| BMI=1 | Drinking0 | 150 | 5 (2-9.8) |  | 165 | 5 (2-10) | 0.389 |
|  | Drinking1 | 58 | 5 (2-17) |  | 74 | 6 (2-14) | 0.380 |
| BMI=2 | Drinking0 | 577 | 6 (3-13) |  | 563 | 7 (4-12) | 0.741 |
|  | Drinking1 | 390 | 6 (2-13) |  | 355 | 6 (2-11) | 0.210 |
| BMI=3 | Drinking0 | 160 | 7 (4-12) |  | 162 | 7 (4-12) | 0.512 |
|  | Drinking1 | 95 | 7 (4-12) |  | 101 | 8 (3-14) | 0.504 |
| BMI=4 | Drinking0 | 30 | 8.5 (6-13.8) | | 39 | 8 (5.5-14.5) | 0.678 |
|  | Drinking1 | 18 | 9.5 (4.3-16.3) | | 19 | 7 (4-15) | 0.553 |
| BMI=1 | District0 | 126 | 6 (3-17) |  | 149 | 6 (3-13) | 0.235 |
|  | District1 | 82 | 4 (2-7) |  | 90 | 3 (2-7) | 0.697 |
| BMI=2 | District0 | 643 | 6 (3-12) |  | 648 | 6 (3-11.3) | 0.851 |
|  | District1 | 324 | 7 (3-13) |  | 270 | 6 (3-12) | 0.215 |
| BMI=3 | District0 | 172 | 7 (4-11) |  | 182 | 7 (4-14) | 0.066 |
|  | District1 | 83 | 8 (4-14.5) |  | 81 | 7 (4-11) | 0.147 |
| BMI=4 | District0 | 28 | 9 (5.8-14.5) | | 47 | 7 (6-14.5) | 0.824 |
|  | District1 | 20 | 7 (6-14) |  | 11 | 4 (2-14) | 0.594 |
| BMI=1 | Education1 | 24 | 6.5 (4-13.5) | | 21 | 5 (3-8) | 0.141 |
|  | Education2 | 68 | 5 (2-12) |  | 77 | 5 (2-9) | 0.710 |
|  | Education3 | 61 | 5 (2-9) |  | 75 | 4 (2-10) | 0.329 |
|  | Education4 | 32 | 6 (2.8-13.8) | | 38 | 5 (2-12.8) | 0.289 |
|  | Education5 | 23 | 4 (2-9) |  | 28 | 9.5 (3.8-14.5) | 0.354 |
| BMI=2 | Education1 | 81 | 12 (7-20) |  | 68 | 7.5 (4-14) | 0.009 |
|  | Education2 | 355 | 5 (2-12) |  | 284 | 7 (3-13) | 0.219 |
|  | Education3 | 263 | 6 (3-10) |  | 308 | 5 (2.8-10) | 0.409 |
|  | Education4 | 136 | 6 (2-13) |  | 138 | 6 (4-11) | 0.804 |
|  | Education5 | 132 | 7 (4-13) |  | 120 | 7 (4-13) | 0.794 |
| BMI=3 | Education1 | 21 | 7 (4-18) |  | 21 | 8 (3-14) | 0.715 |
|  | Education2 | 74 | 8 (3-13) |  | 79 | 9 (4-15) | 0.249 |
|  | Education3 | 83 | 6 (4-10) |  | 81 | 7 (3-12) | 0.730 |
|  | Education4 | 46 | 7 (4-11.5) |  | 45 | 7 (5-11) | 0.822 |
|  | Education5 | 31 | 9 (6-11) |  | 37 | 7 (4-13) | 0.831 |
| BMI=4 | Education1 | 5 | 7 (7-9) |  | 4 | 8 (3.5-12.5) | 0.749 |
|  | Education2 | 12 | 7 (6-11.3) |  | 15 | 4 (2-11.5) | 0.624 |
|  | Education3 | 19 | 13 (5-17.5) | | 18 | 9.5 (4.3-13.8) | 0.332 |
|  | Education4 | 5 | 14 (11-16) | | 13 | 16 (7-17) | 0.832 |
|  | Education5 | 7 | 5 (4-7.5) |  | 8 | 7 (6-7.3) | 0.436 |
| LOS=1 | Surgery0 | 141 | 3 (2-5) |  | 162 | 3 (2-5) | 0.542 |
|  | Surgery1 | 612 | 3 (2-5) |  | 591 | 3 (2-5) | 0.911 |
| LOS=2 | Surgery0 | 86 | 9 (7-11) |  | 84 | 9 (7-10.3) | 0.461 |
|  | Surgery1 | 300 | 9 (7-11) |  | 321 | 9 (7-11) | 0.992 |
| LOS=3 | Surgery0 | 31 | 18 (15-21) | | 34 | 17 (15-19.8) | 0.645 |
|  | Surgery1 | 230 | 18 (15-21) | | 227 | 19 (15-22) | 0.343 |
| LOS=4 | Surgery0 | 11 | 36 (35.5-56.5) | | 4 | 33.5 (31.5-39.3) | 0.192 |
|  | Surgery1 | 67 | 36 (32-43.5) | | 55 | 38 (34-50) | 0.087 |
| LOS=1 | Diagnosis1 | 577 | 2 (2-4) |  | 575 | 3 (2-4) | 0.473 |
|  | Diagnosis2 | 31 | 4 (3-5) |  | 39 | 4 (3-6) | 0.735 |
|  | Diagnosis3 | 97 | 5 (3-6) |  | 89 | 4 (3-6) | 0.517 |
|  | Diagnosis4 | 10 | 5 (5-5) |  | 9 | 6 (4-6) | 0.861 |
|  | Diagnosis5 | 17 | 5 (4-6) |  | 16 | 5 (4-6) | 0.656 |
|  | Diagnosis6 | 21 | 5 (4-5) |  | 25 | 4 (3-6) | 0.408 |
| LOS=2 | Diagnosis1 | 168 | 9 (7-11) |  | 202 | 9 (7-11) | 0.855 |
|  | Diagnosis2 | 50 | 8.5 (7-11) |  | 33 | 9 (7-11) | 0.479 |
|  | Diagnosis3 | 88 | 9 (8-11) |  | 100 | 9 (7-11) | 0.555 |
|  | Diagnosis4 | 19 | 9 (7-11) |  | 13 | 8 (7-10) | 0.574 |
|  | Diagnosis5 | 25 | 8 (7-10) |  | 24 | 9 (7-11.3) | 0.280 |
|  | Diagnosis6 | 36 | 9 (8-10.3) |  | 33 | 8 (8-11) | 0.568 |
| LOS=3 | Diagnosis1 | 156 | 18 (15-22.3) | | 159 | 19 (16-22) | 0.619 |
|  | Diagnosis2 | 16 | 17 (15-19.3) | | 22 | 16 (15-18) | 0.443 |
|  | Diagnosis3 | 49 | 16 (14-19) | | 43 | 16 (14.5-19) | 0.522 |
|  | Diagnosis4 | 1 | 14 (14-14) | | 3 | 19 (17.5-23.5) | NA |
|  | Diagnosis5 | 12 | 19.5 (17-21) | | 11 | 23 (18-24.5) | 0.217 |
|  | Diagnosis6 | 27 | 18 (17.5-23) | | 23 | 19 (15-21.5) | 0.760 |
| LOS=4 | Diagnosis1 | 54 | 36.5 (33-43.8) | | 40 | 37.5 (33.8-52) | 0.532 |
|  | Diagnosis2 | 2 | 33 (32-34) | | 4 | 48.5 (39.8-55.3) | NA |
|  | Diagnosis3 | 10 | 34 (32.3-38.5) | | 7 | 43 (37-71.5) | 0.214 |
|  | Diagnosis4 | 0 | NA |  | 0 | NA | NA |
|  | Diagnosis5 | 8 | 39 (33-42.8) | | 4 | 37.5 (35.5-39.8) | 0.582 |
|  | Diagnosis6 | 4 | 45 (41.5-45.3) | | 4 | 37.5 (35.8-38.3) | 0.249 |
| LOS=1 | Hypertension0 | 524 | 3 (2-5) |  | 538 | 3 (2-5) | 0.670 |
|  | Hypertension1 | 229 | 3 (2-5) |  | 215 | 3 (1-5) | 0.982 |
| LOS=2 | Hypertension0 | 261 | 9 (7-11) |  | 271 | 9 (7-11) | 0.636 |
|  | Hypertension1 | 125 | 9 (7-11) |  | 134 | 9 (7-11) | 0.952 |
| LOS=3 | Hypertension0 | 173 | 18 (15-21) | | 154 | 18.5 (16-22) | 0.128 |
|  | Hypertension1 | 88 | 18 (15.8-22.3) | | 107 | 18 (15-21) | 0.426 |
| LOS=4 | Hypertension0 | 49 | 37 (33-44) | | 41 | 38 (34-52) | 0.118 |
|  | Hypertension1 | 29 | 36 (33-49) | | 18 | 38 (32.3-44.5) | 0.972 |
| LOS=1 | Diabetes0 | 643 | 3 (2-5) |  | 669 | 3 (2-5) | 0.884 |
|  | Diabetes1 | 110 | 3 (2-4) |  | 84 | 3 (1.8-4.3) | 0.659 |
| LOS=2 | Diabetes0 | 341 | 9 (7-11) |  | 358 | 9 (7-11) | 0.979 |
|  | Diabetes1 | 45 | 9 (8-11) |  | 47 | 9 (7-10.5) | 0.230 |
| LOS=3 | Diabetes0 | 228 | 18 (15-21) | | 227 | 18 (15-21.5) | 0.890 |
|  | Diabetes1 | 33 | 18 (15-20) | | 34 | 20 (16-23.8) | 0.051 |
| LOS=4 | Diabetes0 | 69 | 36 (33-45) | | 48 | 40 (34-54.8) | 0.107 |
|  | Diabetes1 | 9 | 36 (35-41) | | 11 | 32 (31.5-36.5) | 0.235 |
| LOS=1 | Smoking0 | 428 | 4 (2-5) |  | 451 | 3 (2-5) | 0.777 |
|  | Smoking1 | 325 | 3 (2-4) |  | 302 | 3 (2-4) | 0.469 |
| LOS=2 | Smoking0 | 251 | 9 (7-11) |  | 280 | 9 (7-11) | 0.505 |
|  | Smoking1 | 135 | 9 (8-11) |  | 125 | 9 (7-10) | 0.111 |
| LOS=3 | Smoking0 | 171 | 18 (15-21) | | 158 | 19 (15-22) | 0.083 |
|  | Smoking1 | 90 | 18 (15.3-22.8) | | 103 | 17 (15-20.5) | 0.268 |
| LOS=4 | Smoking0 | 50 | 36 (33-45) | | 34 | 38 (34-52) | 0.476 |
|  | Smoking1 | 28 | 36.5 (30.8-42.3) | | 25 | 38 (33-43) | 0.218 |
| LOS=1 | Drinking0 | 465 | 3 (2-5) |  | 461 | 3 (2-5) | 0.806 |
|  | Drinking1 | 288 | 3 (2-4) |  | 292 | 3 (2-4) | 0.724 |
| LOS=2 | Drinking0 | 244 | 9 (7-11) |  | 275 | 9 (7-11) | 0.667 |
|  | Drinking1 | 142 | 9 (7-11) |  | 130 | 9 (7-11) | 0.266 |
| LOS=3 | Drinking0 | 165 | 18 (15-21) | | 159 | 19 (16-22.5) | 0.046 |
|  | Drinking1 | 96 | 18 (15-21.3) | | 102 | 17 (15-20) | 0.128 |
| LOS=4 | Drinking0 | 43 | 37 (34-45) | | 34 | 41.5 (34.3-56.3) | 0.100 |
|  | Drinking1 | 35 | 36 (31-43.5) | | 25 | 37 (33-43) | 0.955 |
| LOS=1 | District0 | 258 | 3 (2-5) |  | 244 | 3 (2-4) | 0.801 |
|  | District1 | 495 | 3 (2-5) |  | 509 | 3 (2-5) | 0.782 |
| LOS=2 | District0 | 134 | 9 (7-11) |  | 120 | 9 (7-11) | 0.418 |
|  | District1 | 252 | 9 (7-11) |  | 285 | 9 (7-11) | 0.897 |
| LOS=3 | District0 | 93 | 18 (16-21) | | 79 | 18 (15.5-22) | 0.584 |
|  | District1 | 168 | 18 (15-21) | | 182 | 18 (15-22) | 0.612 |
| LOS=4 | District0 | 24 | 35.5 (33-45) | | 9 | 34 (32-38) | 0.709 |
|  | District1 | 54 | 36.5 (33-43.8) | | 50 | 39 (34-52) | 0.259 |
| LOS=1 | Education1 | 42 | 4 (2-5) |  | 54 | 3.5 (2-5) | 0.720 |
|  | Education2 | 277 | 3 (2-5) |  | 224 | 3 (2-5) | 0.238 |
|  | Education3 | 229 | 3 (2-5) |  | 275 | 3 (2-4) | 0.434 |
|  | Education4 | 114 | 3 (2-5) |  | 112 | 4 (2-5) | 0.482 |
|  | Education5 | 91 | 4 (2-5) |  | 88 | 3.5 (2-5) | 0.426 |
| LOS=2 | Education1 | 37 | 9 (7-11) |  | 31 | 10 (8-11.5) | 0.271 |
|  | Education2 | 119 | 9 (7-11) |  | 122 | 9 (8-10) | 0.250 |
|  | Education3 | 114 | 9 (7-10) |  | 119 | 9 (7-11) | 0.441 |
|  | Education4 | 56 | 9 (7-11.3) |  | 73 | 9 (7-11) | 0.723 |
|  | Education5 | 60 | 9 (7-11) |  | 60 | 8.5 (7-11) | 0.659 |
| LOS=3 | Education1 | 40 | 19 (16-21) | | 27 | 17 (15-22.5) | NA |
|  | Education2 | 92 | 18 (15.8-21.3) | | 89 | 19 (15-22) | 0.976 |
|  | Education3 | 58 | 17.5 (15-21.8) | | 71 | 19 (16.5-22) | 0.613 |
|  | Education4 | 40 | 17 (14.8-20) | | 40 | 17 (15-21) | 0.790 |
|  | Education5 | 31 | 17 (15-19.5) | | 34 | 17 (15-21.8) | 0.740 |
| LOS=4 | Education1 | 12 | 36.5 (34.5-39) | | 2 | 37 (36.5-37.5) | 0.657 |
|  | Education2 | 21 | 36 (33-46) | | 20 | 44 (33.8-58.3) | 0.285 |
|  | Education3 | 25 | 36 (32-45) | | 17 | 35 (33-45) | 0.946 |
|  | Education4 | 9 | 41 (35-53) | | 9 | 37 (34-42) | 0.378 |
|  | Education5 | 11 | 34 (32.5-37.5) | | 11 | 39 (35-50) | 0.101 |
| Surgery=0 | Diagnosis1 | 146 | 4 (2-8) |  | 157 | 4 (2-7) | 0.356 |
|  | Diagnosis2 | 17 | 7 (3-8) |  | 25 | 7 (6-12) | 0.174 |
|  | Diagnosis3 | 50 | 8.5 (5.3-12.8) | | 49 | 7 (5-11) | 0.473 |
|  | Diagnosis4 | 2 | 5 (5-5) |  | 1 | 3 (3-3) | NA |
|  | Diagnosis5 | 29 | 8 (7-11) |  | 25 | 7 (6-9) | 0.061 |
|  | Diagnosis6 | 25 | 9 (4-13) |  | 27 | 8 (6.5-14.5) | 0.293 |
| Surgery=1 | Diagnosis1 | 809 | 5 (2-12) |  | 819 | 5 (2-12) | 0.771 |
|  | Diagnosis2 | 82 | 8 (6-13) |  | 73 | 7 (5-14) | 0.589 |
|  | Diagnosis3 | 194 | 8 (5-14) |  | 190 | 7 (5-13) | 0.774 |
|  | Diagnosis4 | 28 | 7 (6-10.3) |  | 24 | 7.5 (6-10) | 0.414 |
|  | Diagnosis5 | 33 | 10 (6-20) |  | 30 | 12.5 (7-23) | 0.656 |
|  | Diagnosis6 | 63 | 12 (7-18.5) | | 58 | 9 (6-16.5) | 0.180 |
| Surgery=0 | Hypertension0 | 166 | 6 (3-10) |  | 185 | 6 (3-9) | 0.084 |
|  | Hypertension1 | 103 | 6 (3-11) |  | 99 | 6 (3-10) | 0.350 |
| Surgery=1 | Hypertension0 | 841 | 6 (3-13) |  | 819 | 6 (3-12) | 0.649 |
|  | Hypertension1 | 368 | 7 (3-14) |  | 375 | 7 (3-14) | 0.806 |
| Surgery=0 | Diabetes0 | 214 | 7 (3-11) |  | 246 | 6 (3-9) | 0.116 |
|  | Diabetes1 | 55 | 4 (3-9) |  | 38 | 6 (4-10.5) | 0.743 |
| Surgery=1 | Diabetes0 | 1067 | 6 (3-13) |  | 1056 | 6.5 (3-13) | 0.863 |
|  | Diabetes1 | 142 | 6 (2-12.8) |  | 138 | 7 (3-14.8) | 0.197 |
| Surgery=0 | Smoking0 | 159 | 6 (3-10.5) |  | 166 | 6 (3-10) | 0.234 |
|  | Smoking1 | 110 | 5.5 (3-10.8) | | 118 | 6 (3-9) | 0.086 |
| Surgery=1 | Smoking0 | 741 | 7 (4-14) |  | 757 | 7 (4-13) | 0.397 |
|  | Smoking1 | 468 | 5 (2-12) |  | 437 | 6 (2-14) | 0.156 |
| Surgery=0 | Drinking0 | 163 | 6 (3-10) |  | 168 | 6 (3-10) | 0.278 |
|  | Drinking1 | 106 | 6.5 (3-12) |  | 116 | 5 (3-9) | 0.056 |
| Surgery=1 | Drinking0 | 754 | 6.5 (4-13) |  | 761 | 7 (3-13) | 0.495 |
|  | Drinking1 | 455 | 6 (2-13.5) |  | 433 | 6 (3-14) | 0.672 |
| Surgery=0 | District0 | 89 | 6 (4-9) |  | 88 | 6 (2.8-9) | 0.272 |
|  | District1 | 180 | 6 (3-11) |  | 196 | 6 (3-9.3) | 0.600 |
| Surgery=1 | District0 | 420 | 6 (3-14) |  | 364 | 6 (3-11) | 0.376 |
|  | District1 | 789 | 6 (3-13) |  | 830 | 7 (3-13) | 0.280 |
| Surgery=0 | Education1 | 20 | 7.5 (5.5-13.5) | | 26 | 6 (4-7) | 0.054 |
|  | Education2 | 111 | 6 (3-9) |  | 95 | 7 (3-9) | 0.399 |
|  | Education3 | 67 | 4 (2-8) |  | 90 | 4.5 (2.3-7) | 0.524 |
|  | Education4 | 38 | 7 (4-12.8) |  | 37 | 5 (2-8) | 0.063 |
|  | Education5 | 33 | 8 (6-13) |  | 36 | 6.5 (4-11.3) | 0.316 |
| Surgery=1 | Education1 | 111 | 11 (5-19) |  | 88 | 8 (3.8-14) | 0.027 |
|  | Education2 | 398 | 6 (2-13.8) |  | 360 | 7 (3-14) | 0.262 |
|  | Education3 | 359 | 6 (3-11) |  | 392 | 6 (3-11) | 0.328 |
|  | Education4 | 181 | 6 (3-13) |  | 197 | 7 (4-13) | 0.184 |
|  | Education5 | 160 | 6 (4-11.3) |  | 157 | 7 (4-13) | 0.305 |
| Diagnosis=1 | Hypertension0 | 649 | 5 (2-11) |  | 671 | 5 (2-11) | 0.988 |
|  | Hypertension1 | 306 | 5 (2-14) |  | 305 | 5 (2-12) | 0.426 |
| Diagnosis=2 | Hypertension0 | 62 | 8 (5.3-12) |  | 66 | 7 (4-12.5) | 0.548 |
|  | Hypertension1 | 37 | 8 (6-11) |  | 32 | 9 (6-14.3) | 0.614 |
| Diagnosis=3 | Hypertension0 | 178 | 8 (5-12) |  | 159 | 7 (5-11) | 0.487 |
|  | Hypertension1 | 66 | 8 (6-15.5) |  | 80 | 10 (6-14) | 0.264 |
| Diagnosis=4 | Hypertension0 | 18 | 7 (5-8.8) |  | 16 | 7.5 (6-11.3) | 0.163 |
|  | Hypertension1 | 12 | 8.5 (7-11) |  | 9 | 7 (6-9) | 0.208 |
| Diagnosis=5 | Hypertension0 | 44 | 9.5 (7-20.3) | | 40 | 8 (6-12) | 0.065 |
|  | Hypertension1 | 18 | 7 (6-10) |  | 15 | 14 (6.5-24.5) | 0.061 |
| Diagnosis=6 | Hypertension0 | 56 | 11 (7-18) |  | 52 | 8 (6-13) | 0.108 |
|  | Hypertension1 | 32 | 9 (6-14) |  | 33 | 10 (7-18) | 0.289 |
| Diagnosis=1 | Diabetes0 | 821 | 5 (2-12) |  | 854 | 5 (2-11) | 0.338 |
|  | Diabetes1 | 134 | 4 (2-10) |  | 122 | 5 (2-11.8) | 0.210 |
| Diagnosis=2 | Diabetes0 | 83 | 8 (6-12) |  | 89 | 7 (5-14) | 0.554 |
|  | Diabetes1 | 16 | 8 (4-10.8) |  | 9 | 9 (6-11) | 0.586 |
| Diagnosis=3 | Diabetes0 | 217 | 8 (5-13) |  | 213 | 7 (5-13) | 0.690 |
|  | Diabetes1 | 27 | 10 (6-14) |  | 26 | 7 (4.5-11) | 0.434 |
| Diagnosis=4 | Diabetes0 | 26 | 7 (5.3-10) |  | 23 | 7 (6-10) | 0.611 |
|  | Diabetes1 | 4 | 7.5 (5.8-10.3) | | 2 | 14 (11.5-16.5) | NA |
| Diagnosis=5 | Diabetes0 | 56 | 9 (6-18.3) |  | 48 | 9 (6-13.3) | 0.433 |
|  | Diabetes1 | 6 | 8 (4.8-10.5) | | 7 | 8 (5.5-19) | 0.447 |
| Diagnosis=6 | Diabetes0 | 78 | 10 (7-18) |  | 75 | 8 (6-15) | 0.141 |
|  | Diabetes1 | 10 | 9.5 (6.5-16.3) | | 10 | 14 (11.3-23.8) | 0.121 |
| Diagnosis=1 | Smoking0 | 525 | 5 (2-13) |  | 555 | 5 (3-11) | 0.180 |
|  | Smoking1 | 430 | 4 (2-10) |  | 421 | 4 (2-11) | 0.424 |
| Diagnosis=2 | Smoking0 | 74 | 8 (6-13) |  | 72 | 7 (5-13.3) | 0.601 |
|  | Smoking1 | 25 | 8 (7-9) |  | 26 | 7.5 (5.3-15) | 0.556 |
| Diagnosis=3 | Smoking0 | 185 | 7 (5-13) |  | 193 | 7 (5-12) | 0.519 |
|  | Smoking1 | 59 | 9 (6-14) |  | 46 | 7.5 (5.3-13.8) | 0.424 |
| Diagnosis=4 | Smoking0 | 23 | 8 (6-10.5) |  | 15 | 7 (6.5-9.5) | 0.548 |
|  | Smoking1 | 7 | 7 (5-7) |  | 10 | 7 (6-10) | 0.425 |
| Diagnosis=5 | Smoking0 | 40 | 10 (7-19.3) | | 43 | 10 (6.5-15) | 0.503 |
|  | Smoking1 | 22 | 7 (5.3-10.8) | | 12 | 7 (6-10.3) | 0.687 |
| Diagnosis=6 | Smoking0 | 53 | 10 (7-17) |  | 45 | 7 (6-13) | 0.049 |
|  | Smoking1 | 35 | 10 (7.5-18) | | 40 | 12 (7.8-18.3) | 0.372 |
| Diagnosis=1 | Drinking0 | 553 | 5 (2-11) |  | 576 | 5 (2-11) | 0.895 |
|  | Drinking1 | 402 | 5 (2-12) |  | 400 | 4 (2-11) | 0.381 |
| Diagnosis=2 | Drinking0 | 75 | 8 (6-13) |  | 66 | 7 (5-12.8) | 0.907 |
|  | Drinking1 | 24 | 7 (4-9.3) |  | 32 | 9 (5-15) | 0.158 |
| Diagnosis=3 | Drinking0 | 172 | 8 (5-13) |  | 173 | 7 (5-11) | 0.647 |
|  | Drinking1 | 72 | 8 (5.8-13.3) | | 66 | 8.5 (6-14) | 0.625 |
| Diagnosis=4 | Drinking0 | 23 | 8 (6.5-11) |  | 13 | 8 (7-10) | 0.510 |
|  | Drinking1 | 7 | 5 (5-7) |  | 12 | 6.5 (6-10) | 0.104 |
| Diagnosis=5 | Drinking0 | 38 | 9.5 (6-18.8) | | 41 | 9 (6-15) | 0.457 |
|  | Drinking1 | 24 | 7.5 (6.8-17) | | 14 | 8.5 (6-13.5) | 0.995 |
| Diagnosis=6 | Drinking0 | 56 | 9 (6-14.3) |  | 60 | 8 (6-15) | 0.893 |
|  | Drinking1 | 32 | 13 (9-19.5) | | 25 | 10 (7-17) | 0.267 |
| Diagnosis=1 | District0 | 346 | 5 (2-11) |  | 314 | 4 (2-9) | 0.204 |
|  | District1 | 609 | 5 (2-12) |  | 662 | 5 (2-12) | 0.976 |
| Diagnosis=2 | District0 | 30 | 8 (7-12.8) |  | 30 | 8 (6-15.8) | 0.416 |
|  | District1 | 69 | 8 (5-11) |  | 68 | 7 (5-12) | 0.636 |
| Diagnosis=3 | District0 | 73 | 10 (6-14) |  | 64 | 7 (5-13) | 0.220 |
|  | District1 | 171 | 7 (5-12) |  | 175 | 8 (5-12) | 0.553 |
| Diagnosis=4 | District0 | 5 | 9 (8-11) |  | 6 | 10 (7.8-11.5) | 0.417 |
|  | District1 | 25 | 7 (5-10) |  | 19 | 7 (6-8.5) | 0.857 |
| Diagnosis=5 | District0 | 24 | 8.5 (7-20.3) | | 14 | 7.5 (6-10) | 0.045 |
|  | District1 | 38 | 9 (6-16.5) |  | 41 | 10 (6-21) | 0.707 |
| Diagnosis=6 | District0 | 31 | 9 (6.5-15.5) | | 24 | 8 (6.8-12.3) | 0.459 |
|  | District1 | 57 | 11 (7-18) |  | 61 | 9 (6-15) | 0.516 |
| Diagnosis=1 | Education1 | 81 | 9 (4-20) |  | 63 | 5 (3-13) | 0.005 |
|  | Education2 | 346 | 4 (2-10) |  | 315 | 5 (2-10) | 0.155 |
|  | Education3 | 290 | 4 (2-10) |  | 341 | 4 (2-10) | 0.320 |
|  | Education4 | 140 | 5 (2-12) |  | 153 | 5 (3-11) | 0.875 |
|  | Education5 | 98 | 6 (3-11) |  | 104 | 6 (3-12) | 0.598 |
| Diagnosis=2 | Education1 | 9 | 11 (9-17) |  | 13 | 12 (6-14) | 0.399 |
|  | Education2 | 36 | 8 (6-12.3) |  | 35 | 9 (7-15) | 0.093 |
|  | Education3 | 19 | 7 (4.5-9) |  | 28 | 6 (3-10.8) | 0.432 |
|  | Education4 | 17 | 8 (6-14) |  | 10 | 5 (4-8) | 0.154 |
|  | Education5 | 18 | 7 (5.3-12.8) | | 12 | 7 (5.8-8.8) | 0.210 |
| Diagnosis=3 | Education1 | 24 | 9.5 (6-14) |  | 23 | 10 (6.5-13) | 0.871 |
|  | Education2 | 63 | 10 (5-15.5) | | 69 | 9 (6-13) | 0.723 |
|  | Education3 | 63 | 8 (6-12) |  | 66 | 7 (5-10) | 0.258 |
|  | Education4 | 39 | 7 (4.5-11) |  | 39 | 7 (5-11.5) | 0.521 |
|  | Education5 | 55 | 7 (4-10.5) |  | 42 | 7 (5-13.8) | 0.781 |
| Diagnosis=4 | Education1 | 6 | 10 (7.5-11) | | 2 | 9 (8.5-9.5) | NA |
|  | Education2 | 7 | 7 (6-9.5) |  | 4 | 8 (6.8-9.3) | 0.928 |
|  | Education3 | 10 | 7 (5.3-8.8) | | 8 | 7.5 (6-10.5) | 0.326 |
|  | Education4 | 6 | 6.5 (5.3-10.8) | | 5 | 7 (6-11) | 0.700 |
|  | Education5 | 1 | 5 (5-5) |  | 6 | 6.5 (4.5-7.8) | NA |
| Diagnosis=5 | Education1 | 5 | 15 (8-18) |  | 6 | 6.5 (6-8.5) | 0.127 |
|  | Education2 | 29 | 8 (6-21) |  | 11 | 8 (5.5-13) | 0.241 |
|  | Education3 | 14 | 7 (6-9) |  | 19 | 10 (7-14) | 0.566 |
|  | Education4 | 6 | 16.5 (11.5-28.3) | | 6 | 10.5 (5.8-20.5) | 0.274 |
|  | Education5 | 8 | 8.5 (7-10.3) | | 13 | 12 (7-22) | 0.105 |
| Diagnosis=6 | Education1 | 6 | 8 (4.3-11.8) | | 7 | 10 (6-16) | 0.617 |
|  | Education2 | 28 | 9.5 (7-18) |  | 21 | 13 (8-19) | 0.871 |
|  | Education3 | 30 | 9 (7-13.8) |  | 20 | 8 (4-12) | 0.087 |
|  | Education4 | 11 | 9 (5.5-17.5) | | 21 | 8 (7-15) | 0.729 |
|  | Education5 | 13 | 14 (10-18) | | 16 | 10.5 (3.8-16) | 0.393 |
| Hypertension=0 | Diabetes0 | 923 | 6 (3-12) |  | 942 | 6 (3-11) | 0.428 |
|  | Diabetes1 | 84 | 6 (2-11) |  | 62 | 6.5 (4-11.8) | 0.112 |
| Hypertension=1 | Diabetes0 | 358 | 7 (3-14) |  | 360 | 7 (3-14) | 0.455 |
|  | Diabetes1 | 113 | 5 (2-13) |  | 114 | 7 (3-14) | 0.635 |
| Hypertension=0 | Smoking0 | 635 | 6 (4-12) |  | 648 | 6 (3-11) | 0.565 |
|  | Smoking1 | 372 | 5 (3-12) |  | 356 | 5.5 (2.8-11) | 0.879 |
| Hypertension=1 | Smoking0 | 265 | 7 (5-15) |  | 275 | 7 (4-14) | 0.177 |
|  | Smoking1 | 206 | 4.5 (2-11) |  | 199 | 7 (2-14) | 0.381 |
| Hypertension=0 | Drinking0 | 644 | 6 (3-12) |  | 649 | 6 (3-11) | 0.935 |
|  | Drinking1 | 363 | 6 (3-13) |  | 355 | 6 (3-11.5) | 0.457 |
| Hypertension=1 | Drinking0 | 273 | 7 (4-13) |  | 280 | 7 (4-14) | 0.832 |
|  | Drinking1 | 198 | 7 (2-13.8) |  | 194 | 7 (3-14) | 0.418 |
| Hypertension=0 | District0 | 367 | 6 (3-12) |  | 339 | 6 (3-11) | 0.185 |
|  | District1 | 640 | 6 (3-12) |  | 665 | 6 (3-11) | 0.764 |
| Hypertension=1 | District0 | 142 | 7 (3-14) |  | 113 | 7 (3-11) | 0.110 |
|  | District1 | 329 | 6 (3-13) |  | 361 | 7 (3-14) | 0.906 |
| Hypertension=0 | Education1 | 83 | 8 (4-19) |  | 68 | 6 (3-13) | 0.070 |
|  | Education2 | 306 | 6 (3-12) |  | 295 | 6 (3-11) | 0.315 |
|  | Education3 | 311 | 6 (3-10) |  | 339 | 6 (3-10) | 0.656 |
|  | Education4 | 153 | 6 (3-12) |  | 164 | 6 (3-12) | 0.665 |
|  | Education5 | 154 | 7 (4-11.8) |  | 138 | 7 (4-11) | 0.887 |
| Hypertension=1 | Education1 | 48 | 11.5 (7-18) | | 46 | 8 (5-14) | 0.030 |
|  | Education2 | 203 | 6 (2-12) |  | 160 | 8 (3-15) | 0.344 |
|  | Education3 | 115 | 6 (3-11) |  | 143 | 6 (2-11) | 0.127 |
|  | Education4 | 66 | 7 (3-13.8) |  | 70 | 7 (5-12.8) | 0.598 |
|  | Education5 | 39 | 7 (3.5-14) |  | 55 | 10 (4.5-15) | 0.545 |
| Diabetes=0 | Smoking0 | 796 | 7 (4-14) |  | 820 | 7 (4-12) | 0.103 |
|  | Smoking1 | 485 | 5 (2-12) |  | 482 | 6 (2-12) | 0.722 |
| Diabetes=1 | Smoking0 | 104 | 6 (3-11) |  | 103 | 7 (4-12) | 0.278 |
|  | Smoking1 | 93 | 4 (2-13) |  | 73 | 7 (2-14) | 0.364 |
| Diabetes=0 | Drinking0 | 798 | 7 (4-13) |  | 814 | 6 (3-12) | 0.565 |
|  | Drinking1 | 483 | 6 (3-13) |  | 488 | 6 (3-13) | 0.319 |
| Diabetes=1 | Drinking0 | 119 | 5 (3-10) |  | 115 | 7 (4-14) | 0.024 |
|  | Drinking1 | 78 | 7.5 (2-14) |  | 61 | 6 (2-13) | 0.656 |
| Diabetes=0 | District0 | 456 | 6 (3-13) |  | 406 | 6 (3-11) | 0.133 |
|  | District1 | 825 | 7 (3-12) |  | 896 | 6.5 (3-12) | 0.958 |
| Diabetes=1 | District0 | 53 | 6 (3-11) |  | 46 | 6.5 (3-15.5) | 0.156 |
|  | District1 | 144 | 5 (2-13) |  | 130 | 7 (3-12.8) | 0.414 |
| Diabetes=0 | Education1 | 110 | 11 (5-19) |  | 104 | 7 (4-14) | 0.025 |
|  | Education2 | 428 | 6 (3-13) |  | 388 | 6 (3-13) | 0.479 |
|  | Education3 | 380 | 6 (3-11) |  | 439 | 6 (3-11) | 0.124 |
|  | Education4 | 189 | 6 (3-13) |  | 201 | 7 (4-12) | 0.729 |
|  | Education5 | 174 | 7 (4-12) |  | 170 | 7 (4-13) | 0.568 |
| Diabetes=1 | Education1 | 21 | 9 (4-14) |  | 10 | 5.5 (2.3-6) | 0.045 |
|  | Education2 | 81 | 5 (2-11) |  | 67 | 8 (3-17.5) | 0.057 |
|  | Education3 | 46 | 3.5 (2-8.8) | | 43 | 5 (2-8.5) | 0.402 |
|  | Education4 | 30 | 7 (4.3-13) |  | 33 | 7 (4-13) | 0.687 |
|  | Education5 | 19 | 6 (4-14.5) |  | 23 | 8 (3-12.5) | 0.721 |
| Smoking=0 | Drinking0 | 771 | 7 (4-13) |  | 790 | 7 (4-11.8) | 0.542 |
|  | Drinking1 | 129 | 7 (4-15) |  | 133 | 7 (4-14) | 0.117 |
| Smoking=1 | Drinking0 | 146 | 5 (2-9) |  | 139 | 6 (3-14) | 0.046 |
|  | Drinking1 | 432 | 5 (2-12) |  | 416 | 6 (2-11) | 0.752 |
| Smoking=0 | District0 | 301 | 7 (4-14) |  | 269 | 6 (3-12) | 0.031 |
|  | District1 | 599 | 7 (4-13) |  | 654 | 7 (4-12) | 0.730 |
| Smoking=1 | District0 | 208 | 5 (2-11) |  | 183 | 5 (3-9) | 0.597 |
|  | District1 | 370 | 5 (2-12) |  | 372 | 6 (2-13.3) | 0.329 |
| Smoking=0 | Education1 | 105 | 9 (6-18) |  | 93 | 7 (4-14) | 0.035 |
|  | Education2 | 276 | 6 (3-14) |  | 276 | 7 (4-13.3) | 0.624 |
|  | Education3 | 233 | 6 (3-10) |  | 272 | 6 (3-10) | 0.246 |
|  | Education4 | 134 | 6 (4-13) |  | 144 | 6 (4-11.3) | 0.533 |
|  | Education5 | 152 | 7 (4-13) |  | 138 | 7 (4-12) | 0.960 |
| Smoking=1 | Education1 | 26 | 14 (4-22.3) | | 21 | 6 (3-9) | 0.176 |
|  | Education2 | 233 | 5 (2-11) |  | 179 | 5 (2-10.5) | 0.215 |
|  | Education3 | 193 | 5 (3-10) |  | 210 | 5 (2-11) | 0.552 |
|  | Education4 | 85 | 5 (2-13) |  | 90 | 7 (4-13.8) | 0.580 |
|  | Education5 | 41 | 5 (3-9) |  | 55 | 6 (2.5-14) | 0.173 |
| Drinking=0 | District0 | 307 | 7 (3.5-14) |  | 292 | 6 (3-11.3) | 0.182 |
|  | District1 | 610 | 6 (3-12) |  | 637 | 7 (4-12) | 0.356 |
| Drinking=1 | District0 | 202 | 6 (2-12) |  | 160 | 5.5 (3-9) | 0.553 |
|  | District1 | 359 | 6 (3-14) |  | 389 | 6 (3-14) | 0.562 |
| Drinking=0 | Education1 | 102 | 9 (5-18) |  | 87 | 7 (4-13.5) | 0.040 |
|  | Education2 | 291 | 6 (3-13) |  | 278 | 7 (4-14) | 0.033 |
|  | Education3 | 248 | 6 (3-10) |  | 272 | 6 (3-10) | 0.749 |
|  | Education4 | 136 | 6 (3-12) |  | 154 | 7 (4-11) | 0.913 |
|  | Education5 | 140 | 7 (4-12) |  | 138 | 7 (4-12) | 0.904 |
| Drinking=1 | Education1 | 29 | 12 (5-19) |  | 27 | 6 (3.5-14.5) | 0.141 |
|  | Education2 | 218 | 5 (2-12) |  | 177 | 5 (2-10) | 0.699 |
|  | Education3 | 178 | 6 (3-13) |  | 210 | 6 (2-12) | 0.140 |
|  | Education4 | 83 | 7 (3-13.5) |  | 80 | 7 (4-14) | 0.875 |
|  | Education5 | 53 | 6 (3-11) |  | 55 | 7 (3-14) | 0.317 |
| District=0 | Education1 | 58 | 7 (5-18) |  | 55 | 6 (3-13.5) | 0.096 |
|  | Education2 | 228 | 6 (3-13) |  | 167 | 6 (3-11.5) | 0.779 |
|  | Education3 | 169 | 6 (3-11) |  | 164 | 5 (2-9) | 0.047 |
|  | Education4 | 34 | 6 (2.3-13.8) | | 54 | 7 (3-11) | 0.957 |
|  | Education5 | 20 | 6.5 (3-11.5) | | 12 | 8.5 (6.3-13.8) | 0.213 |
| District=1 | Education1 | 73 | 11 (7-19) |  | 59 | 8 (4-13.5) | 0.014 |
|  | Education2 | 281 | 5 (2-12) |  | 288 | 7 (3-13.3) | 0.147 |
|  | Education3 | 257 | 6 (3-10) |  | 318 | 6 (3-11) | 0.882 |
|  | Education4 | 185 | 6 (3-13) |  | 180 | 7 (4-12) | 0.948 |
|  | Education5 | 173 | 7 (4-12) |  | 181 | 7 (4-13) | 0.703 |
| Definition of subgroup labels: Sex: 1 [Male], 2 [Female]; Age: 1 [<51years], 2 [51-59years], 3 [60-67years], 4 [≥68years]; BMI 1 [<18.5], 2 [18.5-24], 3 [24-28], 4 [≥28]; Length of Stay (LOS): 1 [1-6 days], 2 [7-13 days], 3 [14-29 days], 4 [≥30 days]; Surgery: 0 [No], 1 [Yes]; Diagnosis: 1 [Benign tumor], 2 [Malignant tumor], 3 [Inflammation], 4 [Hernia], 5 [Intestinal obstruction], 6 [Others]; Hypertension: 0 [No], 1 [Yes]; Diabetes: 0 [No], 1 [Yes]; Smoking: 0 [No], 1 [Yes]; Drinking: 0 [No], 1 [Yes]; District: 0 [Rural], 1 [Urban]; Education: 1 [Illiterate], 2 [Primary school], 3 [Middle school], 4 [High school], 5 [University degree]; | | | | | | | |
| *p* values are determined by T-test or Wilcoxon Rank Sum test. | | | |  |  |  |  |
